# Supplementary figures and images for: Two RNA-binding proteins mediate the sorting of miR223 from mitochondria into exosomes
Source: eLife. 2023 Jul 25;12:e85878. doi: 10.7554/eLife.85878 (PMC10403255; doi:10.7554/eLife.85878)

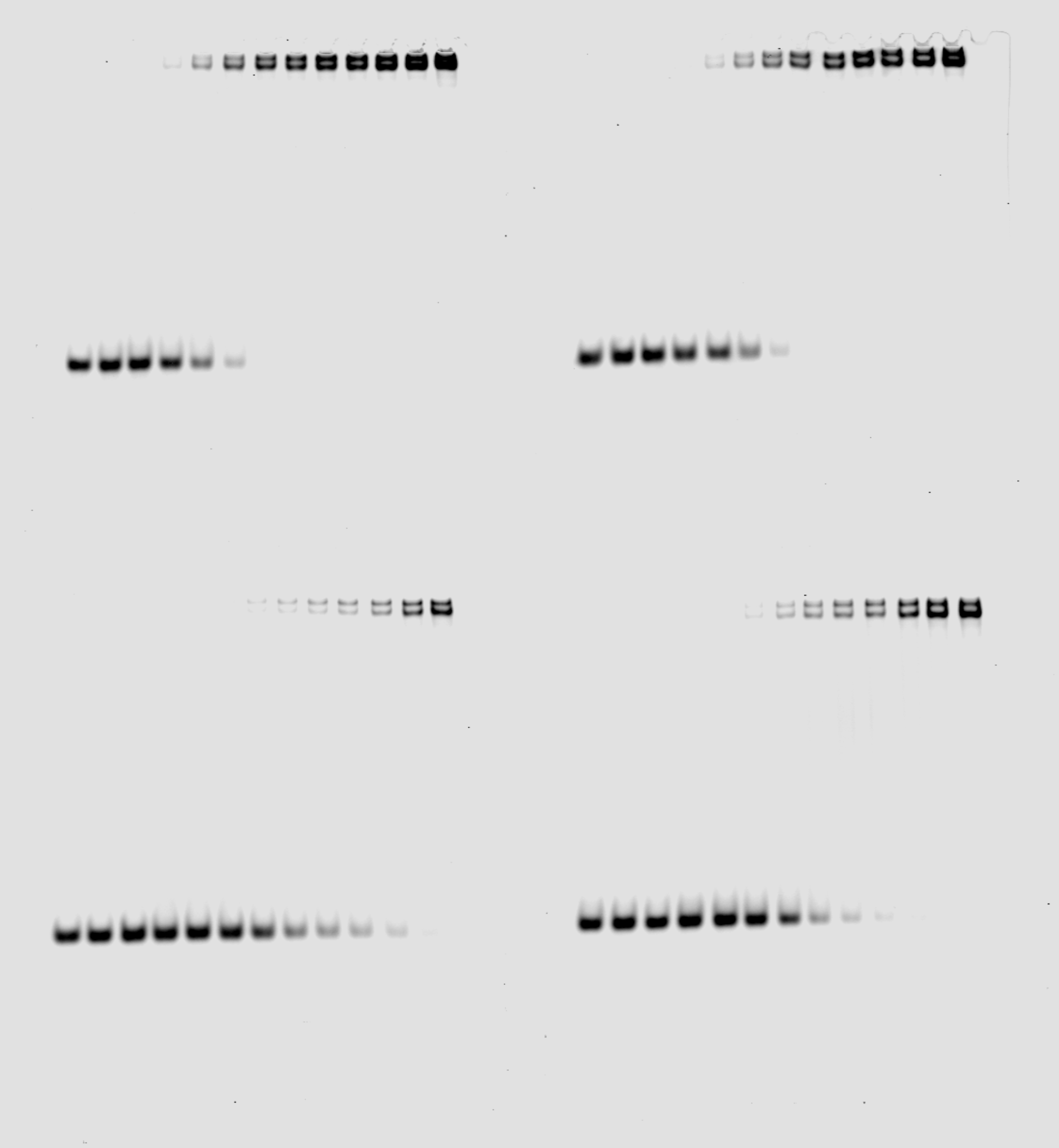

Supplement: Figure 1—source data 1. [file elife-85878-fig1-data1.zip › Figure 1b-source data 1.tif]

Figure 1b.

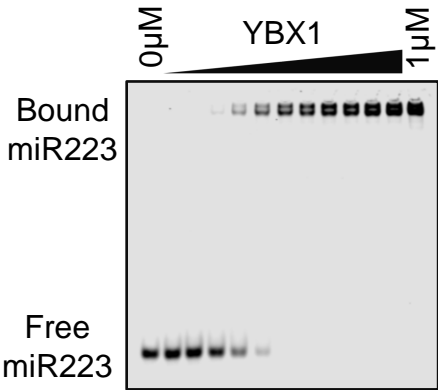

b1

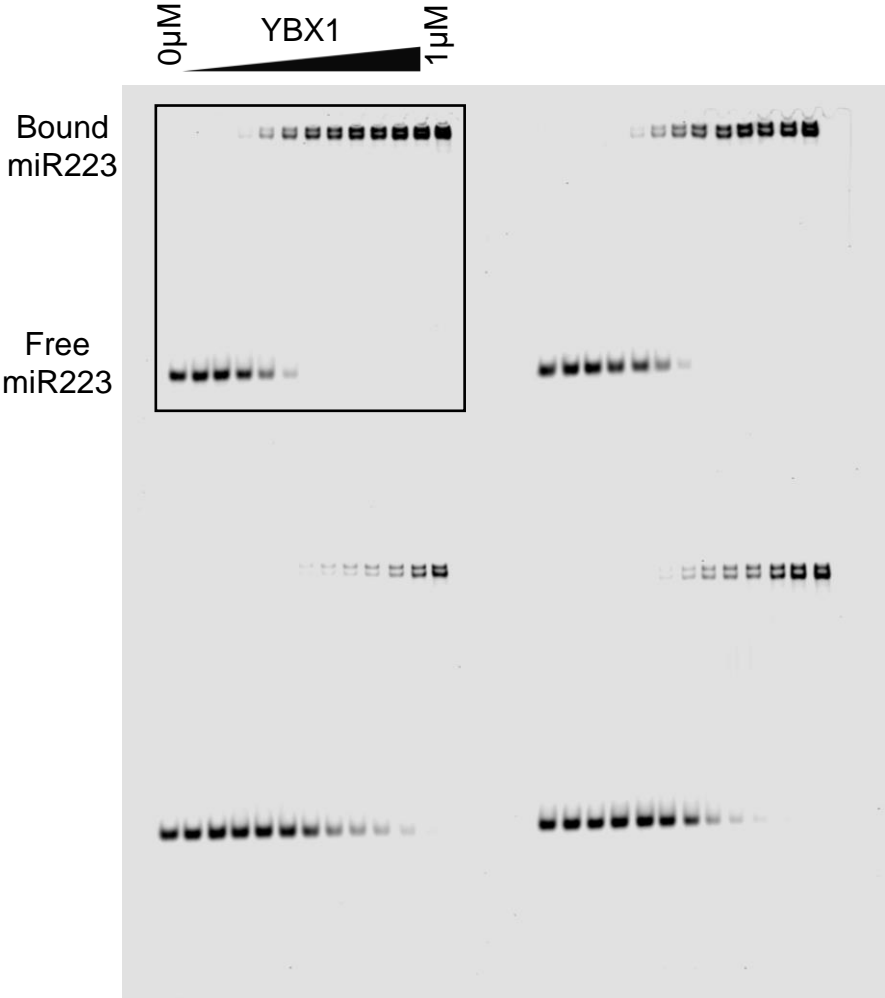

Supplement: Figure 1—source data 1. [file elife-85878-fig1-data1.zip › Figure 1b-Source data 2.pdf]

Figure 1c.

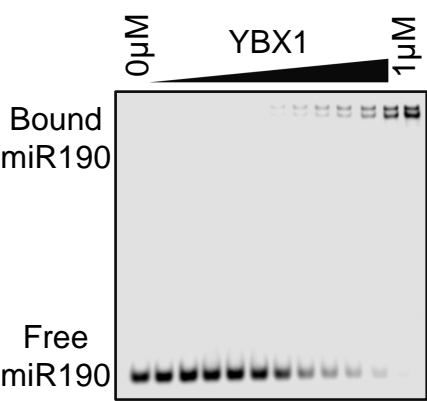

c1

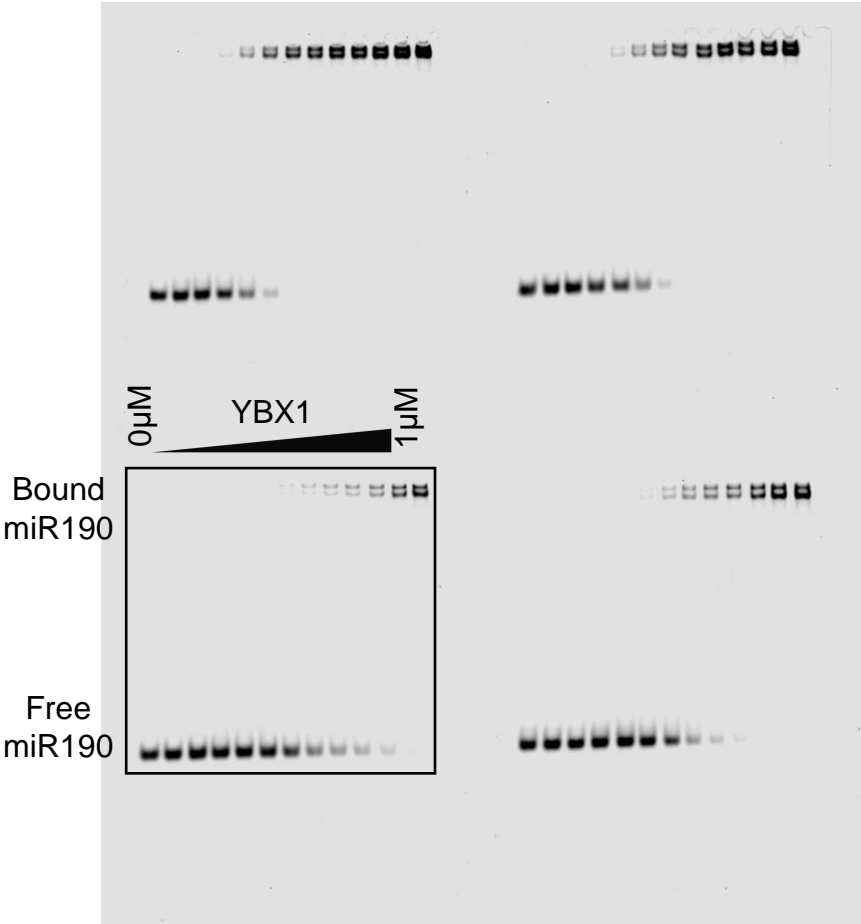

Supplement: Figure 1—source data 1. [file elife-85878-fig1-data1.zip › Figure 1c-Source data 2.pdf]

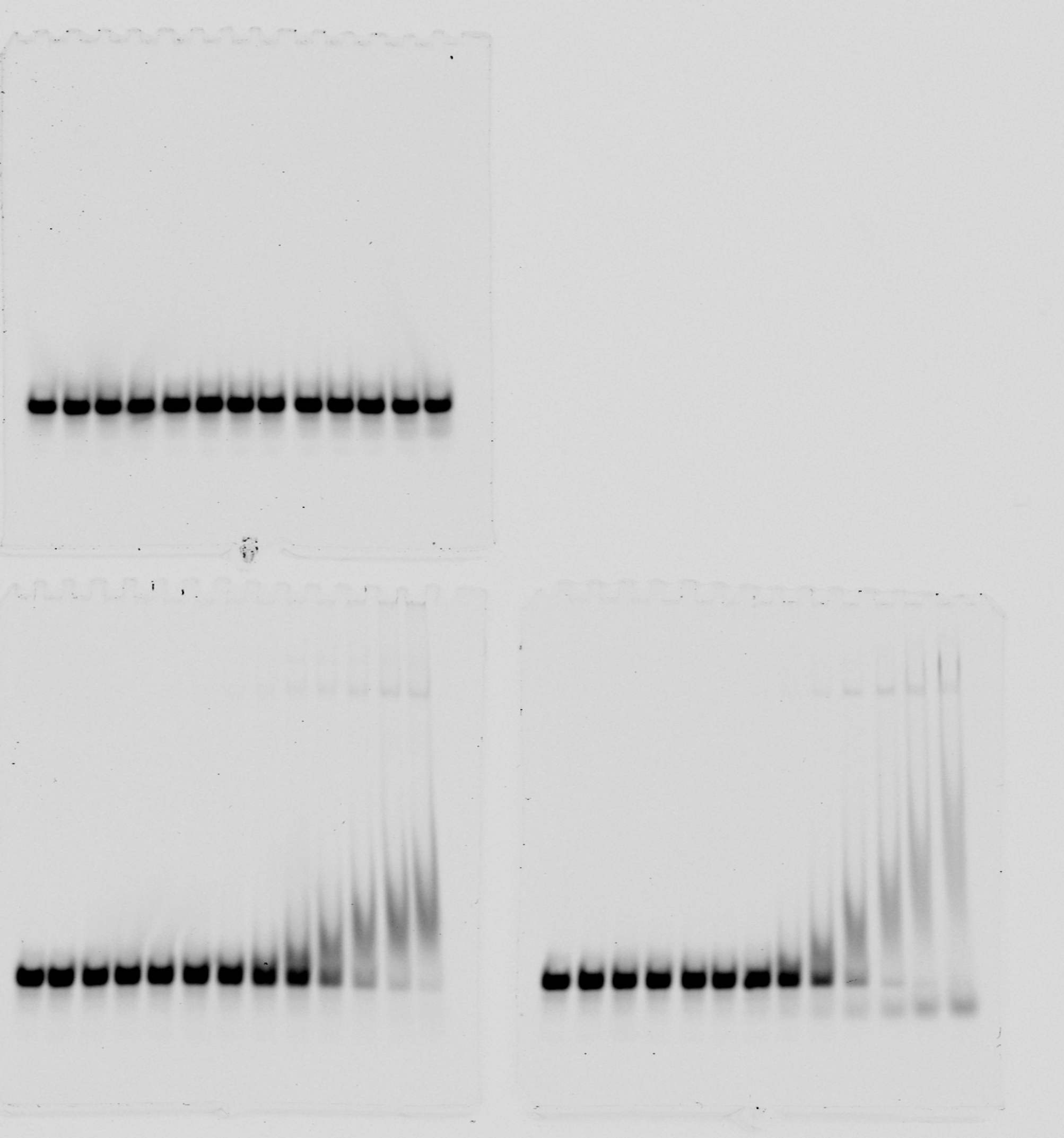

Supplement: Figure 1—source data 1. [file elife-85878-fig1-data1.zip › Figure 1f-source data 1.tif]

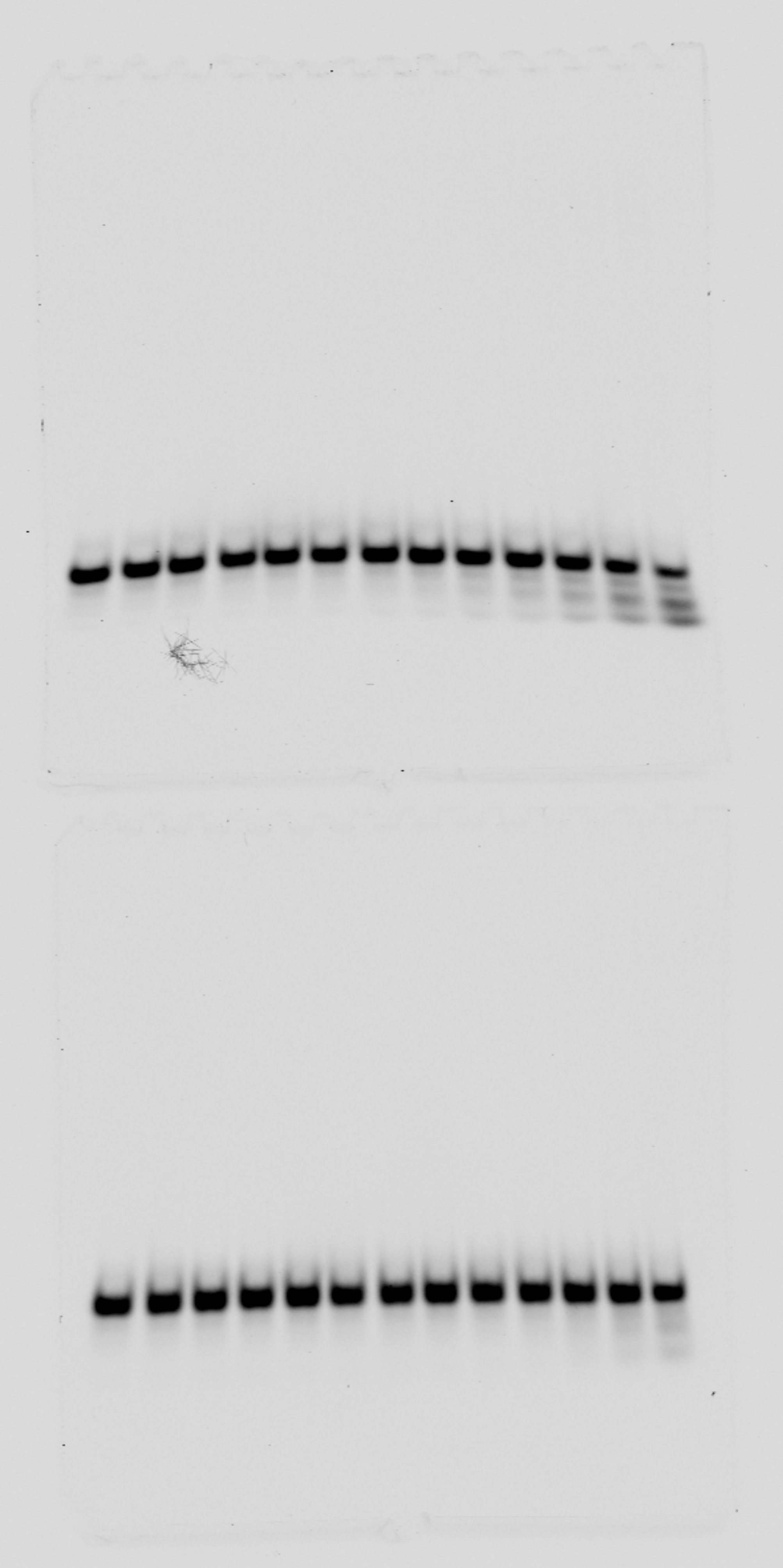

Supplement: Figure 1—source data 1. [file elife-85878-fig1-data1.zip › Figure 1f-source data 2.tif]

Figure 1f.

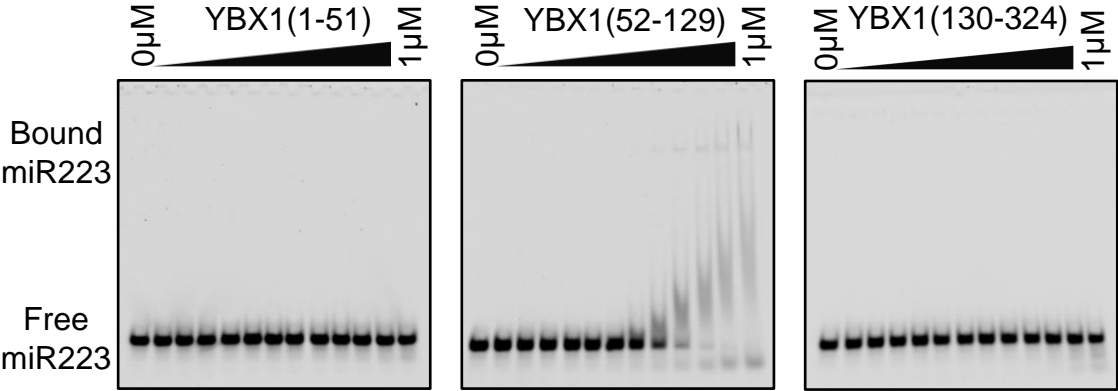

f1

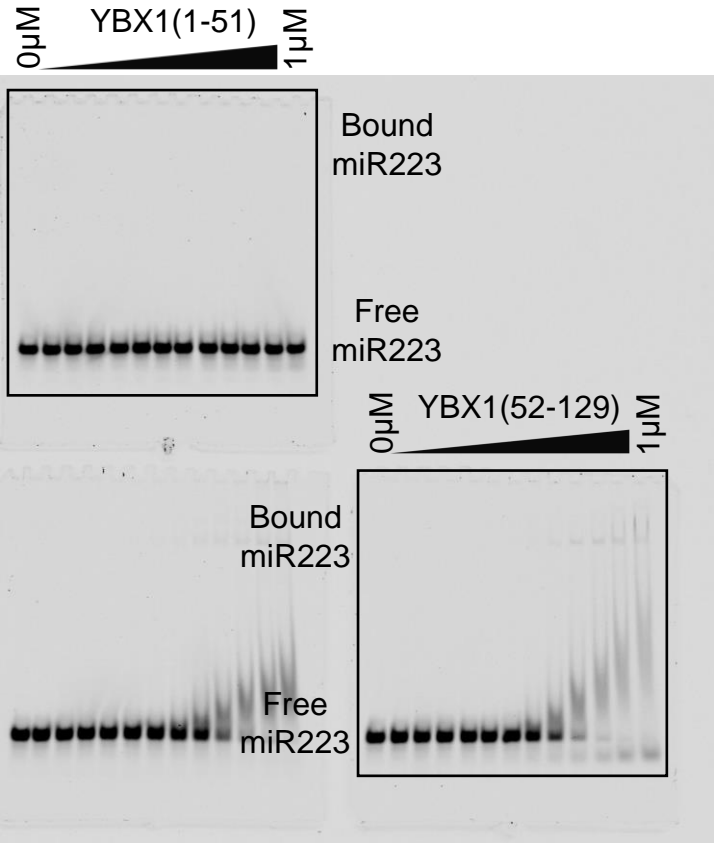

f2

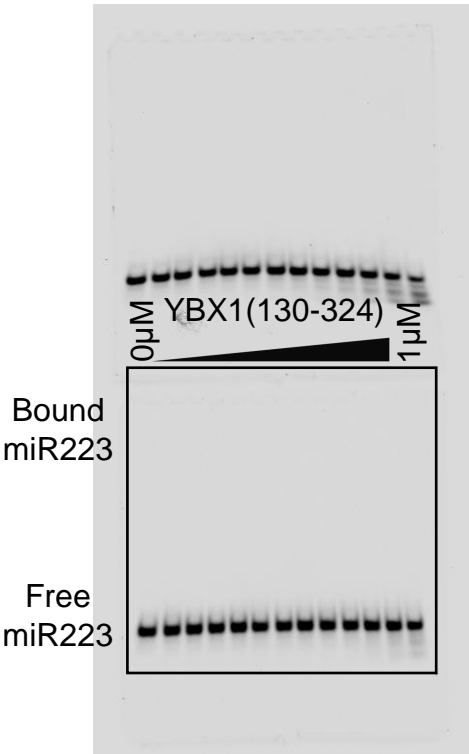

Supplement: Figure 1—source data 1. [file elife-85878-fig1-data1.zip › Figure 1f-source data 3.pdf]

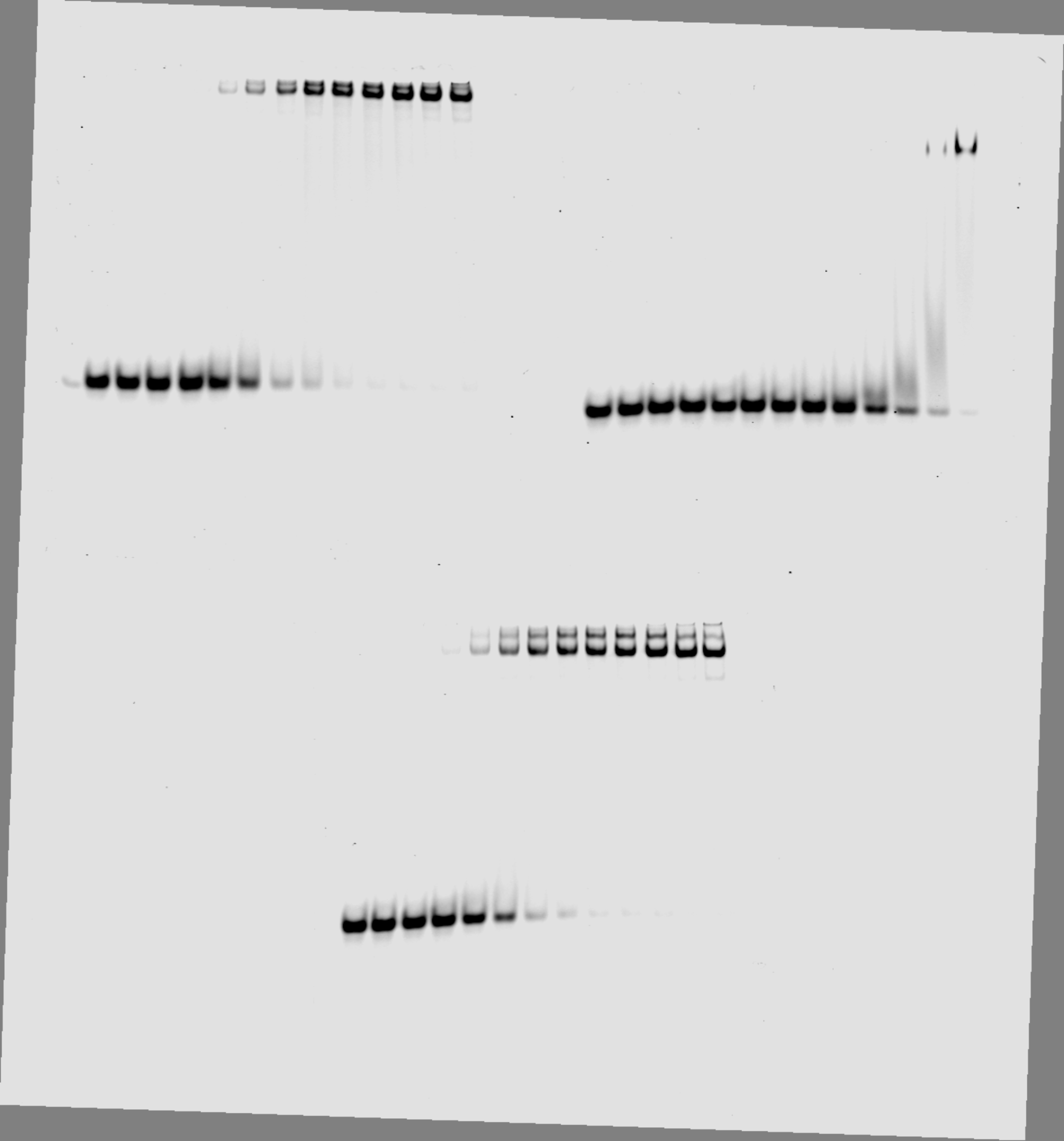

Supplement: Figure 1—source data 1. [file elife-85878-fig1-data1.zip › Figure 1g-source data 1.tif]

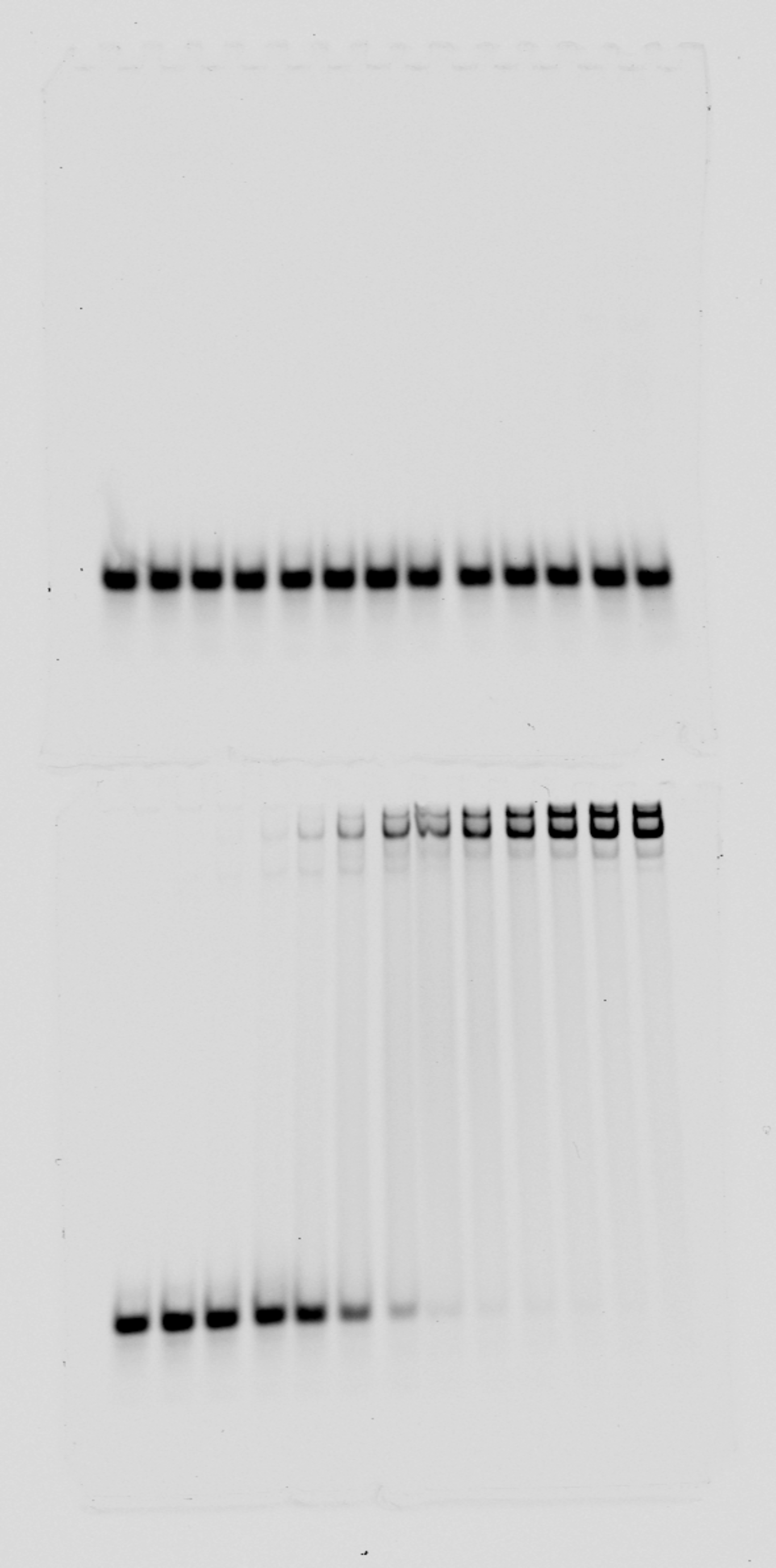

Supplement: Figure 1—source data 1. [file elife-85878-fig1-data1.zip › Figure 1g-source data 2.tif]

Figure 2g.

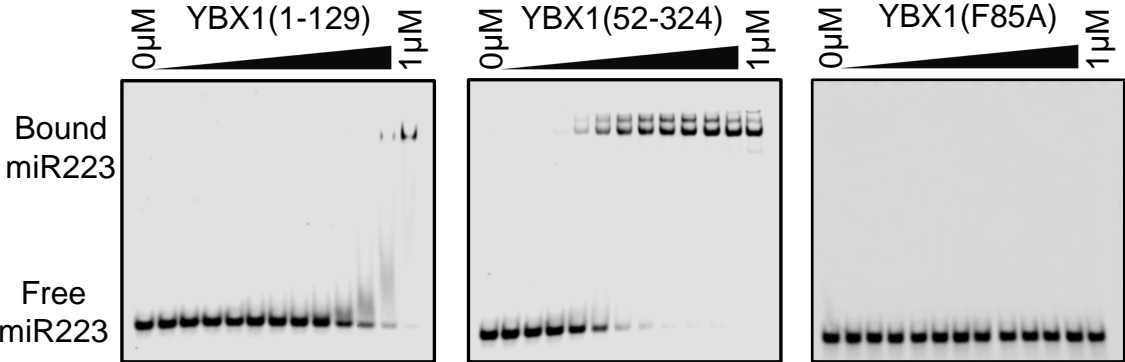

g1.

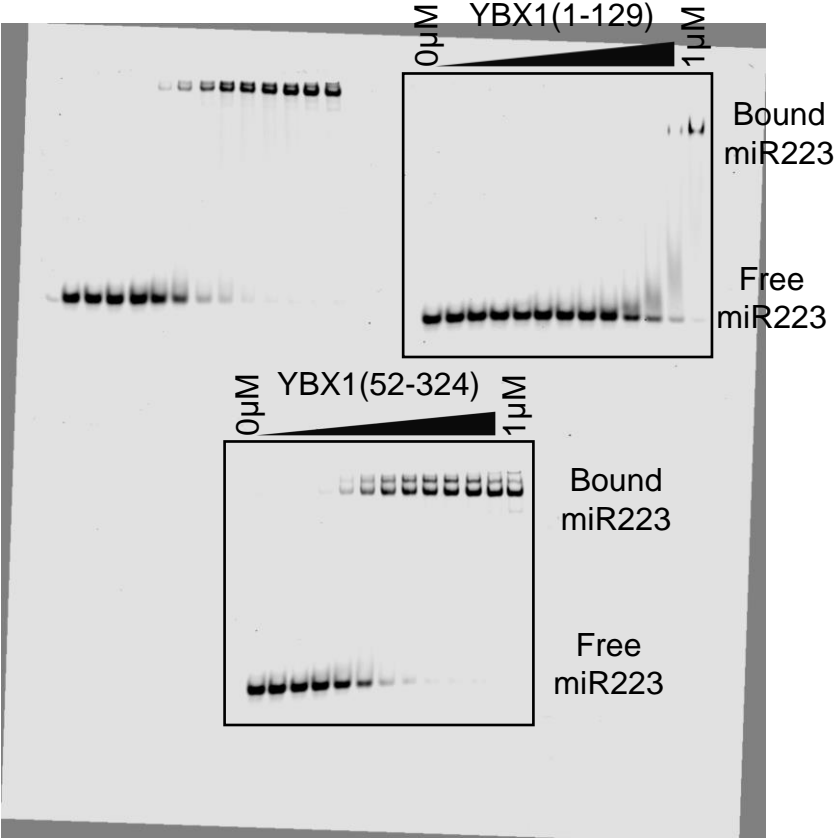

g2. 0  $\mu$ M YBX1(F85A) 1  $\mu$ M

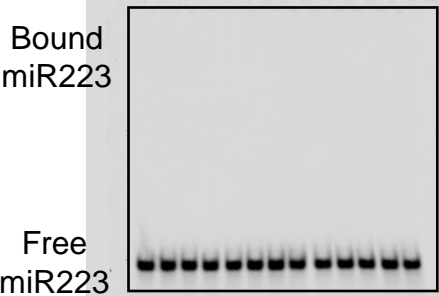

Supplement: Figure 1—source data 1. [file elife-85878-fig1-data1.zip › Figure 1g-source data 3.pdf]

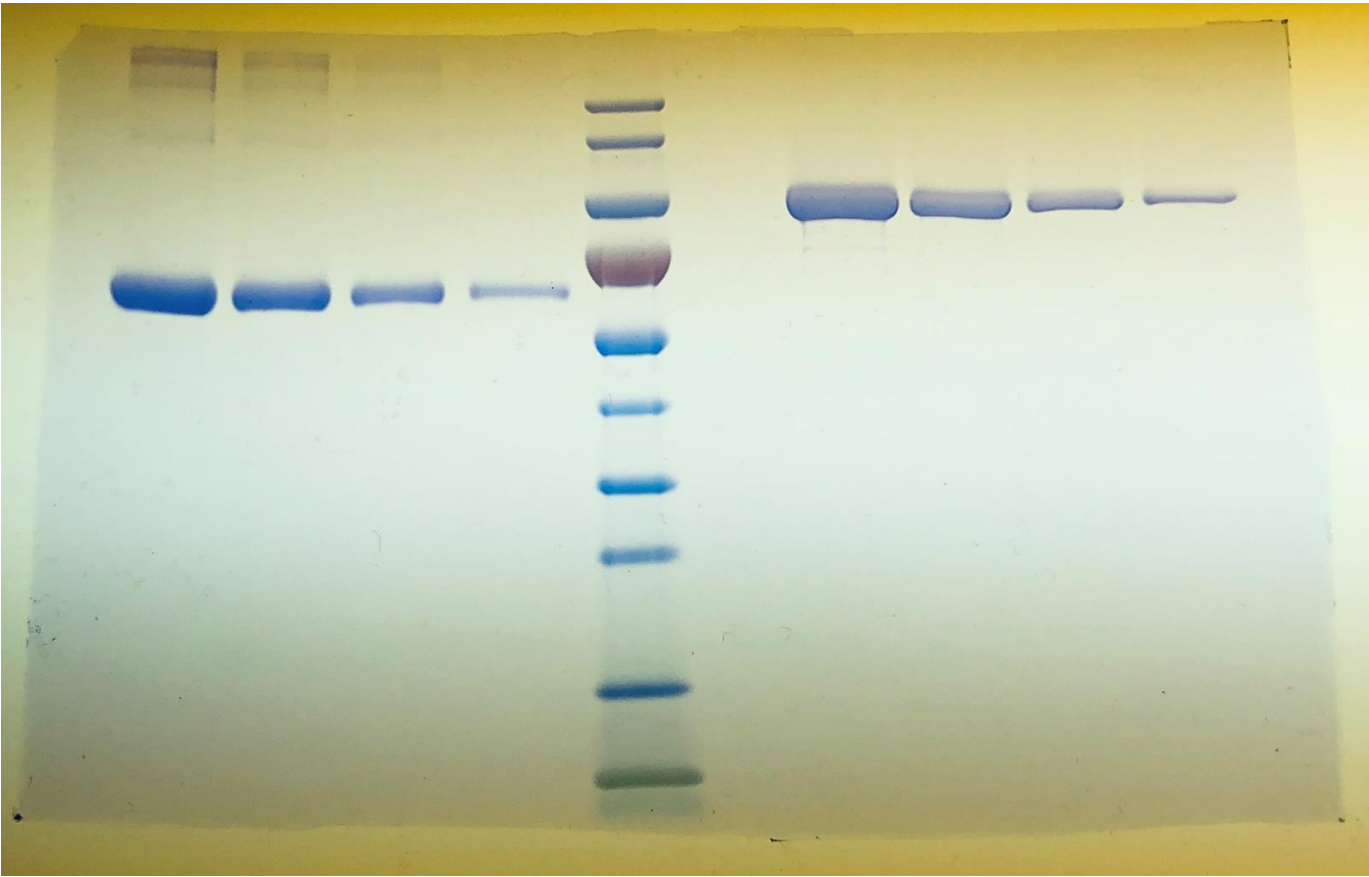

Supplement: Figure 1—figure supplement 2—source data 1. [file elife-85878-fig1-figsupp2-data1.zip › Figure 1-figure supplement 2a-source data 1.pdf]

Figure 1-figure supplement 2a.

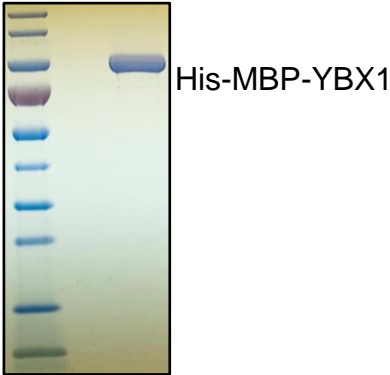

a1.

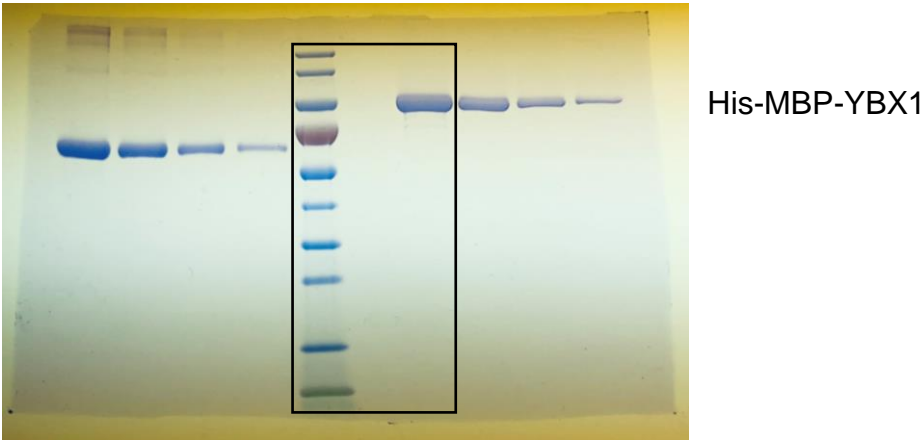

Supplement: Figure 1—figure supplement 2—source data 1. [file elife-85878-fig1-figsupp2-data1.zip › Figure 1-figure supplement 2a-source data 2.pdf]

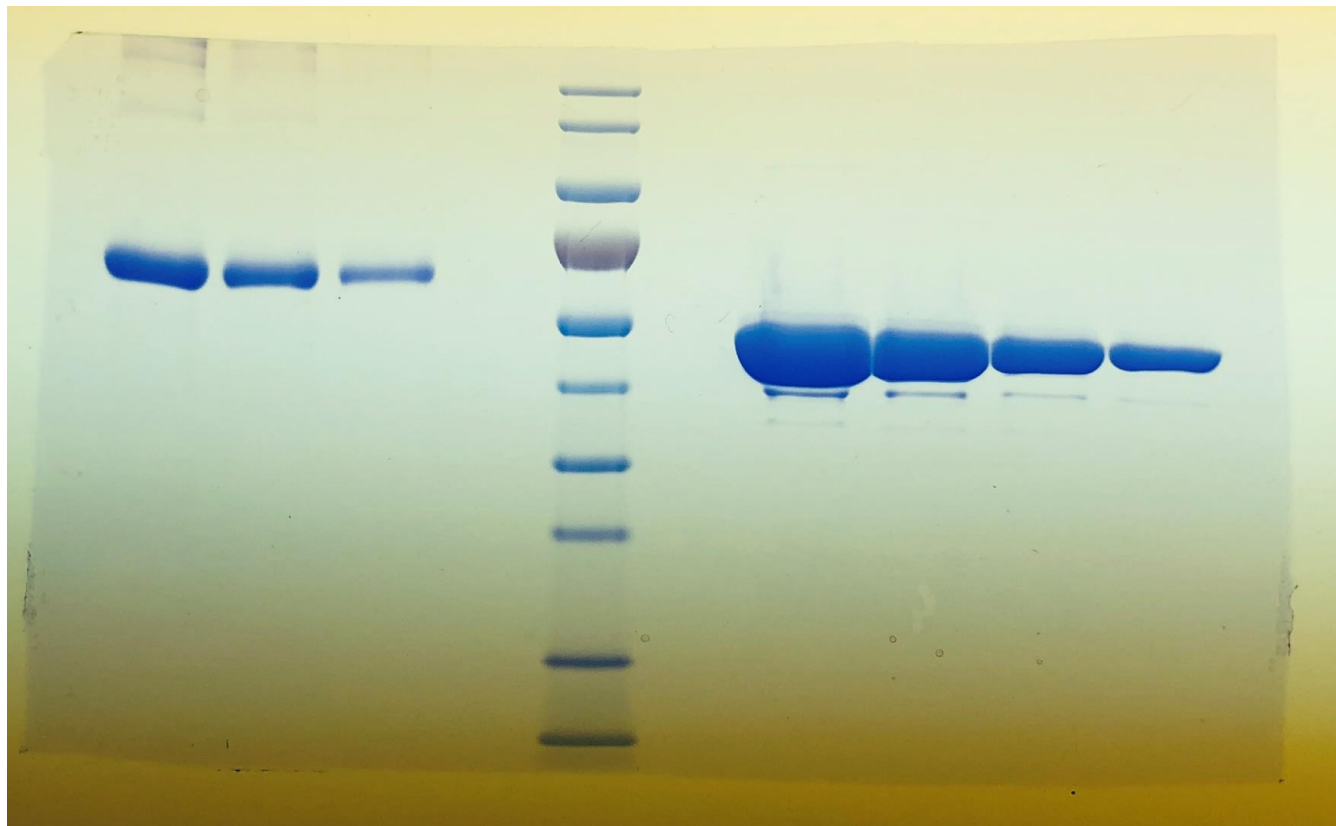

Supplement: Figure 1—figure supplement 2—source data 1. [file elife-85878-fig1-figsupp2-data1.zip › Figure 1-figure supplement 2b-source data 1.pdf]

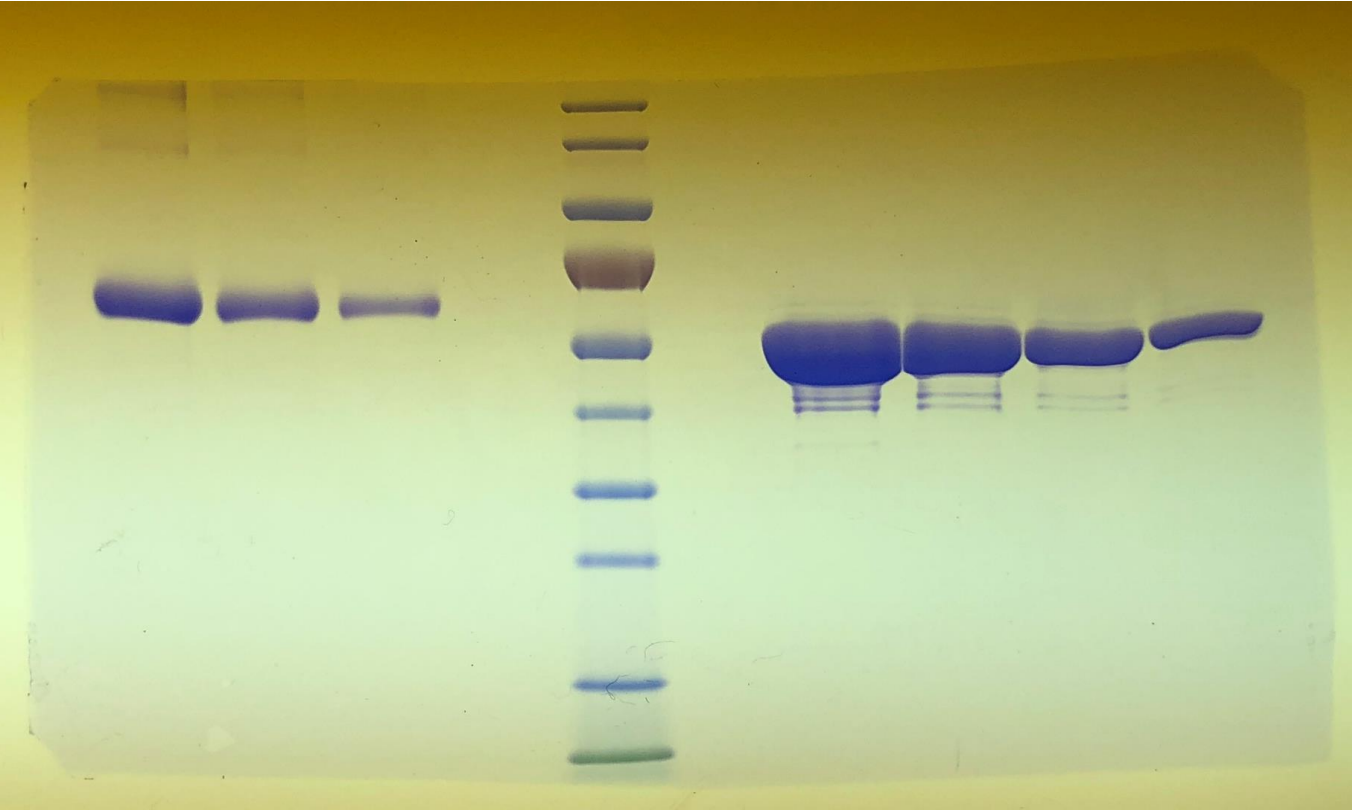

Supplement: Figure 1—figure supplement 2—source data 1. [file elife-85878-fig1-figsupp2-data1.zip › Figure 1-figure supplement 2b-source data 2.pdf]

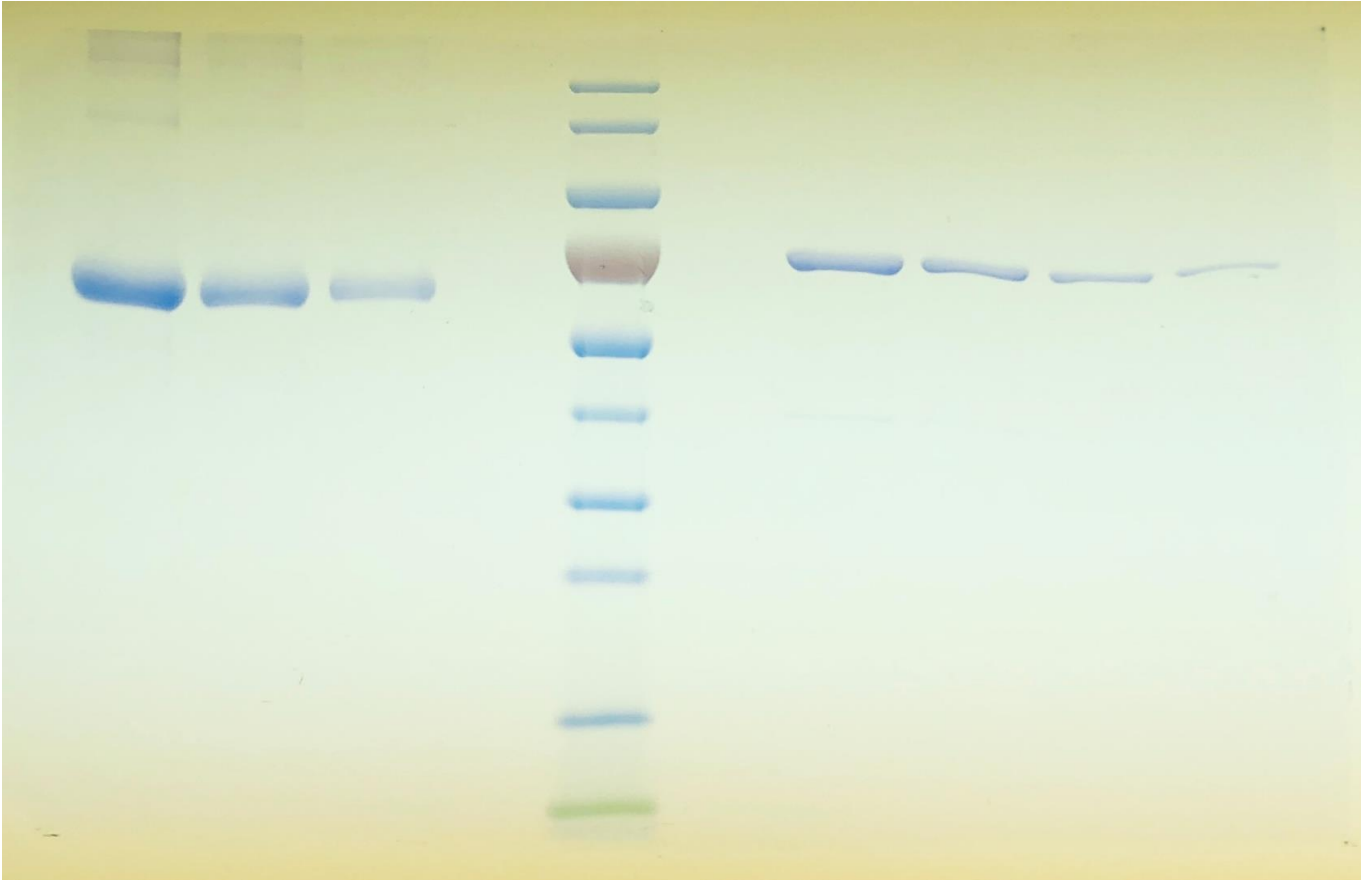

Supplement: Figure 1—figure supplement 2—source data 1. [file elife-85878-fig1-figsupp2-data1.zip › Figure 1-figure supplement 2b-source data 3.pdf]

Figure 1-figure supplement 2b.

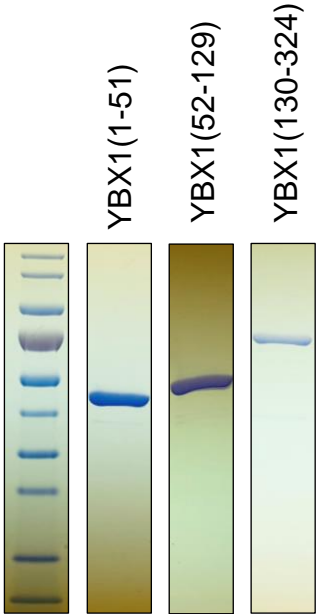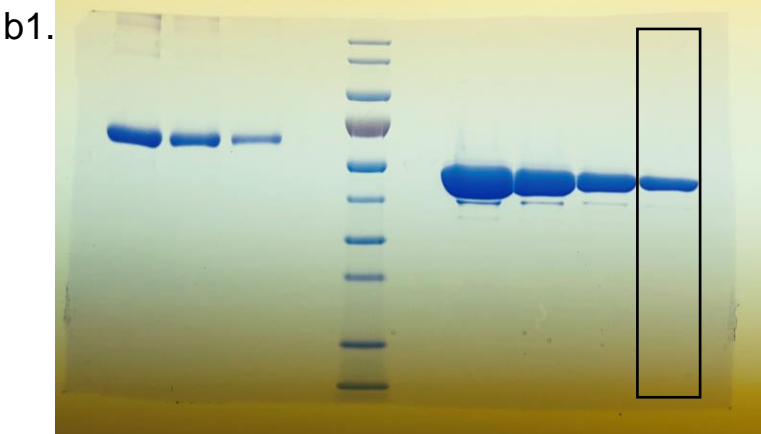

YBX1(1-51)

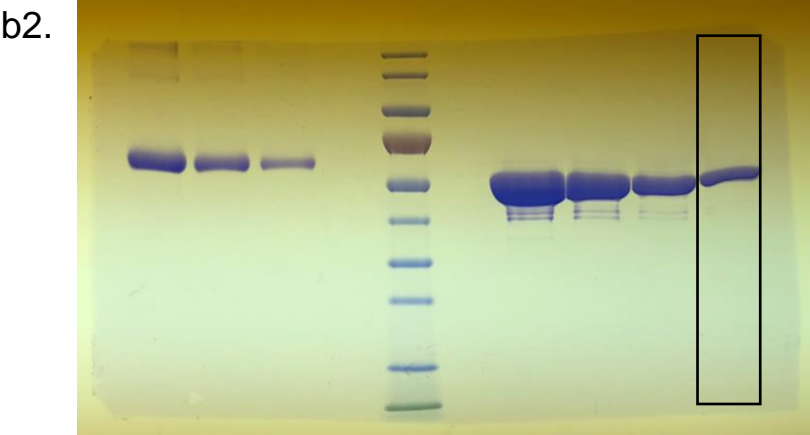

YBX1(52-129)

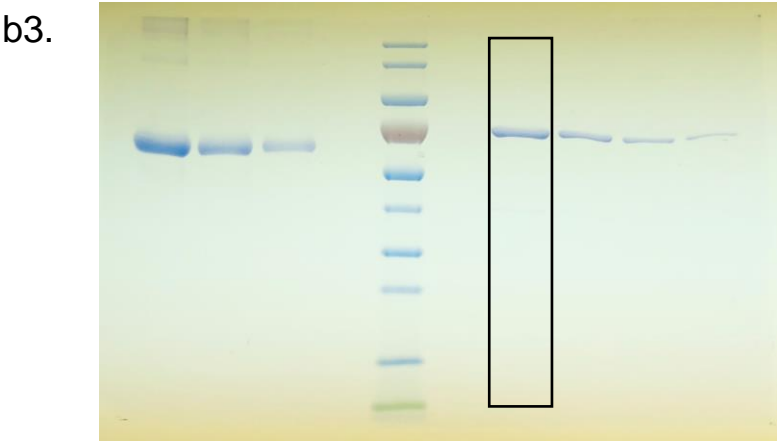

YBX1(130-324)

Supplement: Figure 1—figure supplement 2—source data 1. [file elife-85878-fig1-figsupp2-data1.zip › Figure 1-figure supplement 2b-source data 4.pdf]

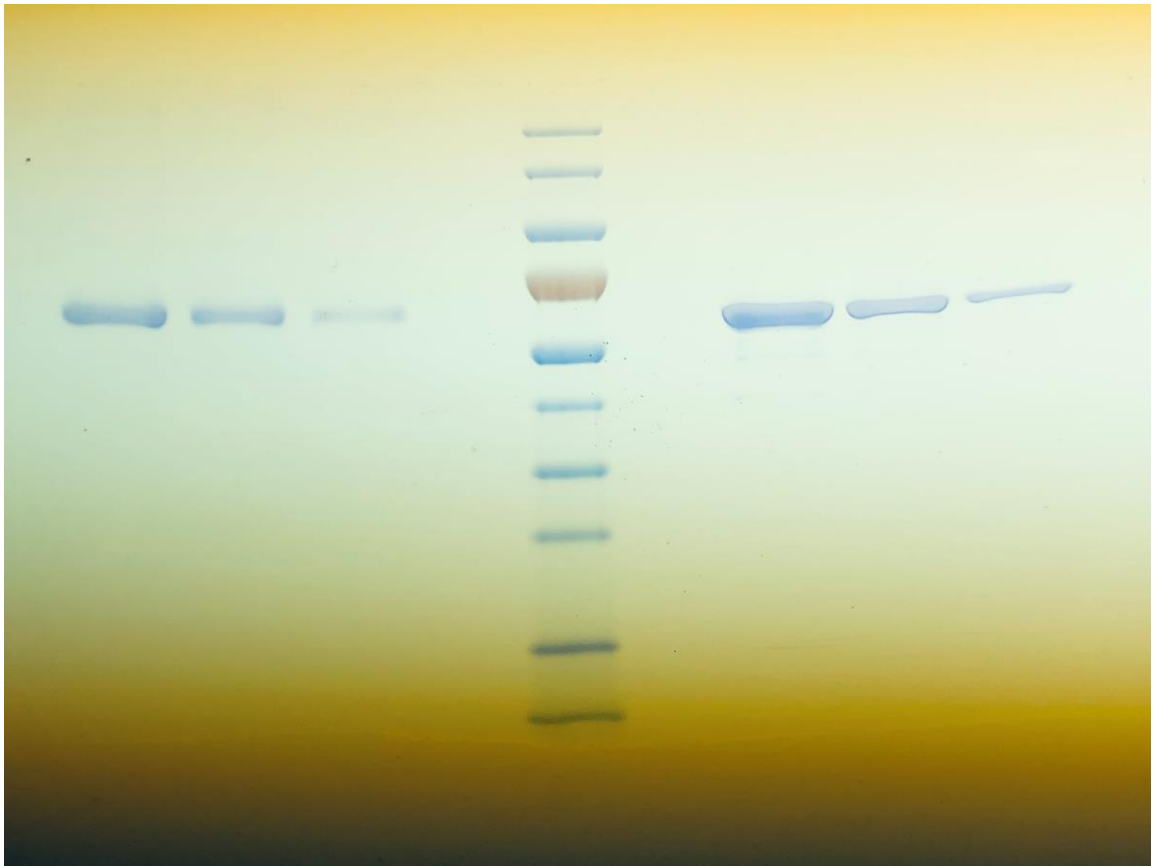

Supplement: Figure 1—figure supplement 2—source data 1. [file elife-85878-fig1-figsupp2-data1.zip › Figure 1-figure supplement 2c-source data 1.pdf]

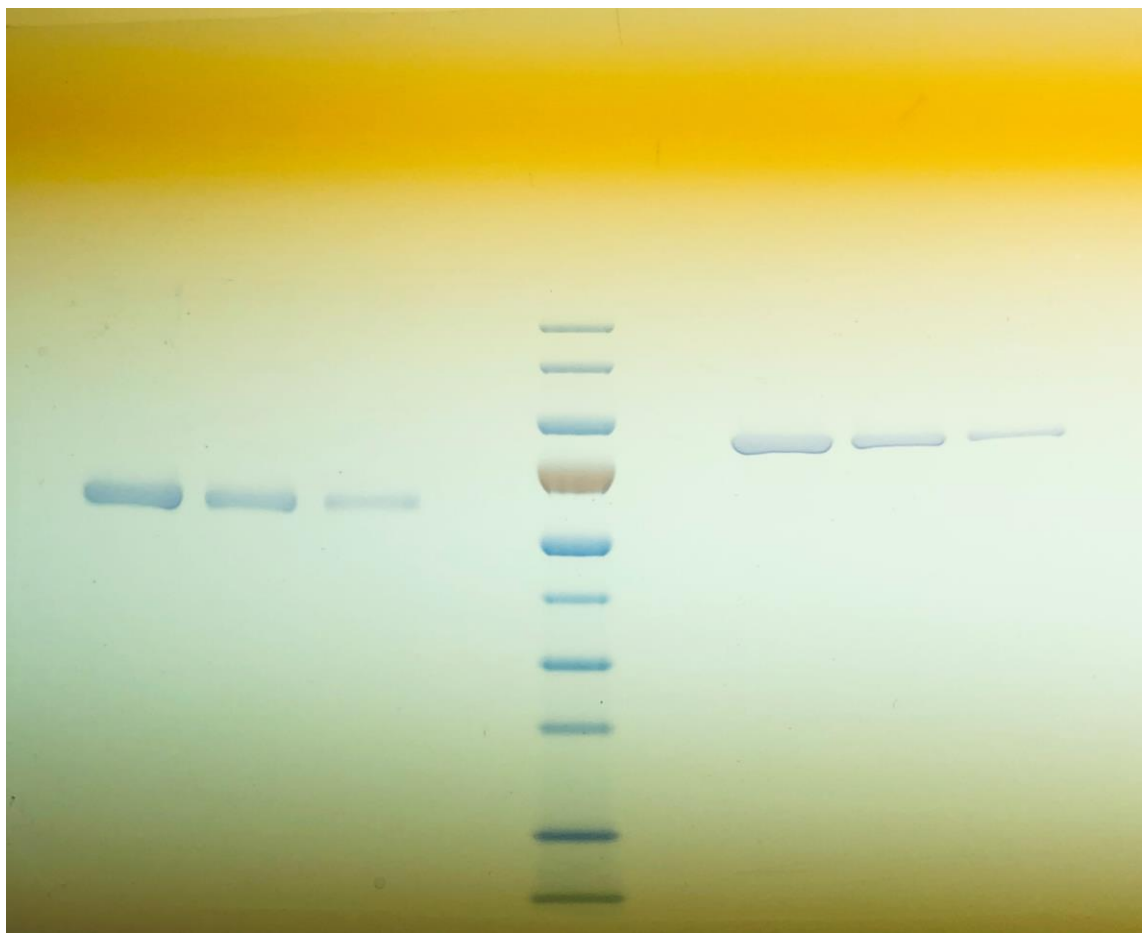

Supplement: Figure 1—figure supplement 2—source data 1. [file elife-85878-fig1-figsupp2-data1.zip › Figure 1-figure supplement 2c-source data 2.pdf]

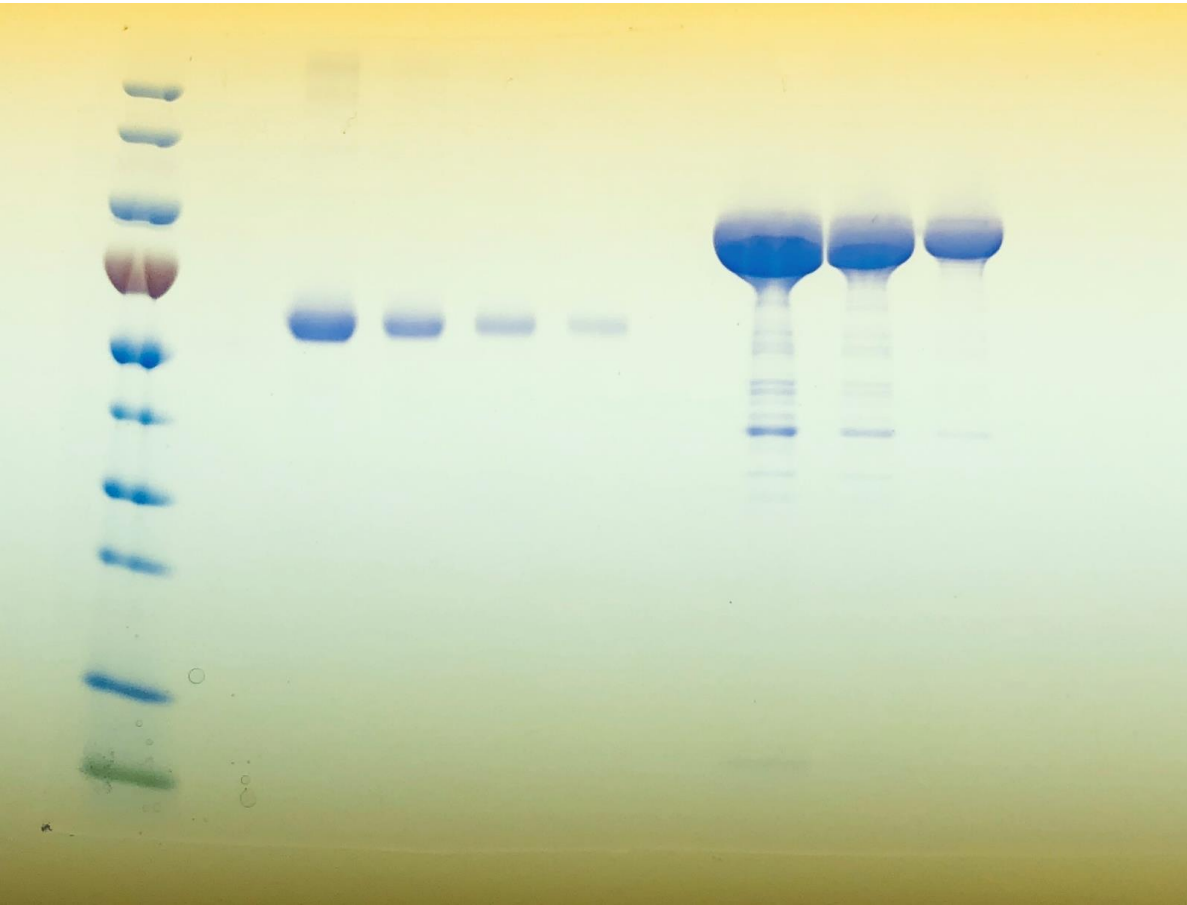

Supplement: Figure 1—figure supplement 2—source data 1. [file elife-85878-fig1-figsupp2-data1.zip › Figure 1-figure supplement 2c-source data 3.pdf]

Figure 1-figure supplement 2c.

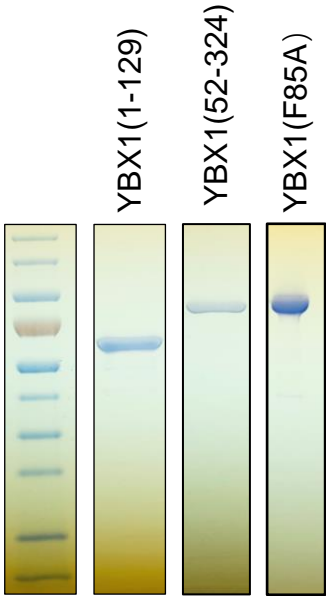

c1.

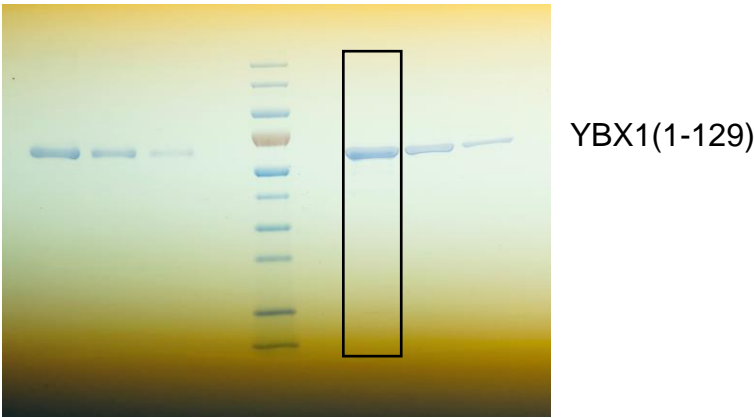

c2.

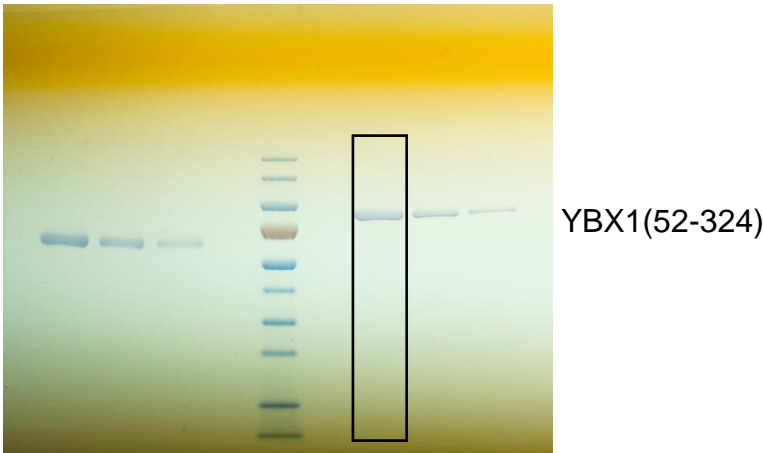

c3.

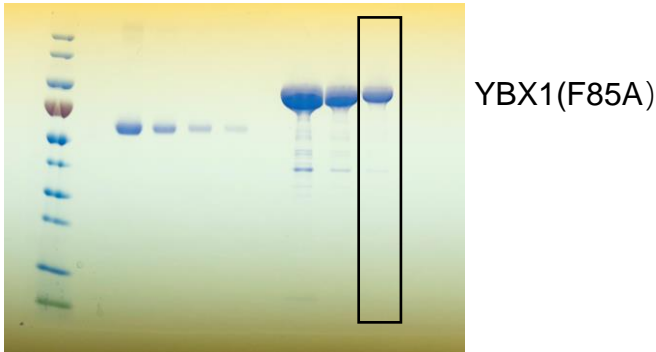

Supplement: Figure 1—figure supplement 2—source data 1. [file elife-85878-fig1-figsupp2-data1.zip › Figure 1-figure supplement 2c-source data 4.pdf]

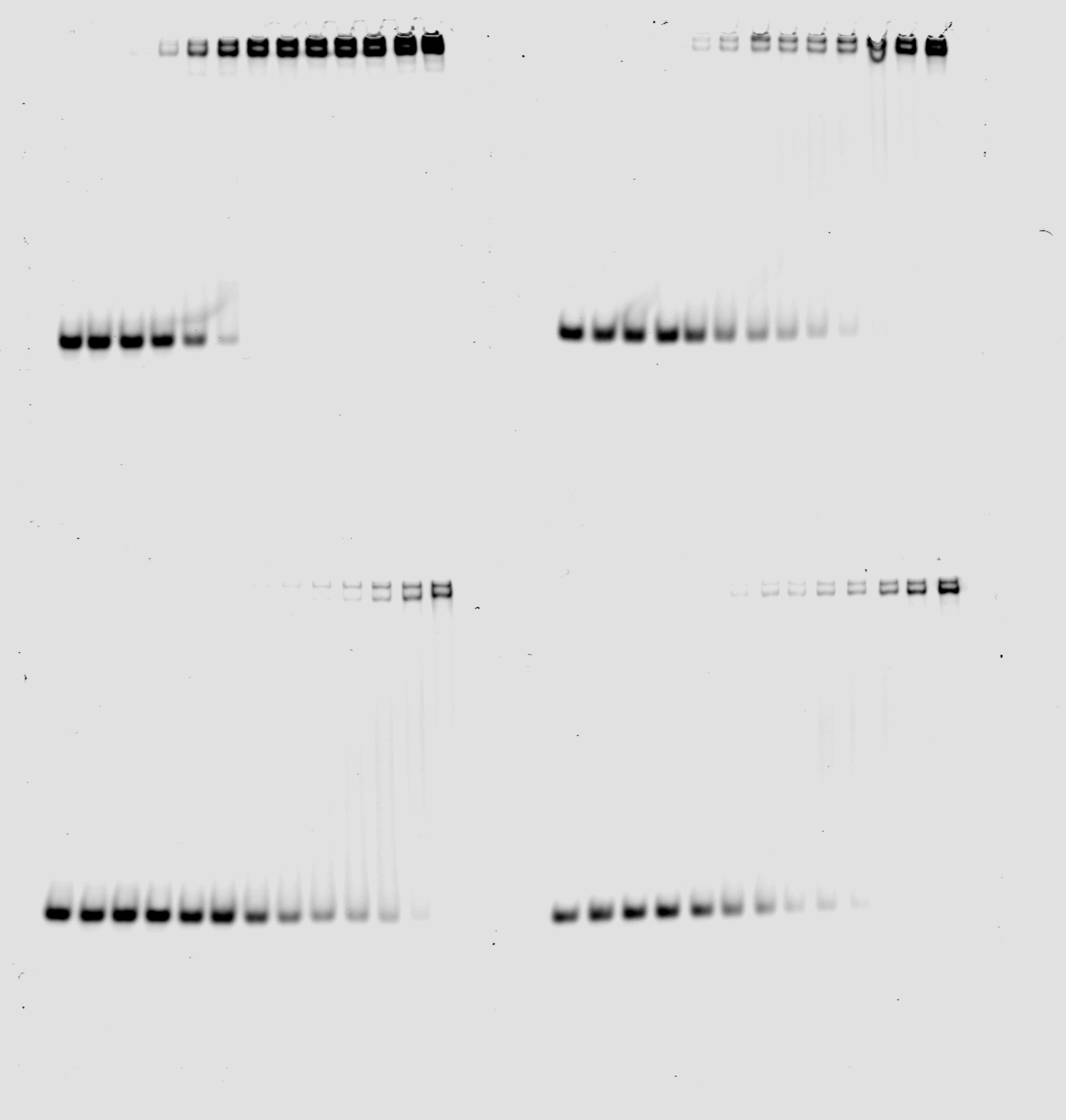

Supplement: Figure 2—source data 1. [file elife-85878-fig2-data1.zip › Figure 2b-source data 1.tif]

Figure 2b.

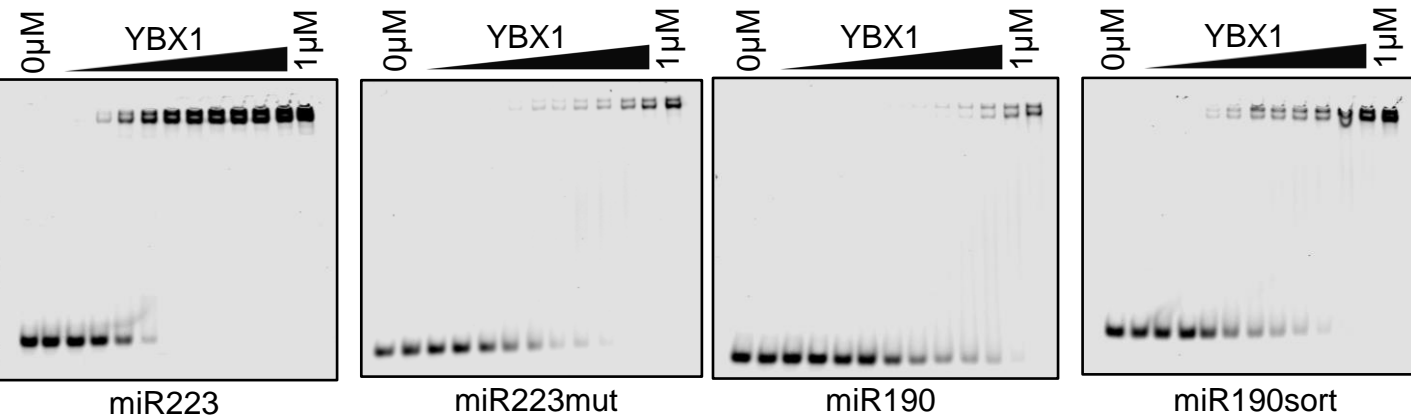

b1.

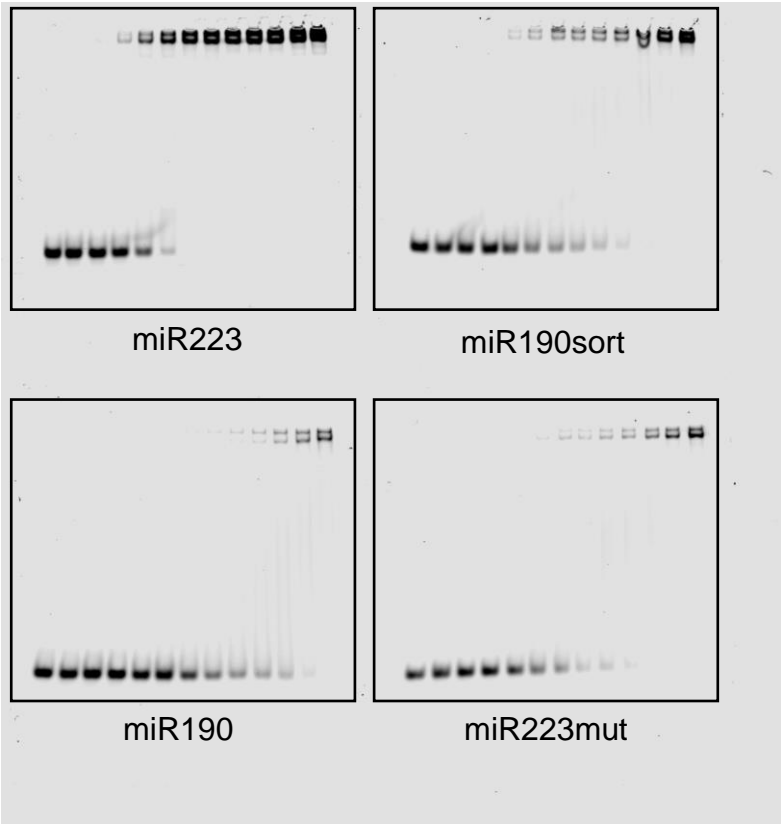

Supplement: Figure 2—source data 1. [file elife-85878-fig2-data1.zip › Figure 2b-source data 2.pdf]

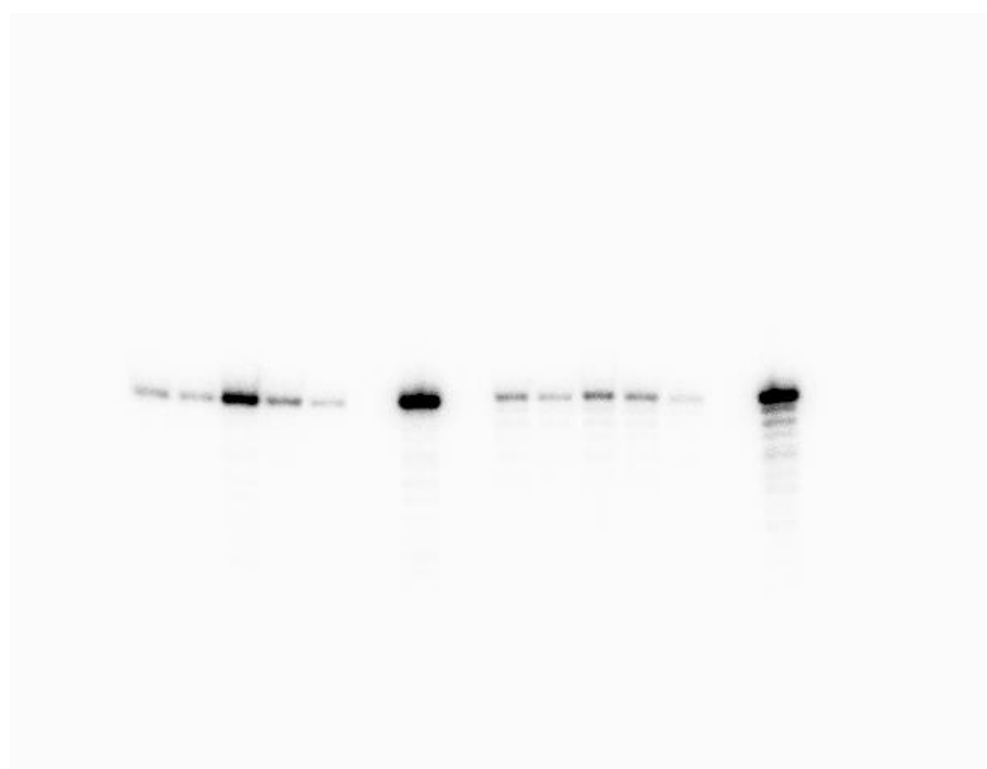

Supplement: Figure 2—source data 1. [file elife-85878-fig2-data1.zip › Figure 2g-source data 1.pdf]

Figure 2g.

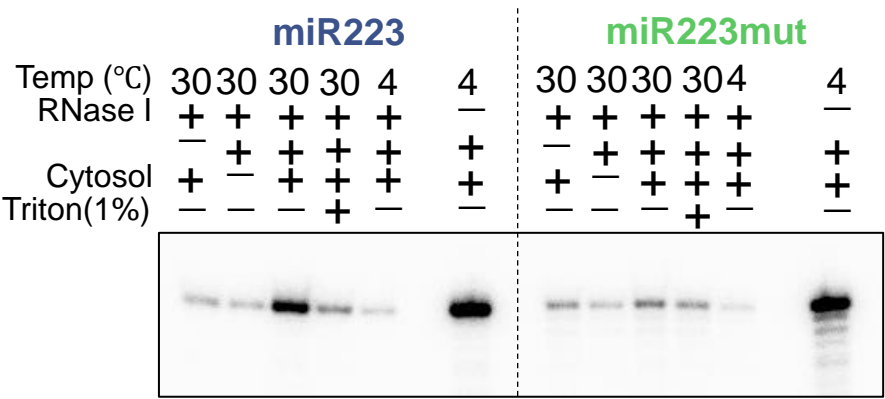

g1.

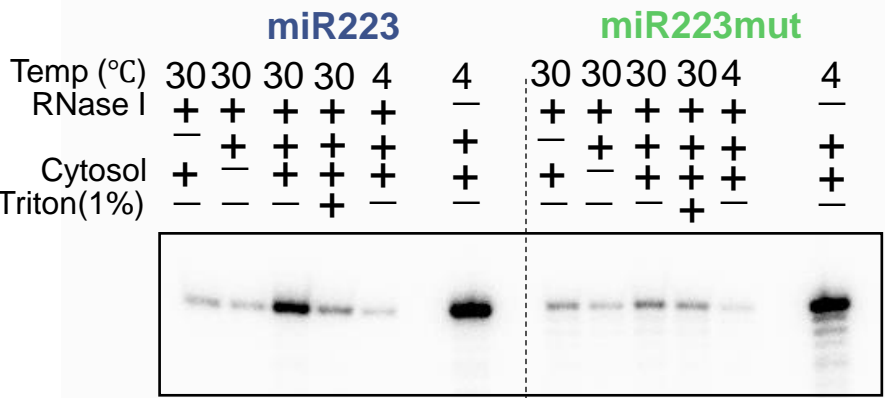

Supplement: Figure 2—source data 1. [file elife-85878-fig2-data1.zip › Figure 2g-source data 2.pdf]

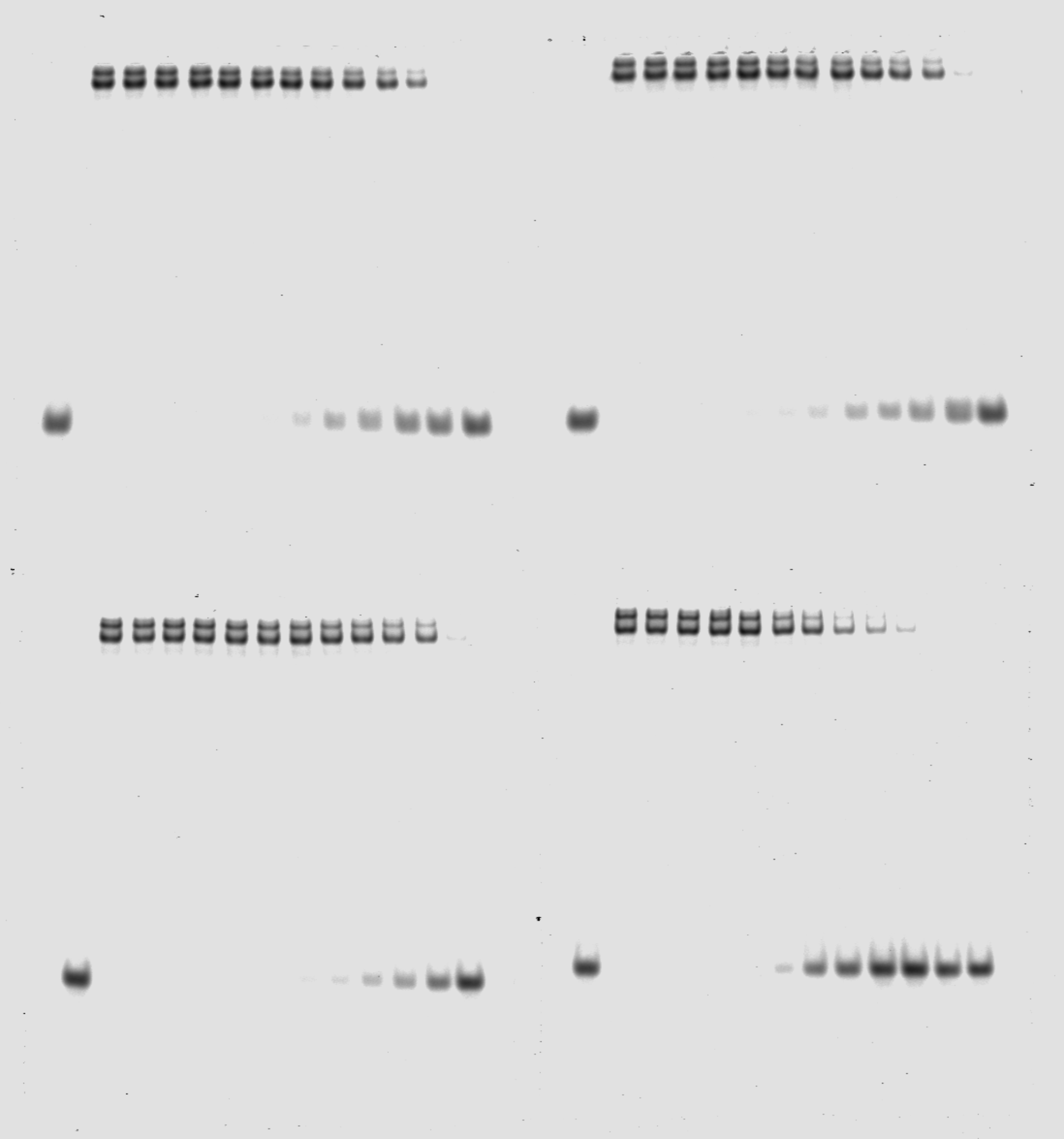

Supplement: Figure 2—figure supplement 1—source data 1. [file elife-85878-fig2-figsupp1-data1.zip › Figure 2-figure supplement 1a,1b,1e,1f-source data 1.tif]

Figure 2-figure supplement 1a

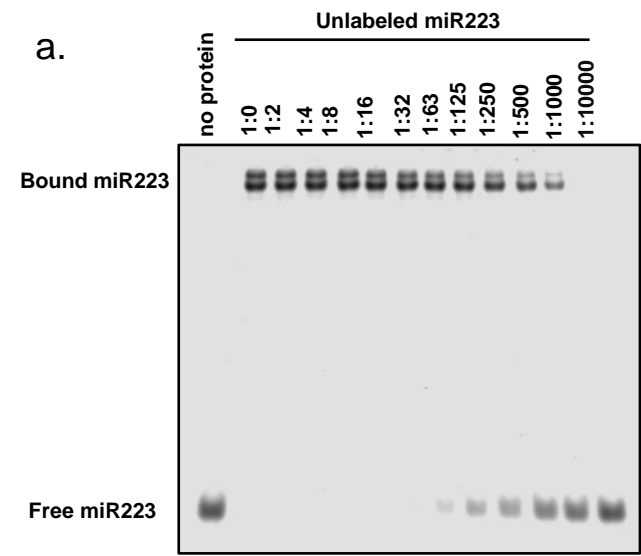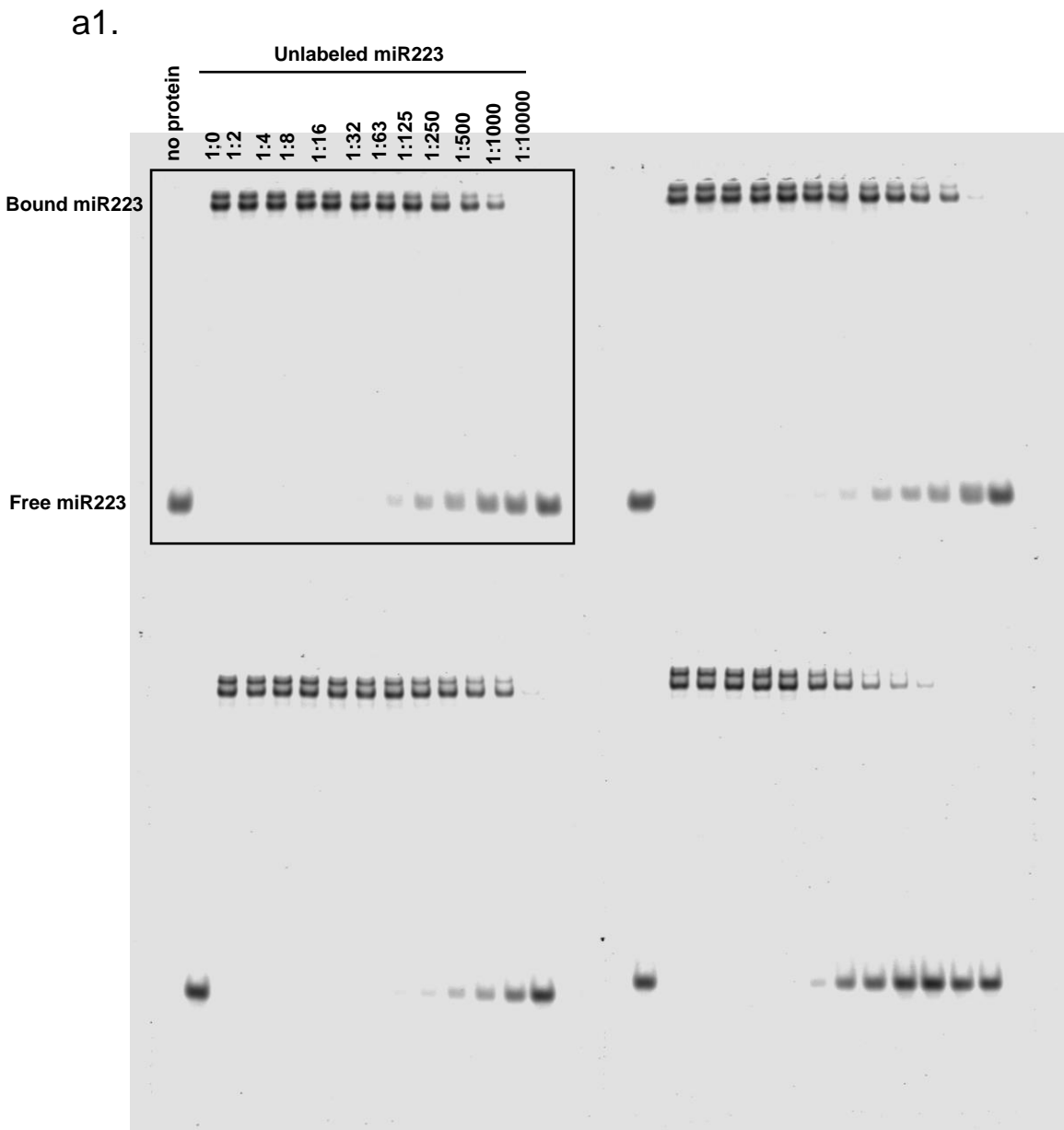

Supplement: Figure 2—figure supplement 1—source data 1. [file elife-85878-fig2-figsupp1-data1.zip › Figure 2-figure supplement 1a-source data 2.pdf]

Figure 2-figure supplement 1b

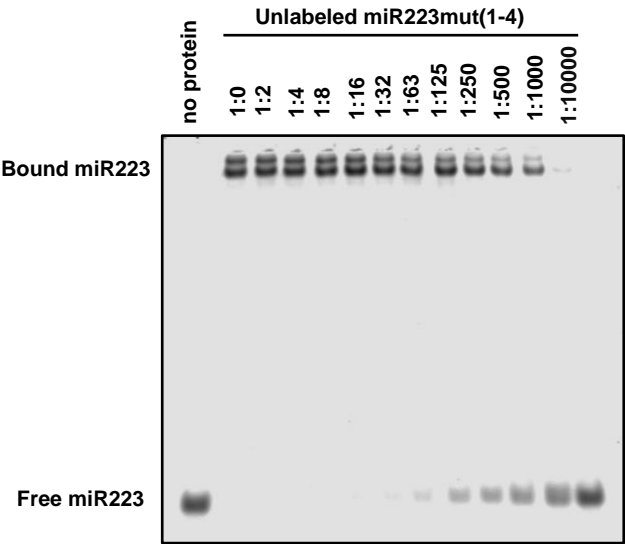

b1.

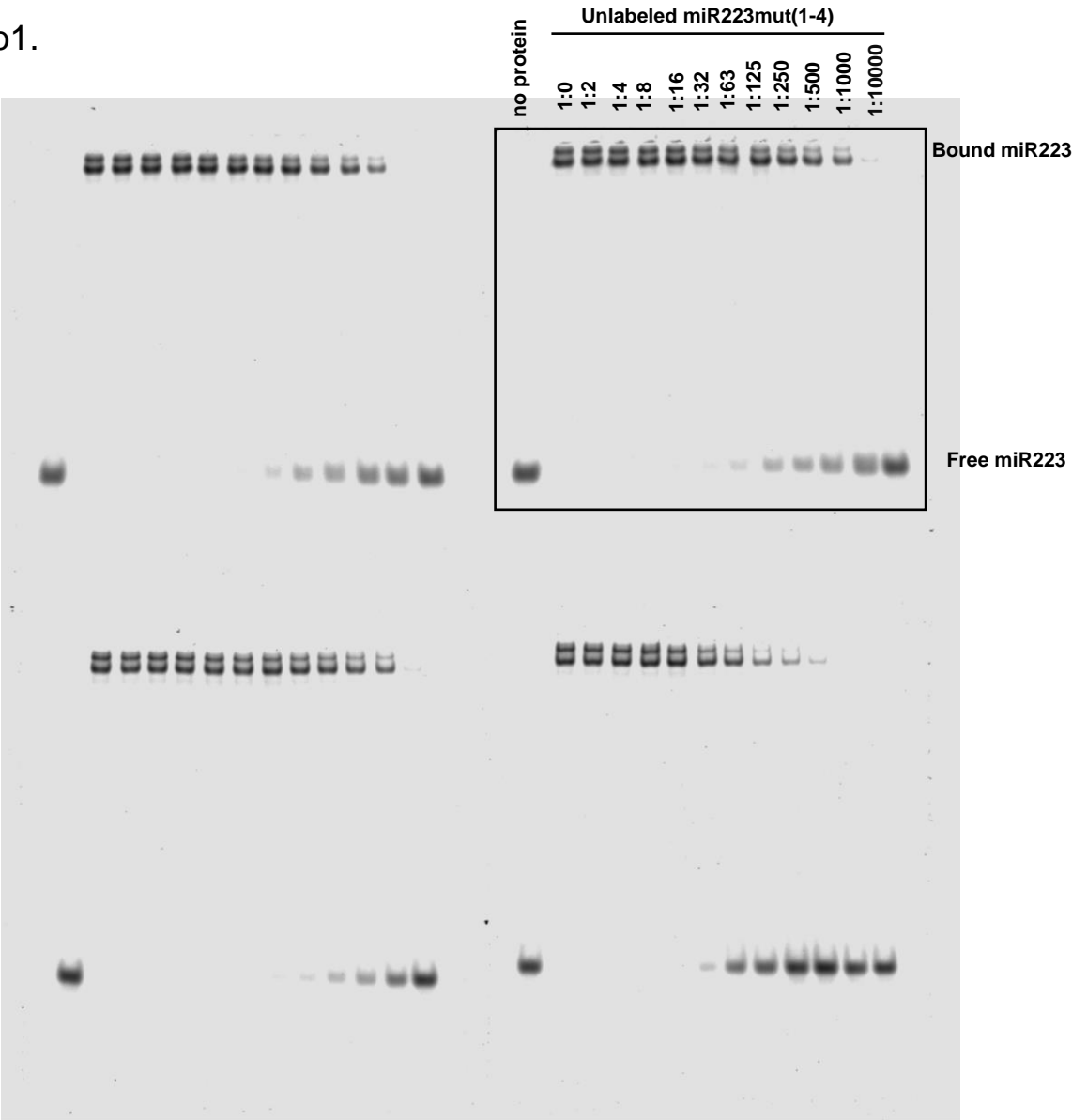

Supplement: Figure 2—figure supplement 1—source data 1. [file elife-85878-fig2-figsupp1-data1.zip › Figure 2-figure supplement 1b-source data 2.pdf]

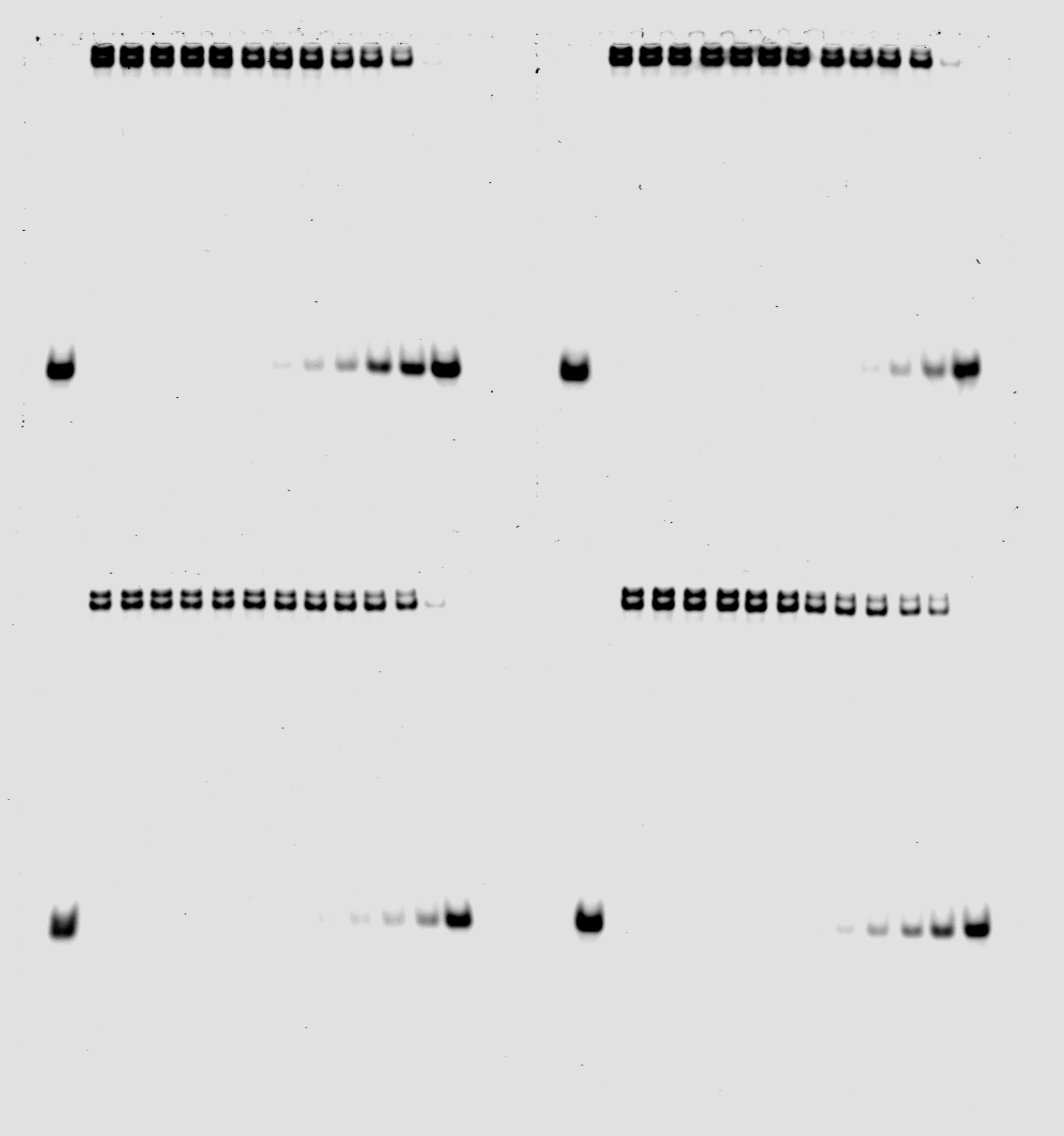

Supplement: Figure 2—figure supplement 1—source data 1. [file elife-85878-fig2-figsupp1-data1.zip › Figure 2-figure supplement 1c-source data 1.tif]

Figure 2-figure supplement 1c.

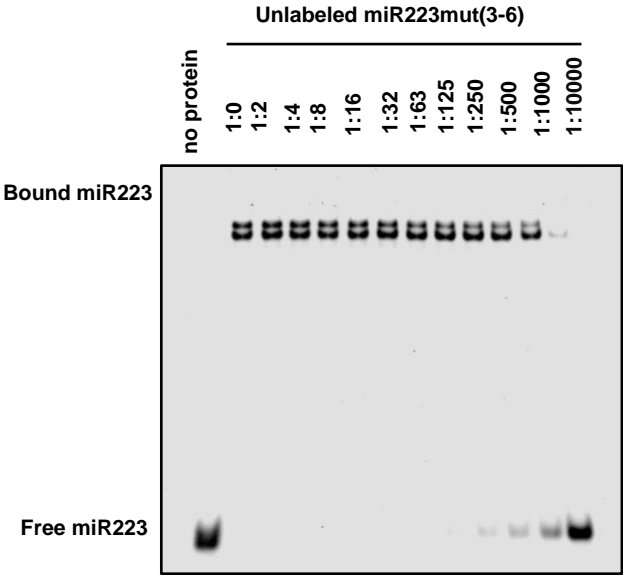

c1.

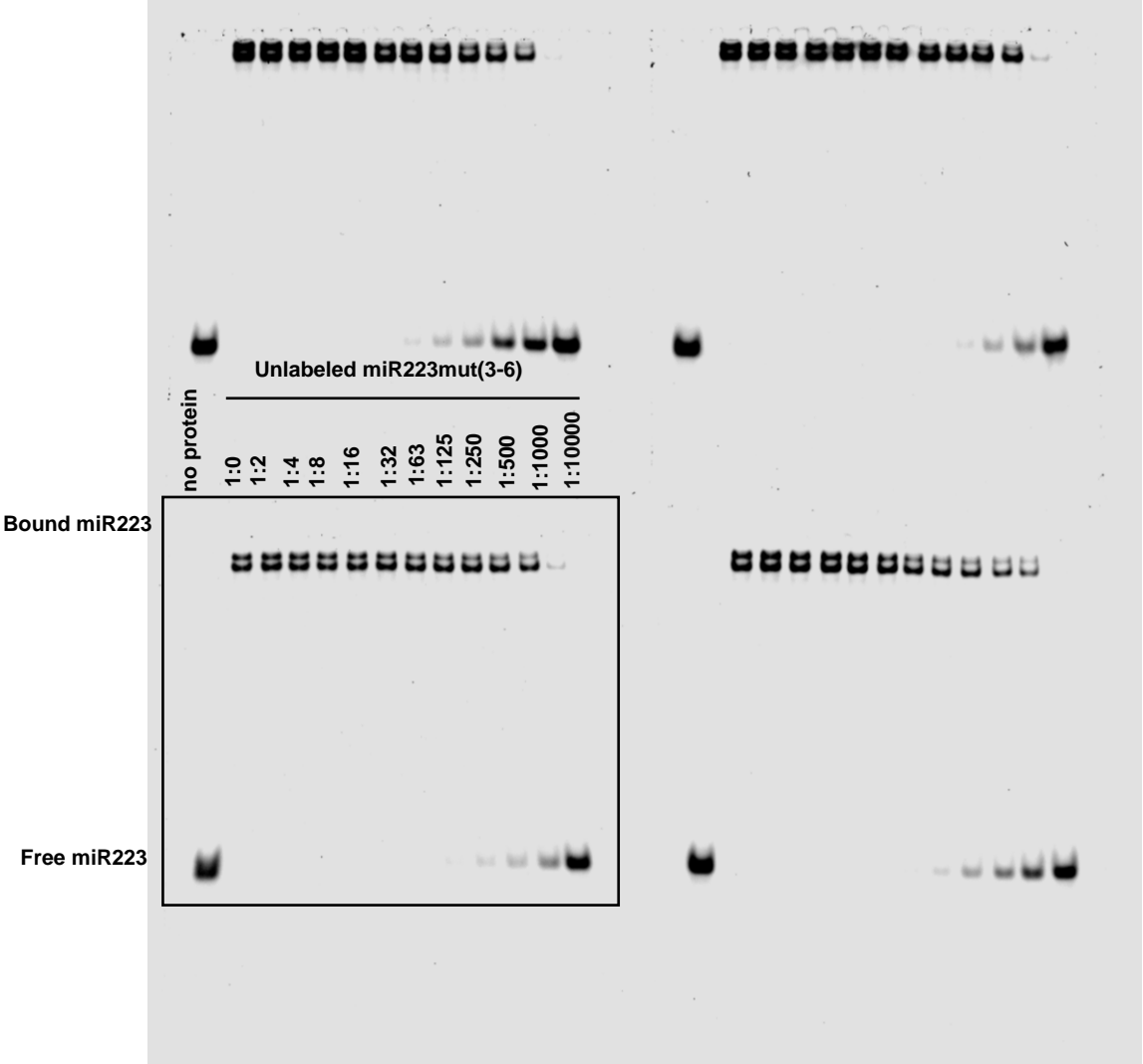

Supplement: Figure 2—figure supplement 1—source data 1. [file elife-85878-fig2-figsupp1-data1.zip › Figure 2-figure supplement 1c-source data 2.pdf]

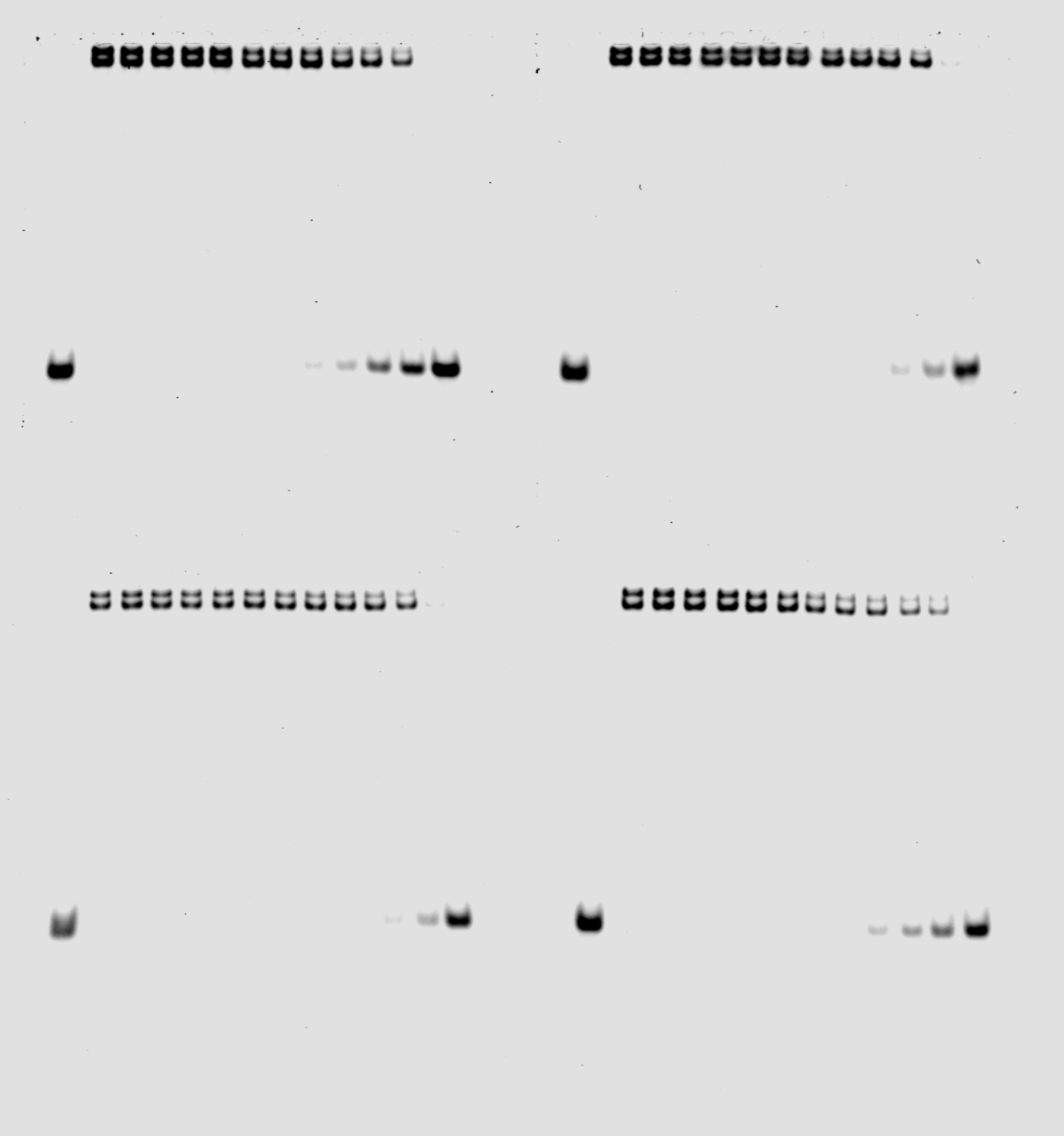

Supplement: Figure 2—figure supplement 1—source data 1. [file elife-85878-fig2-figsupp1-data1.zip › Figure 2-figure supplement 1d-source data 1.tif]

Figure 2-figure supplement 1d

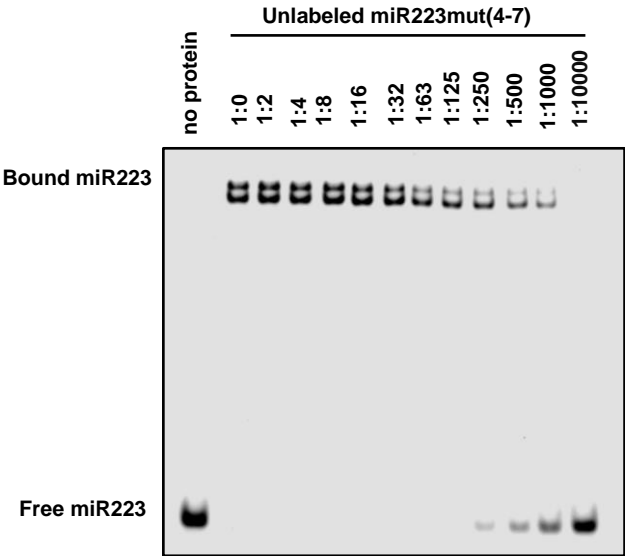

d1.

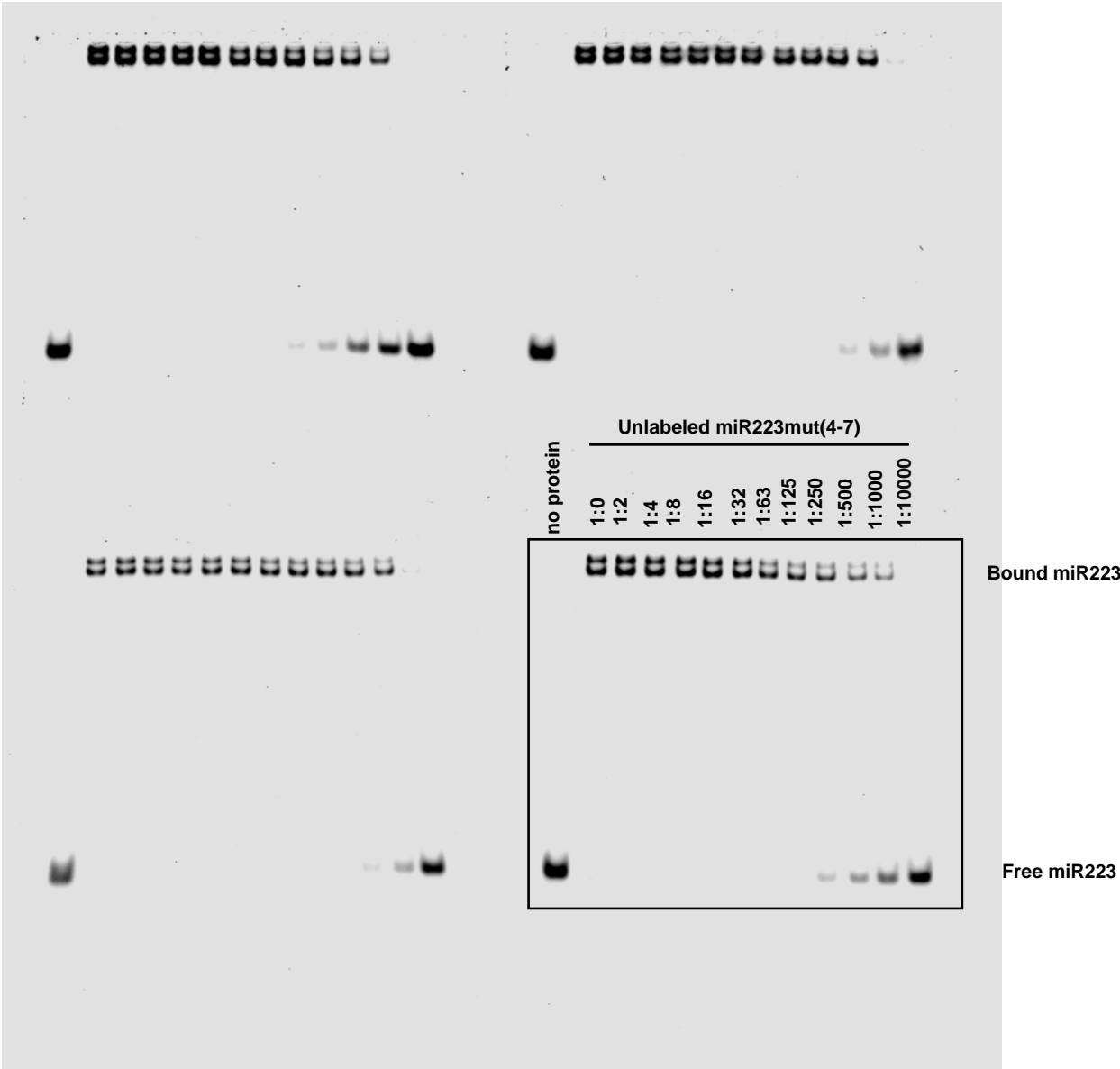

Supplement: Figure 2—figure supplement 1—source data 1. [file elife-85878-fig2-figsupp1-data1.zip › Figure 2-figure supplement 1d-source data 2.pdf]

Figure 2-figure supplement 1e

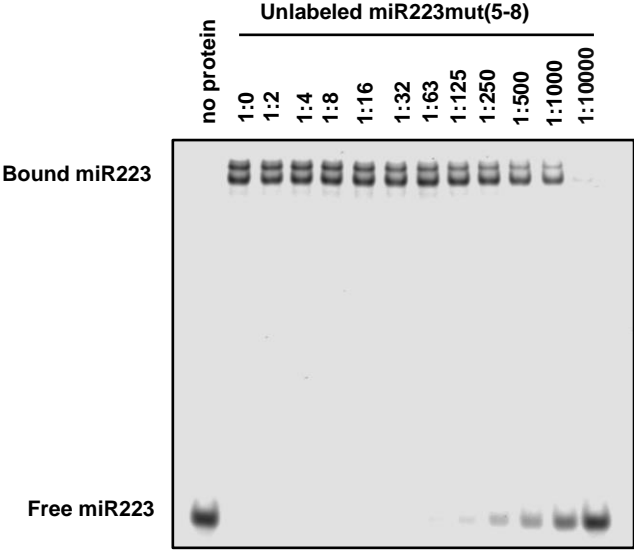

e1.

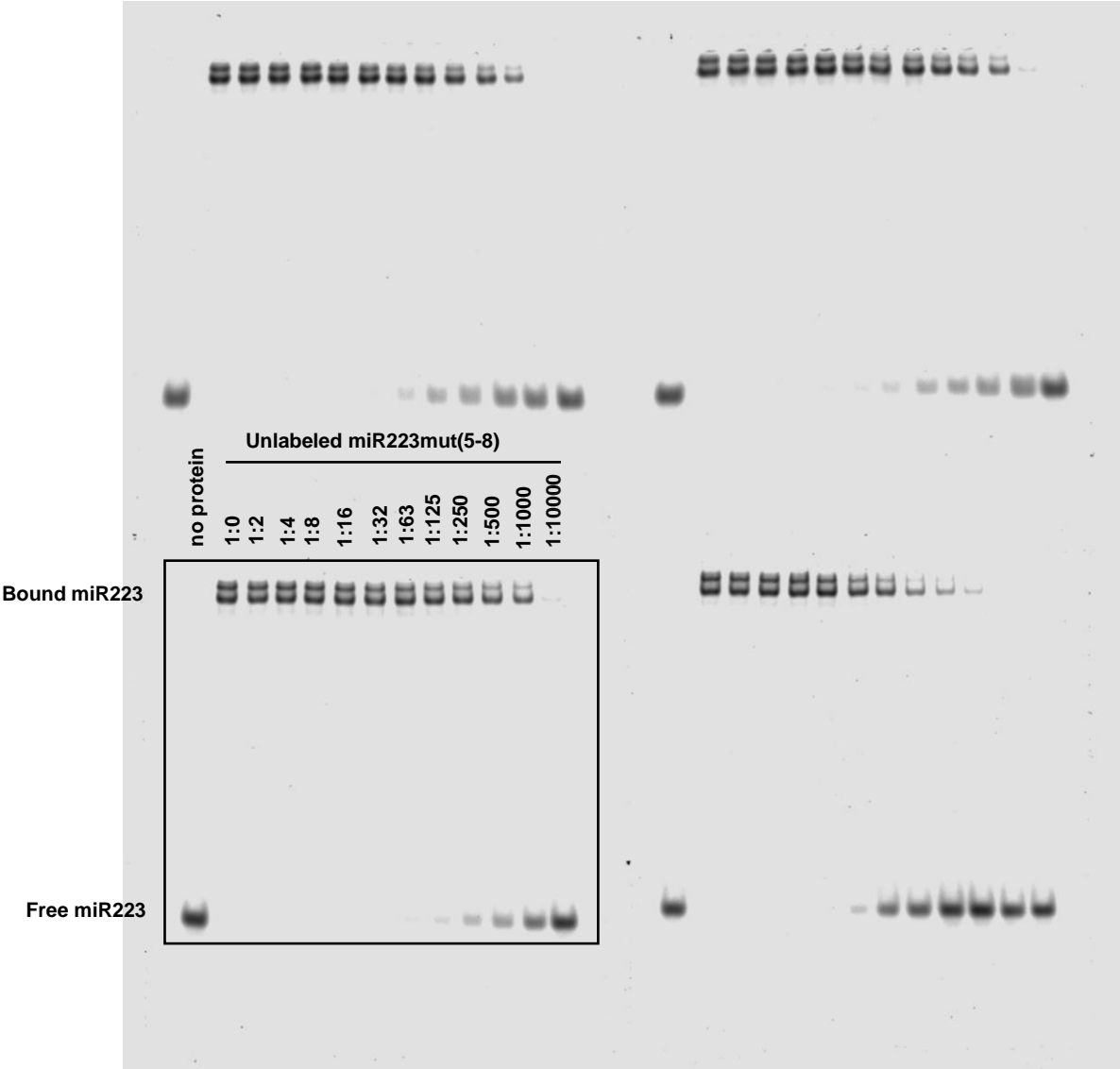

Supplement: Figure 2—figure supplement 1—source data 1. [file elife-85878-fig2-figsupp1-data1.zip › Figure 2-figure supplement 1e-source data 2.pdf]

Figure 2-figure supplement 1f

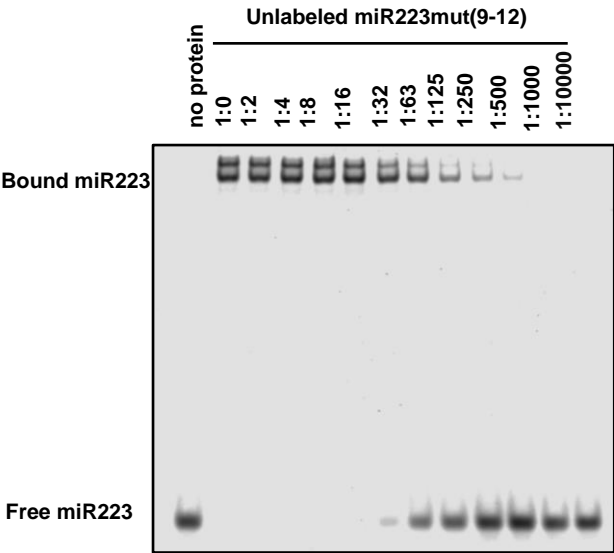

f1.

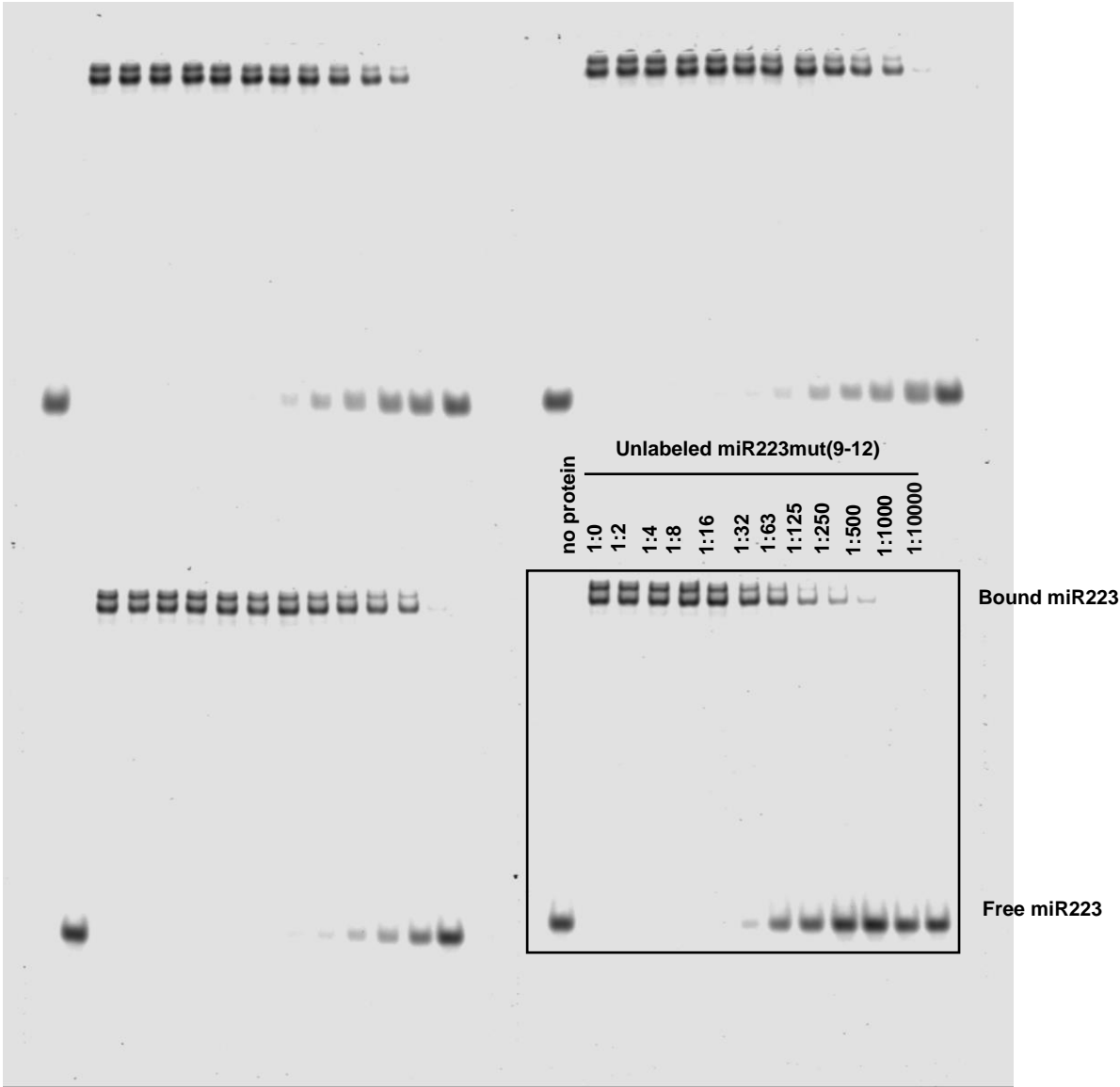

Supplement: Figure 2—figure supplement 1—source data 1. [file elife-85878-fig2-figsupp1-data1.zip › Figure 2-figure supplement 1f-source data 2.pdf]

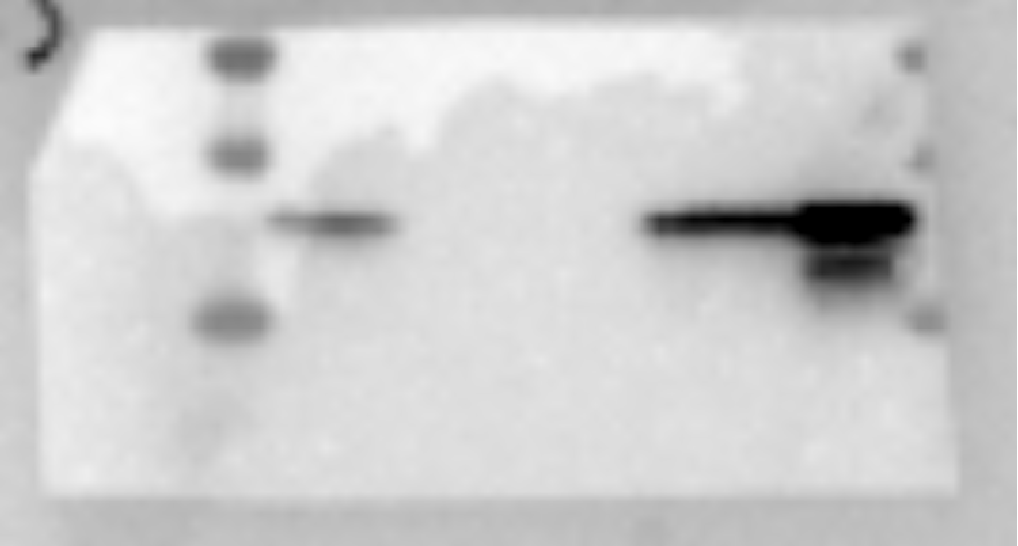

Supplement: Figure 3—source data 1. [file elife-85878-fig3-data1.zip › Figure 3a-source data 1.tif]

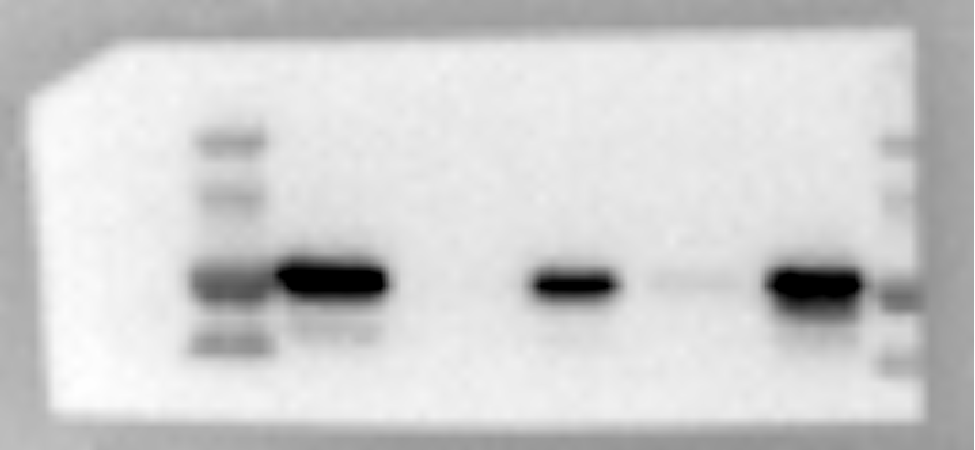

Supplement: Figure 3—source data 1. [file elife-85878-fig3-data1.zip › Figure 3a-source data 2.tif]

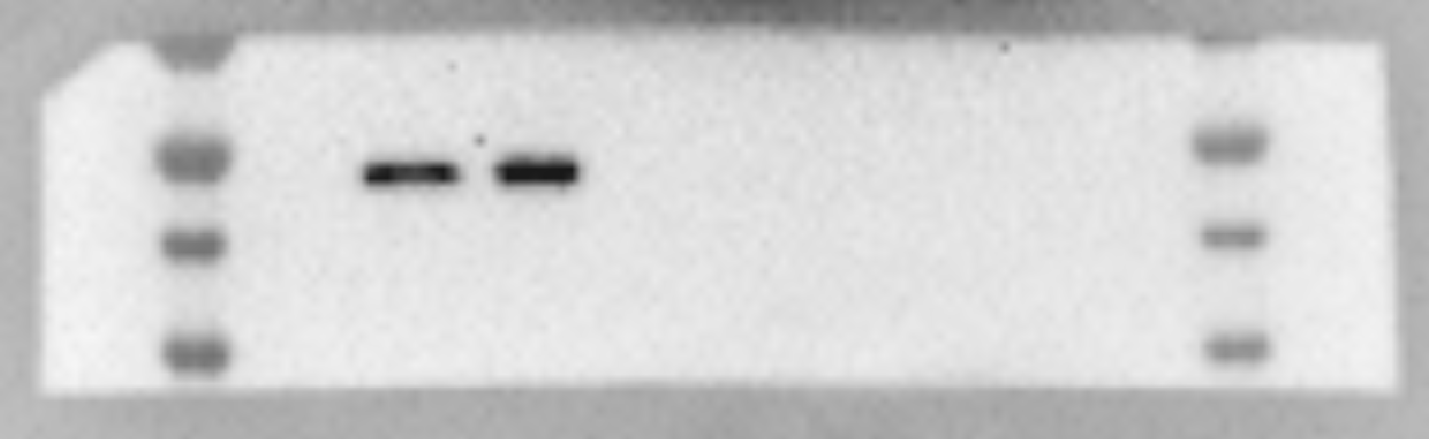

Supplement: Figure 3—source data 1. [file elife-85878-fig3-data1.zip › Figure 3a-source data 3.tif]

Figure 3b.

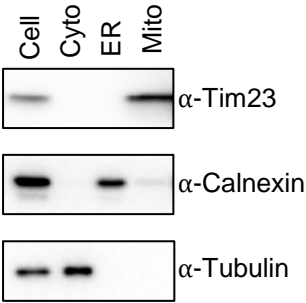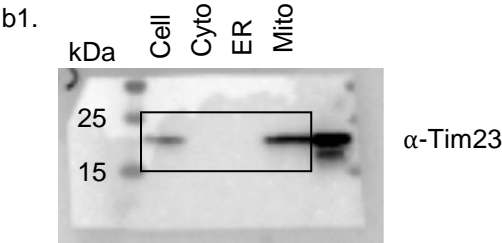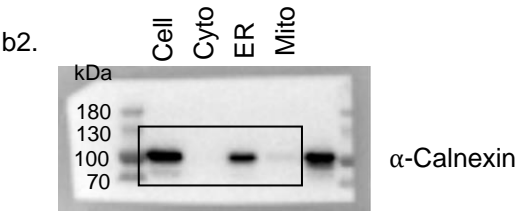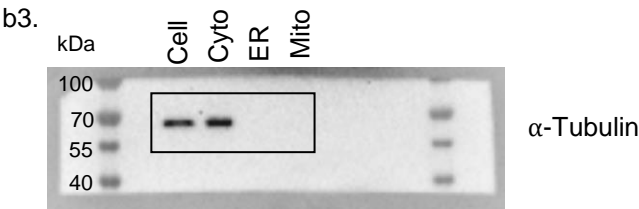

Supplement: Figure 3—source data 1. [file elife-85878-fig3-data1.zip › Figure 3a-source data 4.pdf]

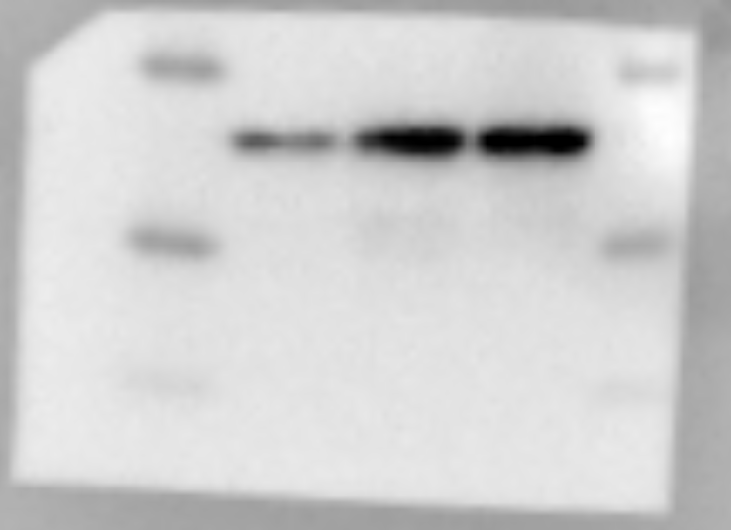

Supplement: Figure 3—source data 1. [file elife-85878-fig3-data1.zip › Figure 3c-source data 1.tif]

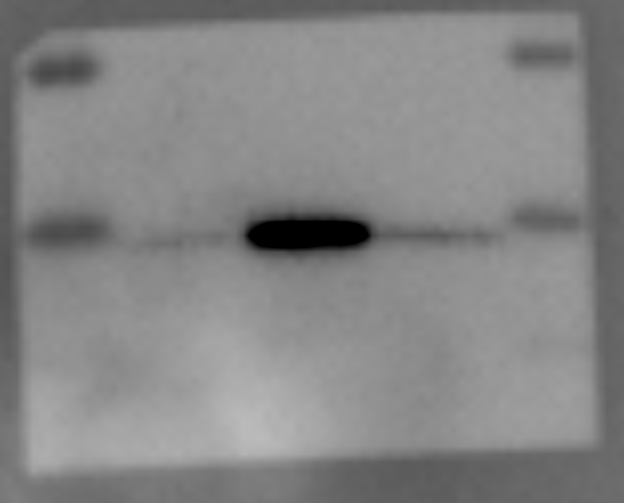

Supplement: Figure 3—source data 1. [file elife-85878-fig3-data1.zip › Figure 3c-source data 2.tif]

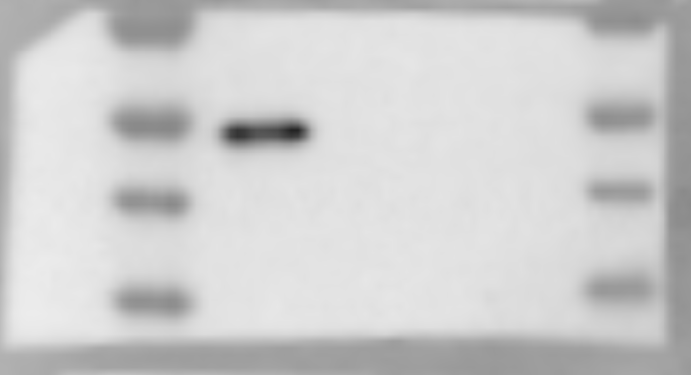

Supplement: Figure 3—source data 1. [file elife-85878-fig3-data1.zip › Figure 3c-source data 3.tif]

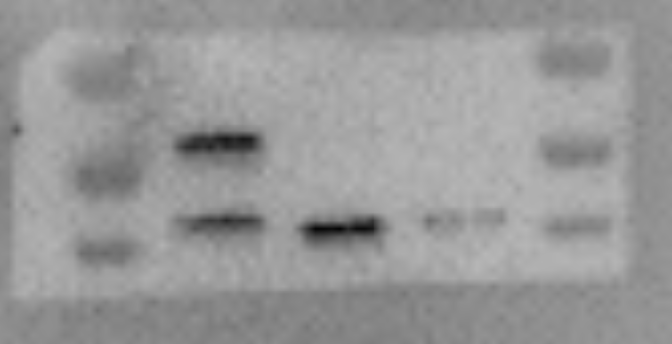

Supplement: Figure 3—source data 1. [file elife-85878-fig3-data1.zip › Figure 3c-source data 4.tif]

Figure 3d.

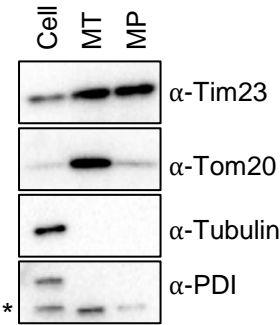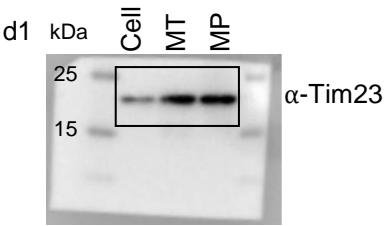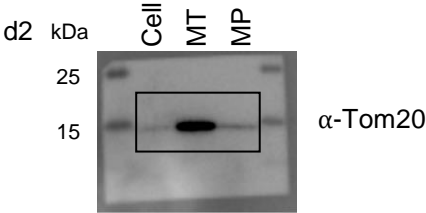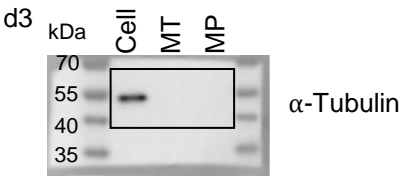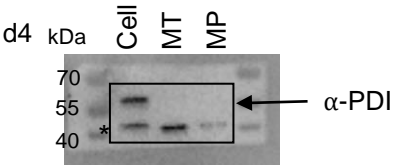

Supplement: Figure 3—source data 1. [file elife-85878-fig3-data1.zip › Figure 3c-source data 5.pdf]

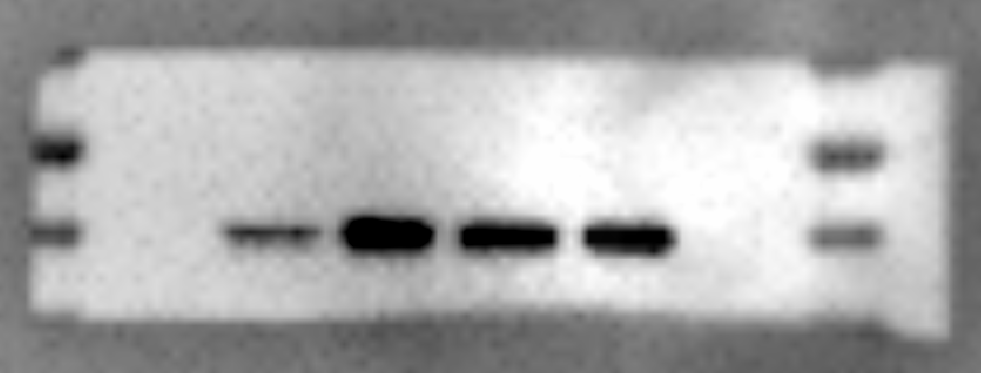

Supplement: Figure 3—source data 1. [file elife-85878-fig3-data1.zip › Figure 3e-source data 1-Citrate Synthase.tif]

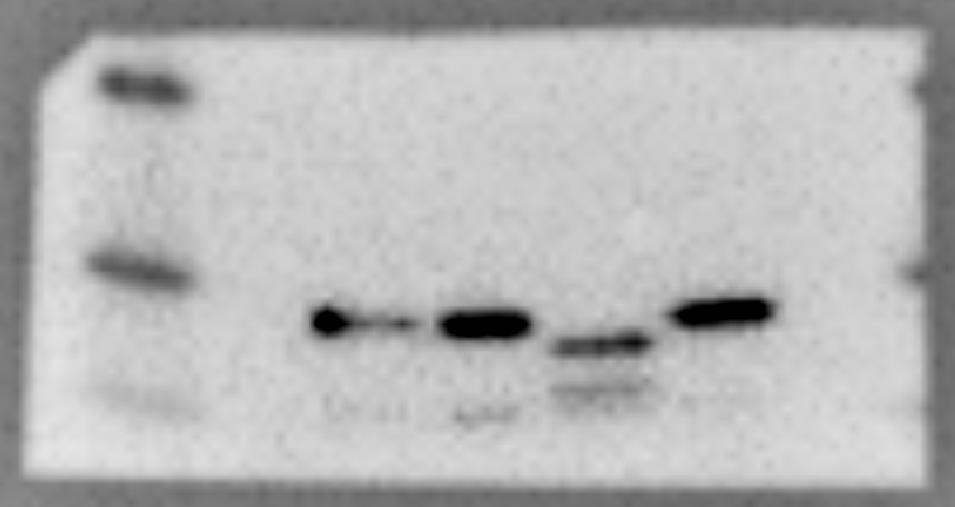

Supplement: Figure 3—source data 1. [file elife-85878-fig3-data1.zip › Figure 3e-source data 2-Tom20.tif]

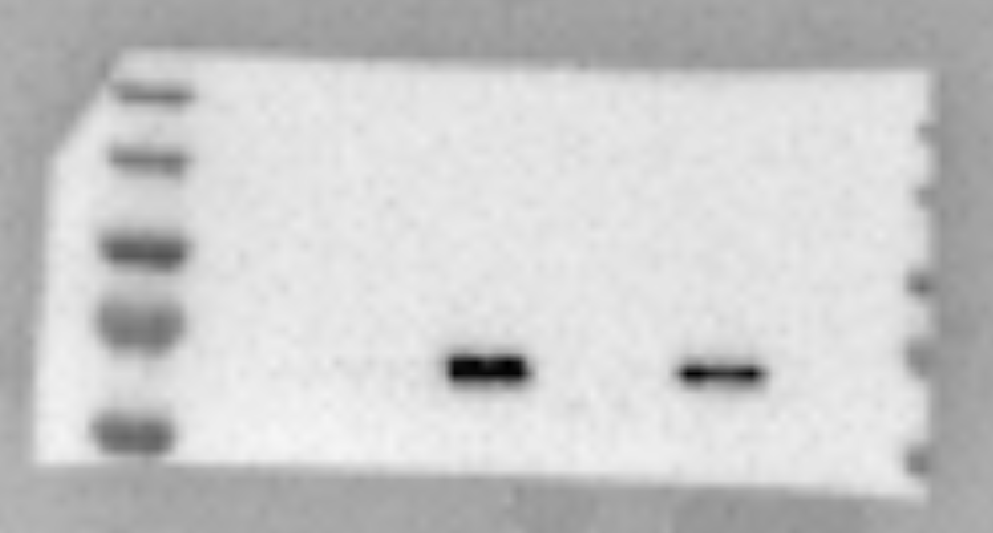

Supplement: Figure 3—source data 1. [file elife-85878-fig3-data1.zip › Figure 3e-source data 3-AIF.tif]

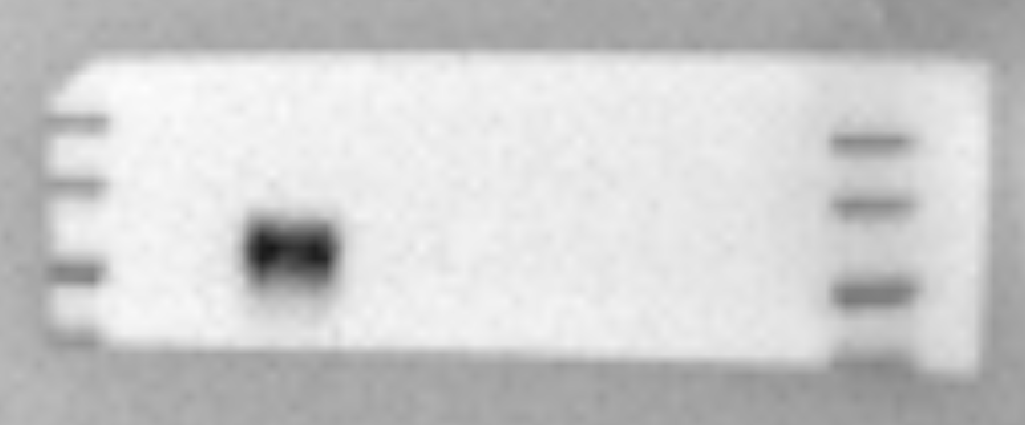

Supplement: Figure 3—source data 1. [file elife-85878-fig3-data1.zip › Figure 3e-source data 4-Lamp1.tif]

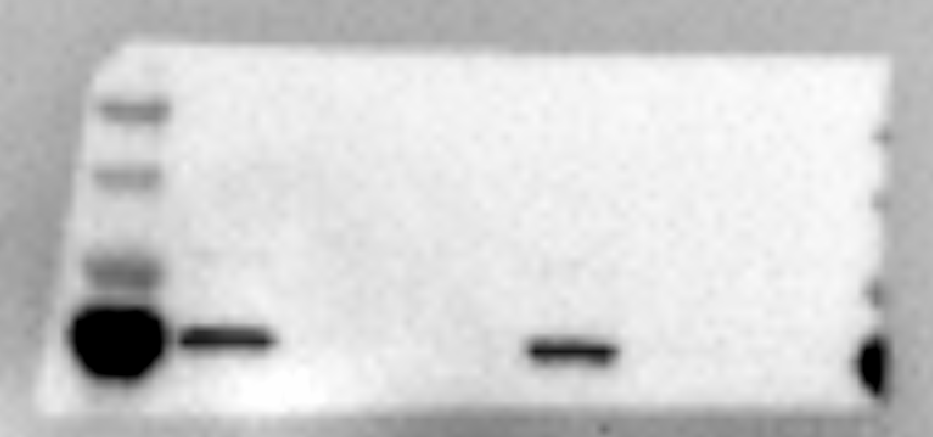

Supplement: Figure 3—source data 1. [file elife-85878-fig3-data1.zip › Figure 3e-source data 5-GRP78.tif]

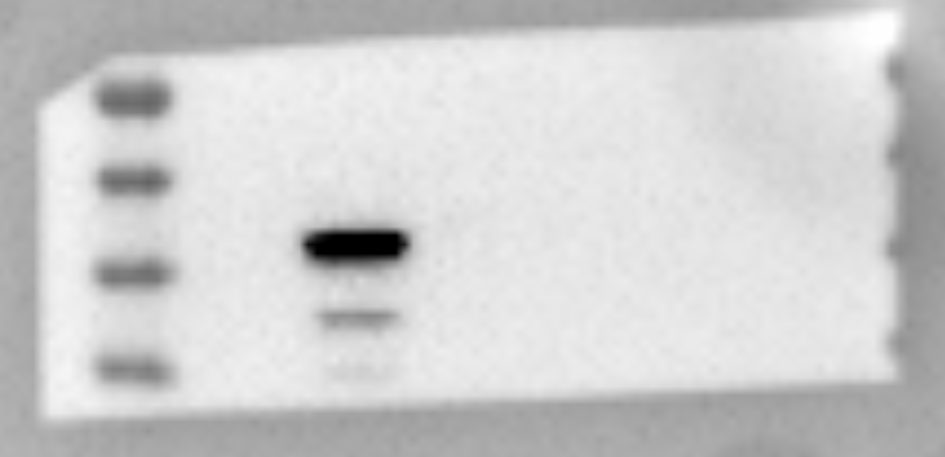

Supplement: Figure 3—source data 1. [file elife-85878-fig3-data1.zip › Figure 3e-source data 6-GAPDH.tif]

Figure 3e.

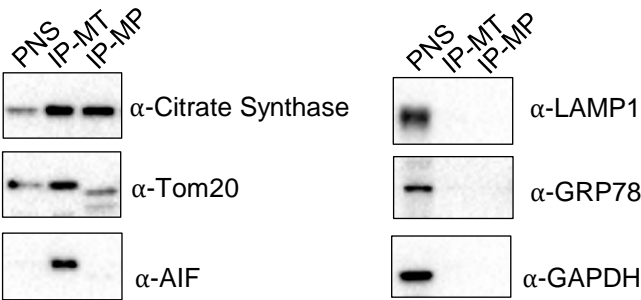

e1

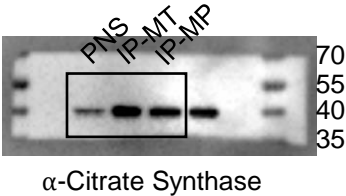

e4

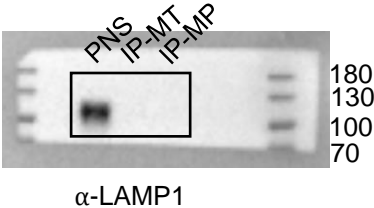

e2

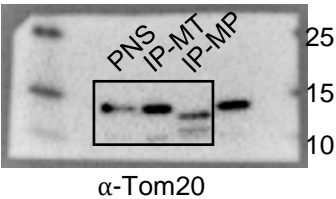

e5

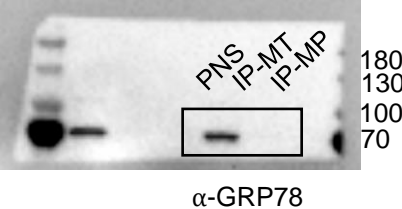

e3

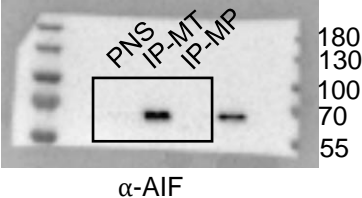

e6

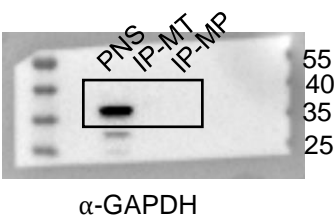

Supplement: Figure 3—source data 1. [file elife-85878-fig3-data1.zip › Figure 3e-source data 7.pdf]

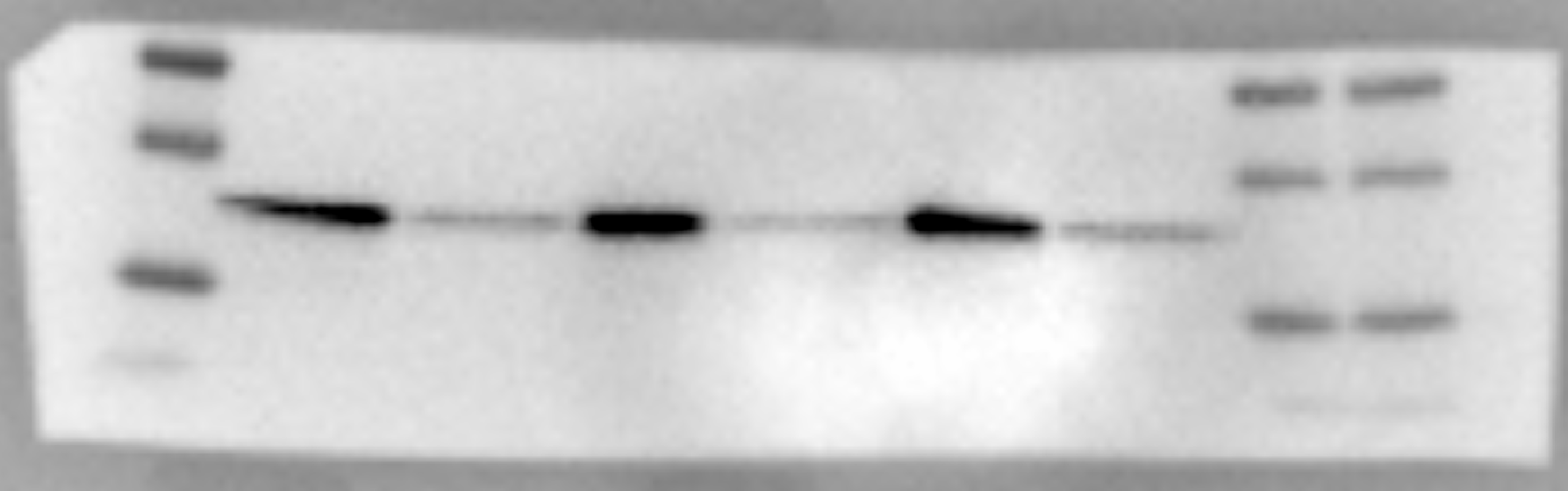

Supplement: Figure 3—source data 1. [file elife-85878-fig3-data1.zip › Figure 3h-source data 1.tif]

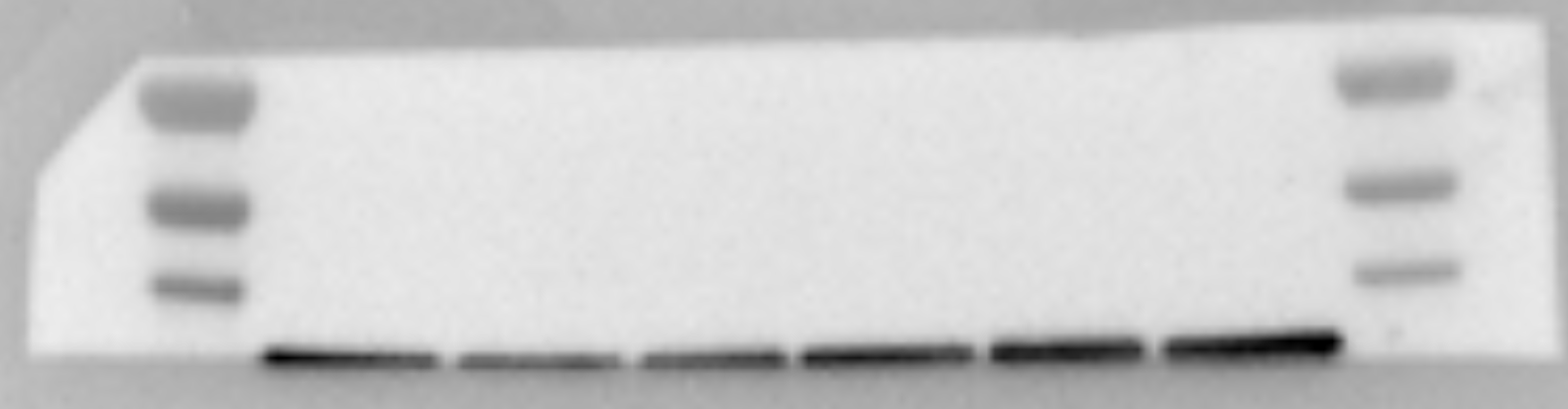

Supplement: Figure 3—source data 1. [file elife-85878-fig3-data1.zip › Figure 3h-source data 2.tif]

Figure 3g.

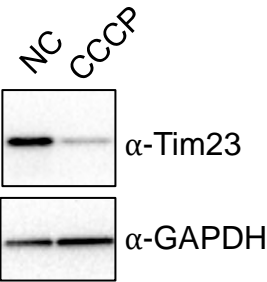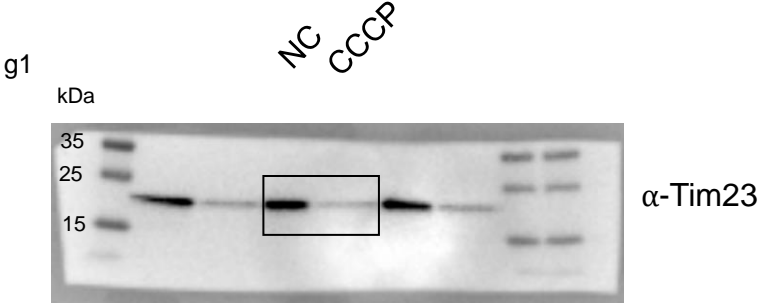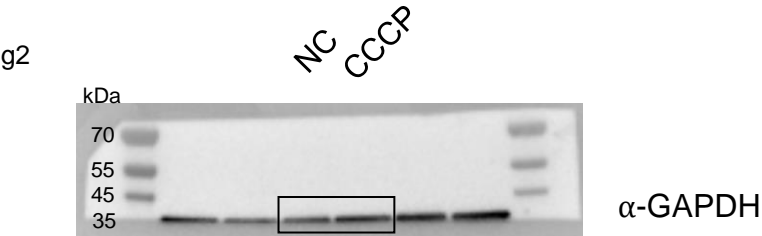

Supplement: Figure 3—source data 1. [file elife-85878-fig3-data1.zip › Figure 3h-source data 3.pdf]

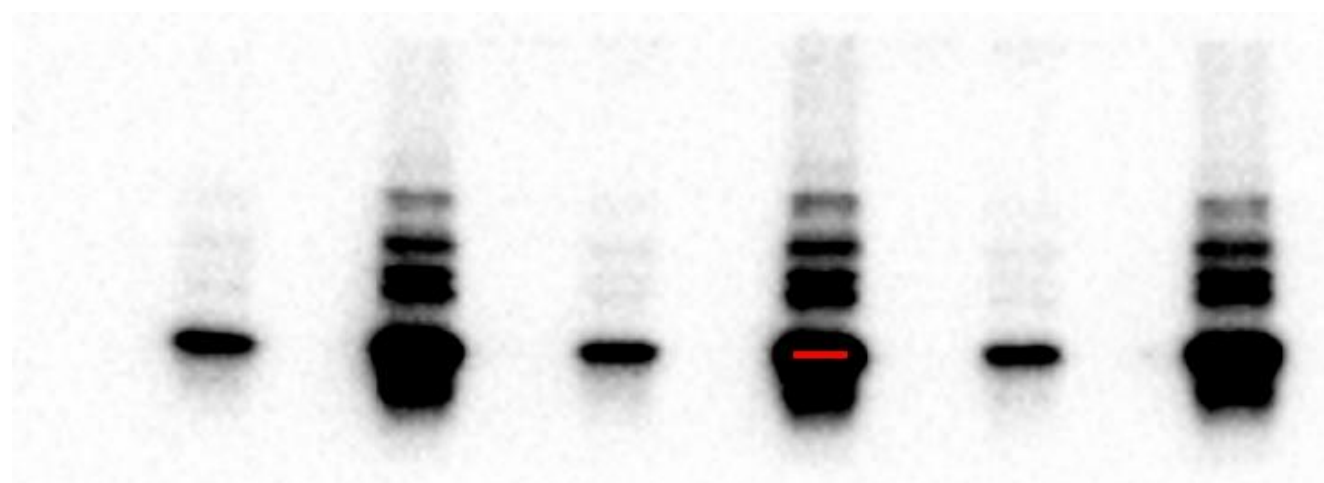

Supplement: Figure 3—figure supplement 1—source data 1. [file elife-85878-fig3-figsupp1-data1.zip › Figure 3-figure supplement 1b-source data 1.pdf]

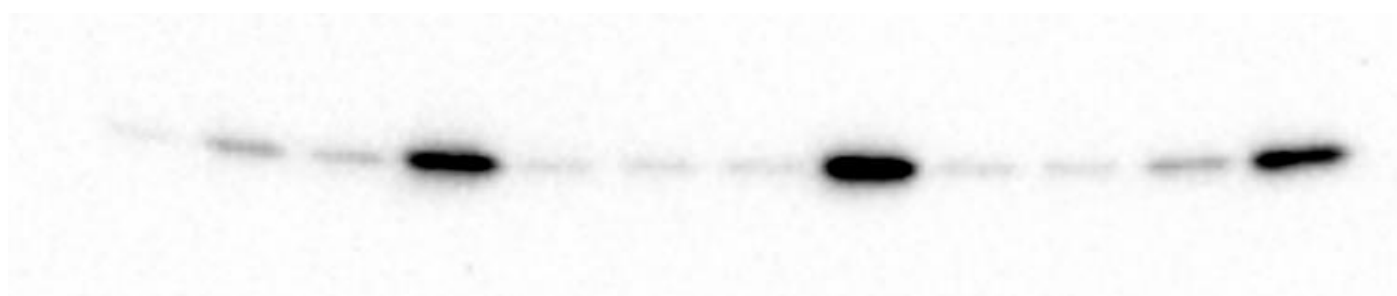

Supplement: Figure 3—figure supplement 1—source data 1. [file elife-85878-fig3-figsupp1-data1.zip › Figure 3-figure supplement 1b-source data 2.pdf]

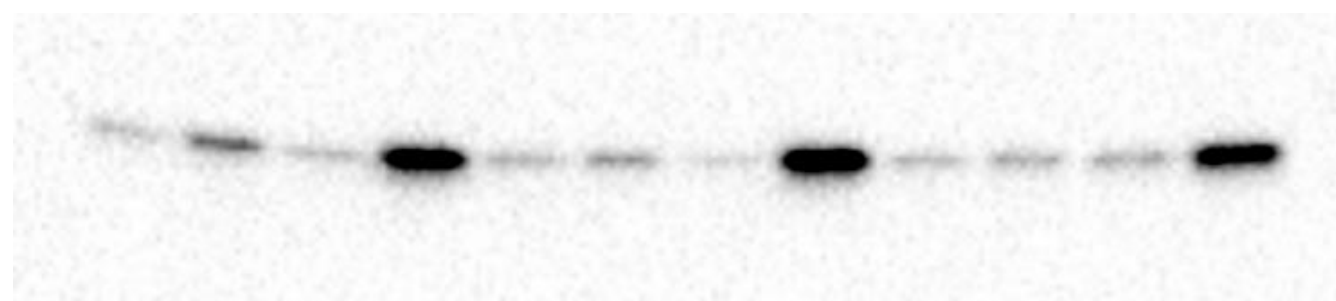

Supplement: Figure 3—figure supplement 1—source data 1. [file elife-85878-fig3-figsupp1-data1.zip › Figure 3-figure supplement 1b-source data 3.pdf]

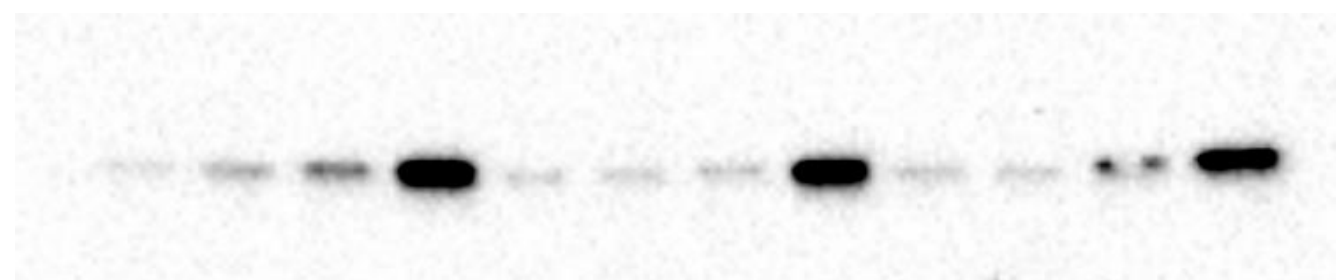

Supplement: Figure 3—figure supplement 1—source data 1. [file elife-85878-fig3-figsupp1-data1.zip › Figure 3-figure supplement 1b-source data 4.pdf]

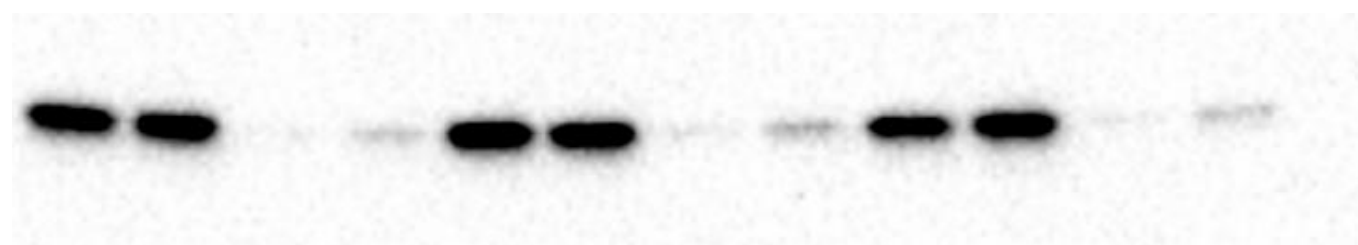

Supplement: Figure 3—figure supplement 1—source data 1. [file elife-85878-fig3-figsupp1-data1.zip › Figure 3-figure supplement 1b-source data 5.pdf]

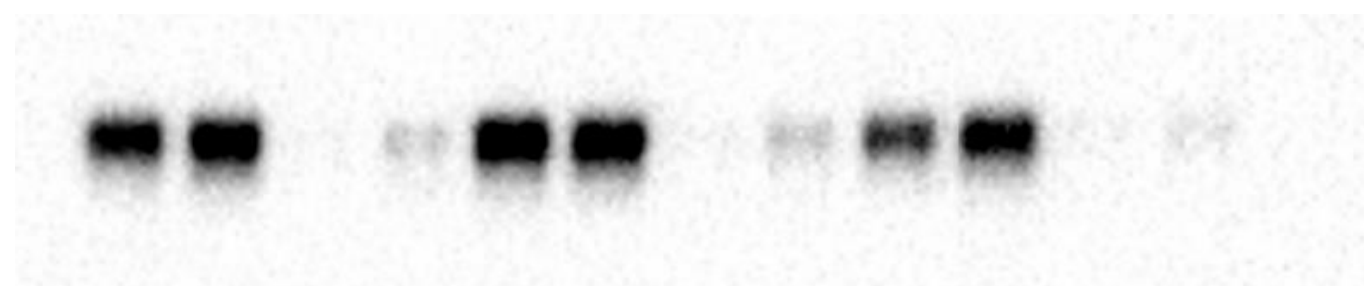

Supplement: Figure 3—figure supplement 1—source data 1. [file elife-85878-fig3-figsupp1-data1.zip › Figure 3-figure supplement 1b-source data 6.pdf]

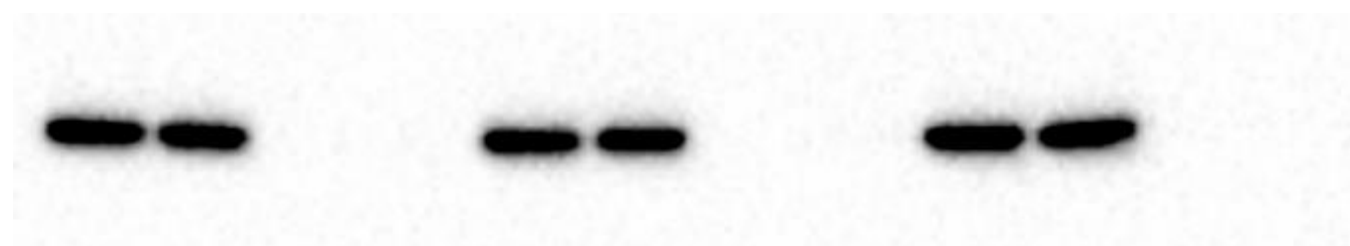

Supplement: Figure 3—figure supplement 1—source data 1. [file elife-85878-fig3-figsupp1-data1.zip › Figure 3-figure supplement 1b-source data 7.pdf]

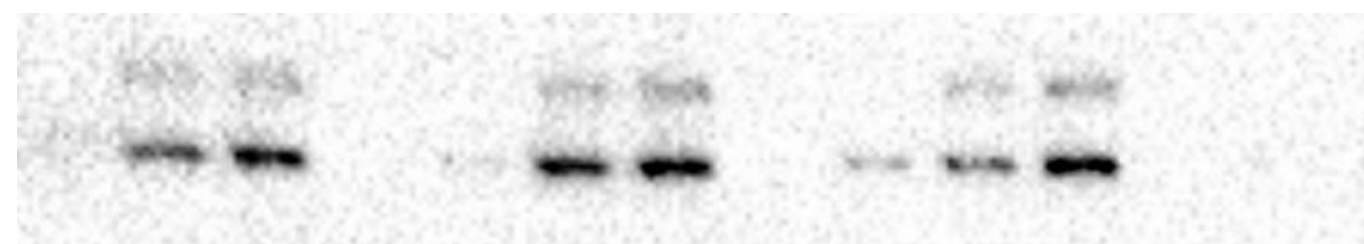

Supplement: Figure 3—figure supplement 1—source data 1. [file elife-85878-fig3-figsupp1-data1.zip › Figure 3-figure supplement 1b-source data 8.pdf]

Figure 3-figure supplement 1b

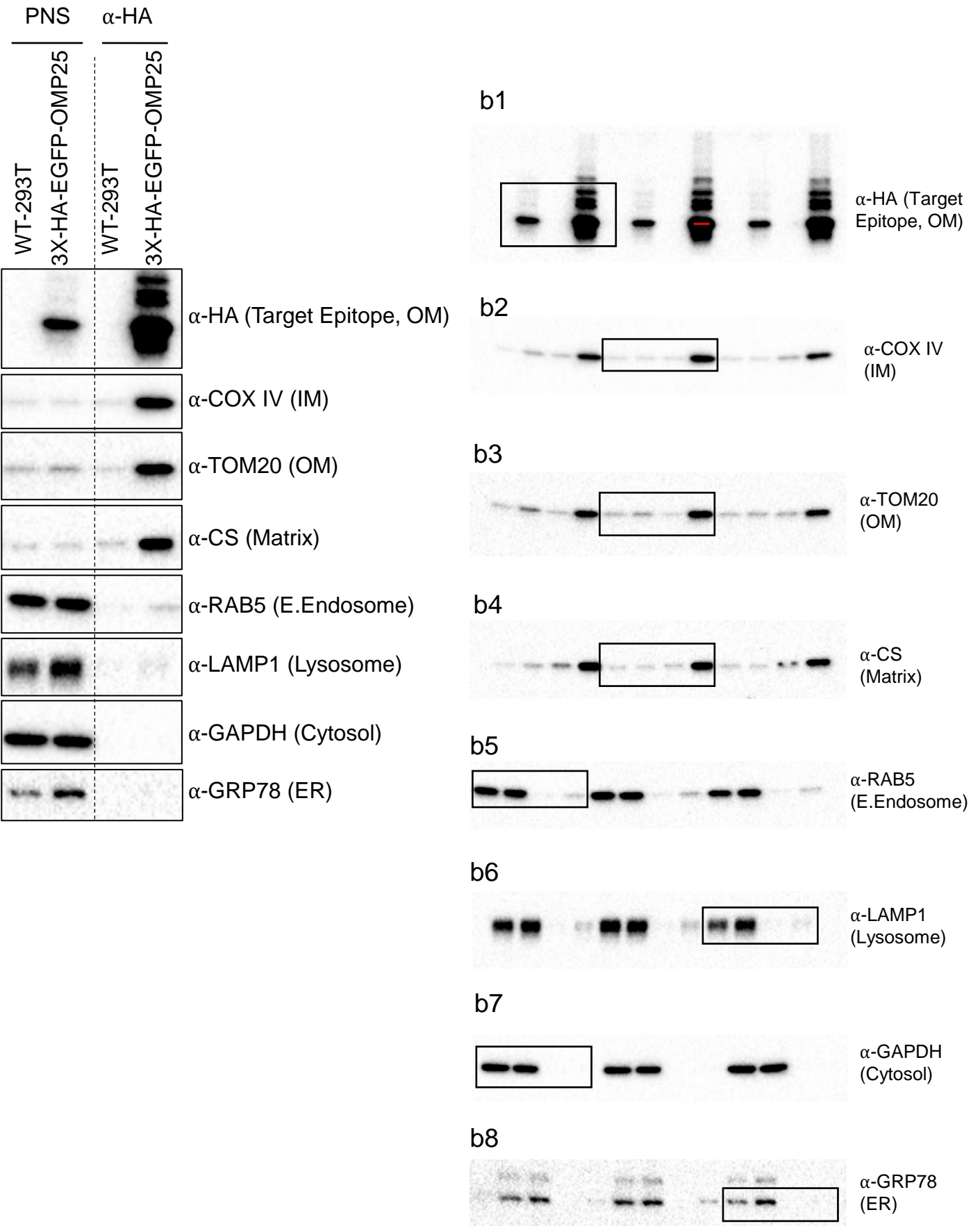

Supplement: Figure 3—figure supplement 1—source data 1. [file elife-85878-fig3-figsupp1-data1.zip › Figure 3-figure supplement 1b-source data 9.pdf]

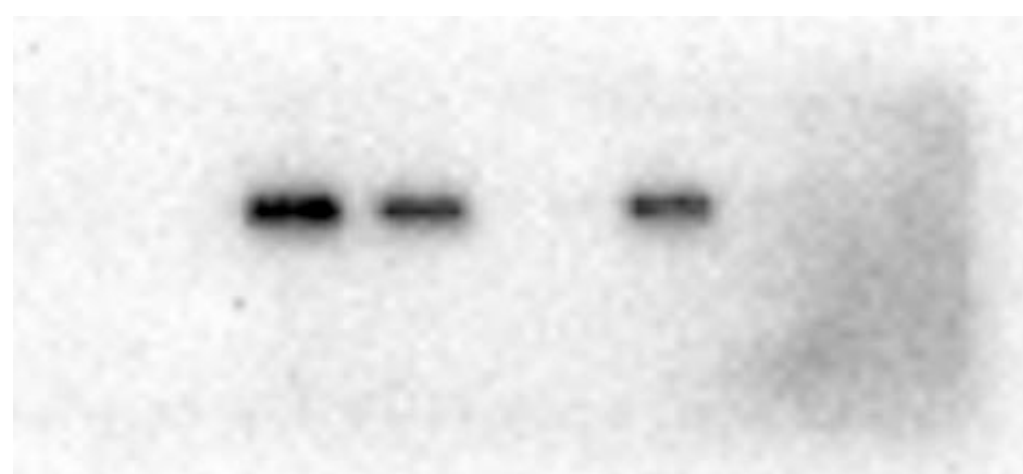

Supplement: Figure 3—figure supplement 1—source data 1. [file elife-85878-fig3-figsupp1-data1.zip › Figure 3-figure supplement 1c-source data 1.pdf]

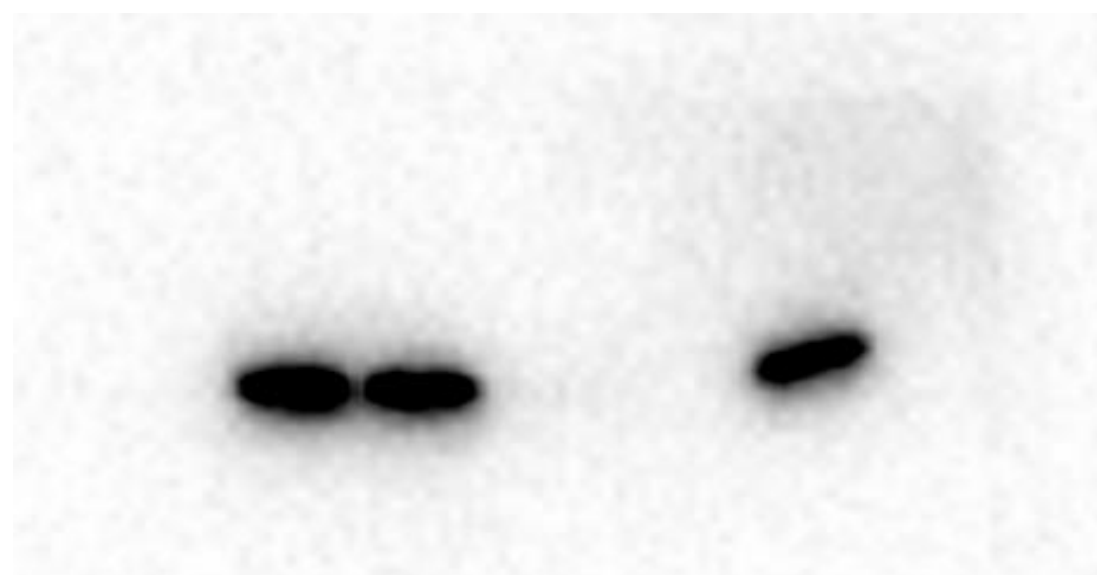

Supplement: Figure 3—figure supplement 1—source data 1. [file elife-85878-fig3-figsupp1-data1.zip › Figure 3-figure supplement 1c-source data 2.pdf]

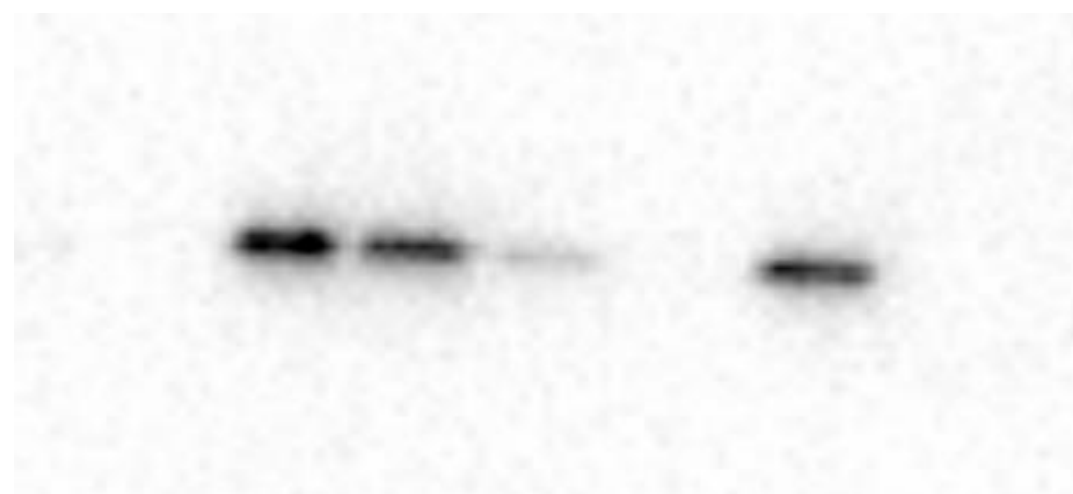

Supplement: Figure 3—figure supplement 1—source data 1. [file elife-85878-fig3-figsupp1-data1.zip › Figure 3-figure supplement 1c-source data 3.pdf]

Figure 3-figure supplement 1c

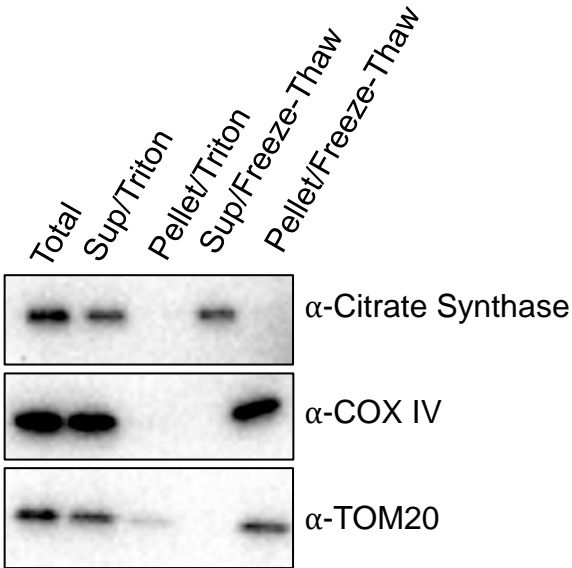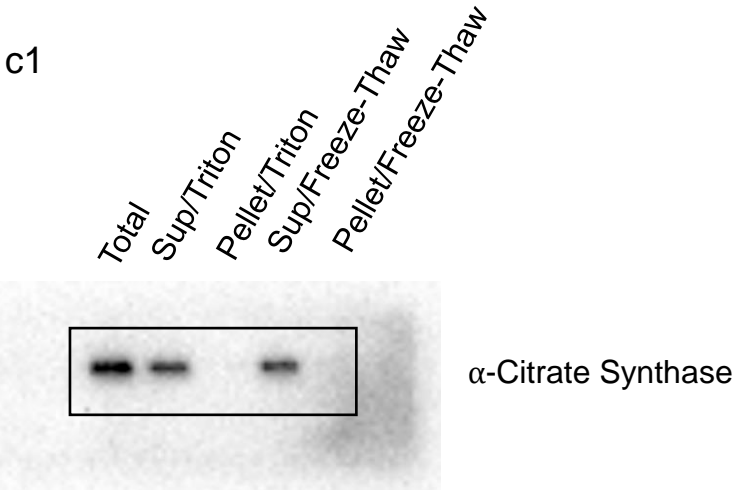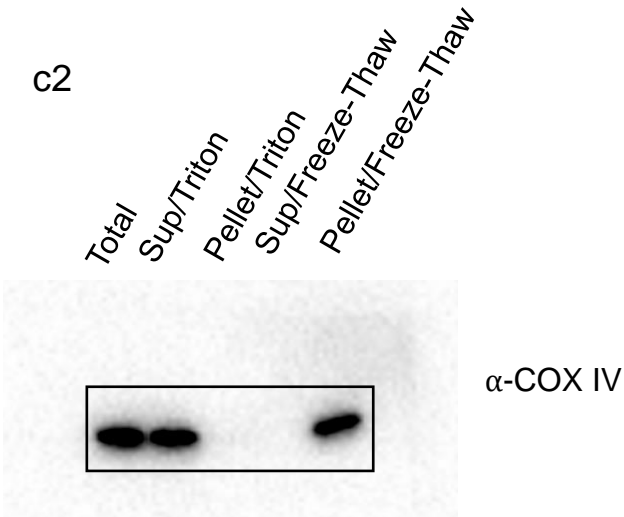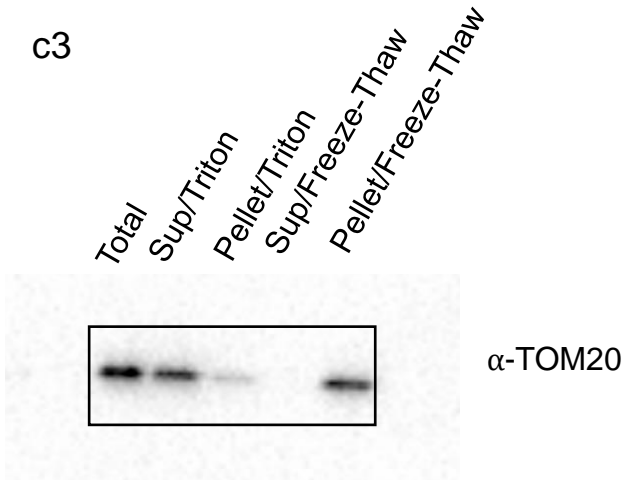

Supplement: Figure 3—figure supplement 1—source data 1. [file elife-85878-fig3-figsupp1-data1.zip › Figure 3-figure supplement 1c-source data 4.pdf]

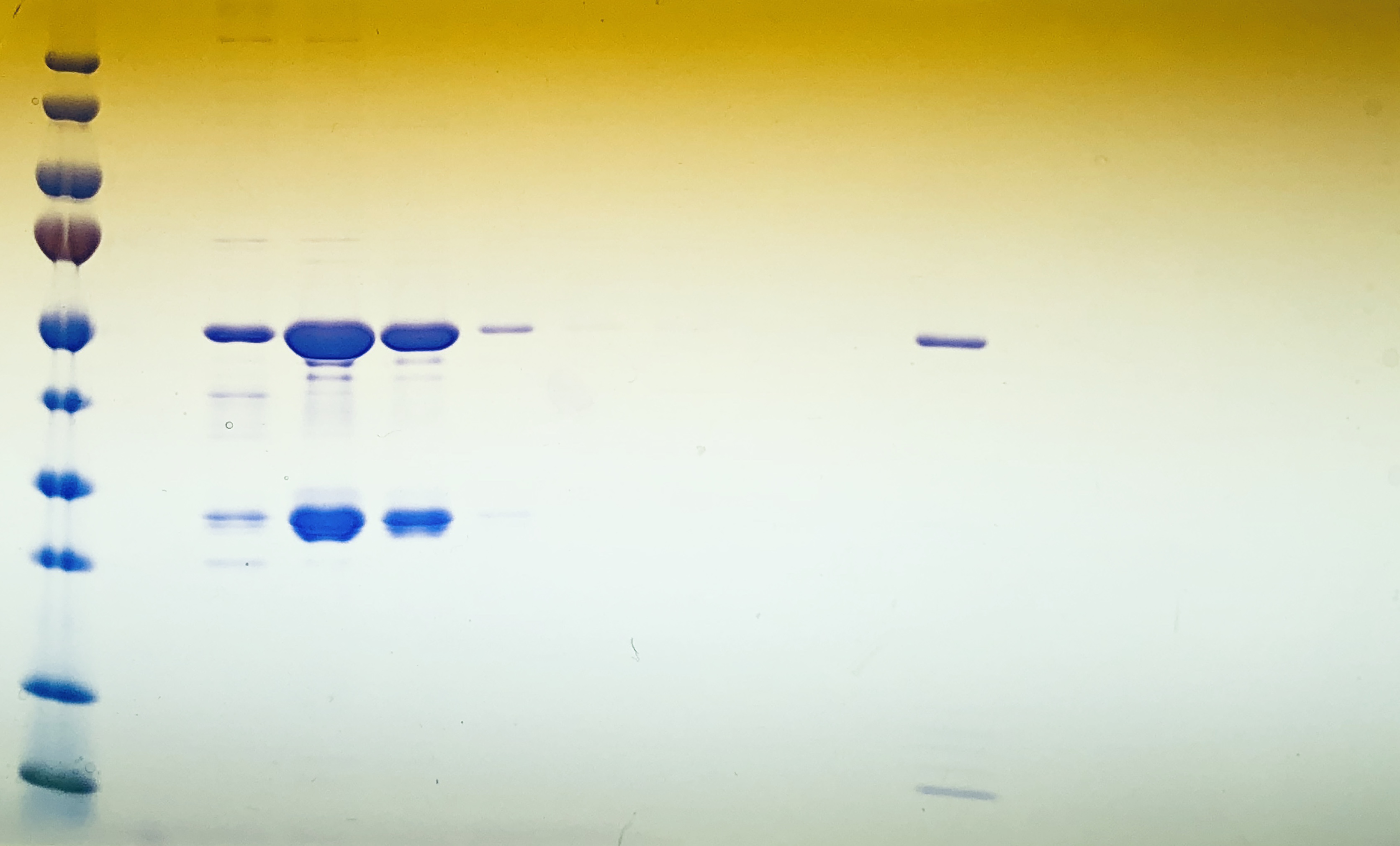

Supplement: Figure 4—source data 1. [file elife-85878-fig4-data1.zip › Figure 4a-source data 1.tif]

Figure 4a

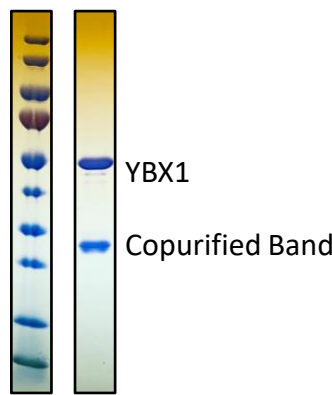

a1

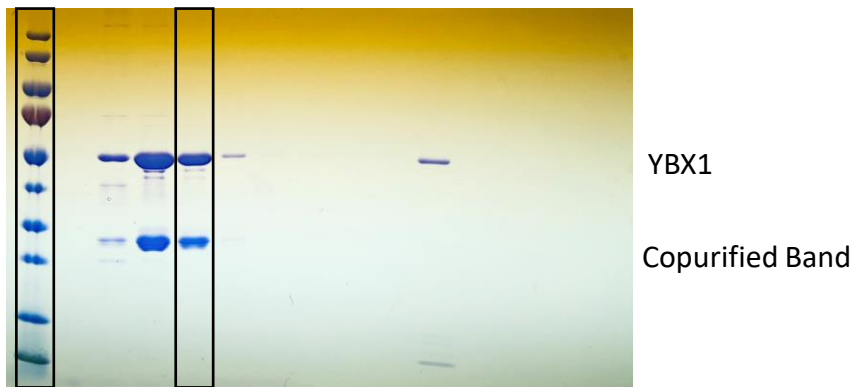

Supplement: Figure 4—source data 1. [file elife-85878-fig4-data1.zip › Figure 4a-source data 2.pdf]

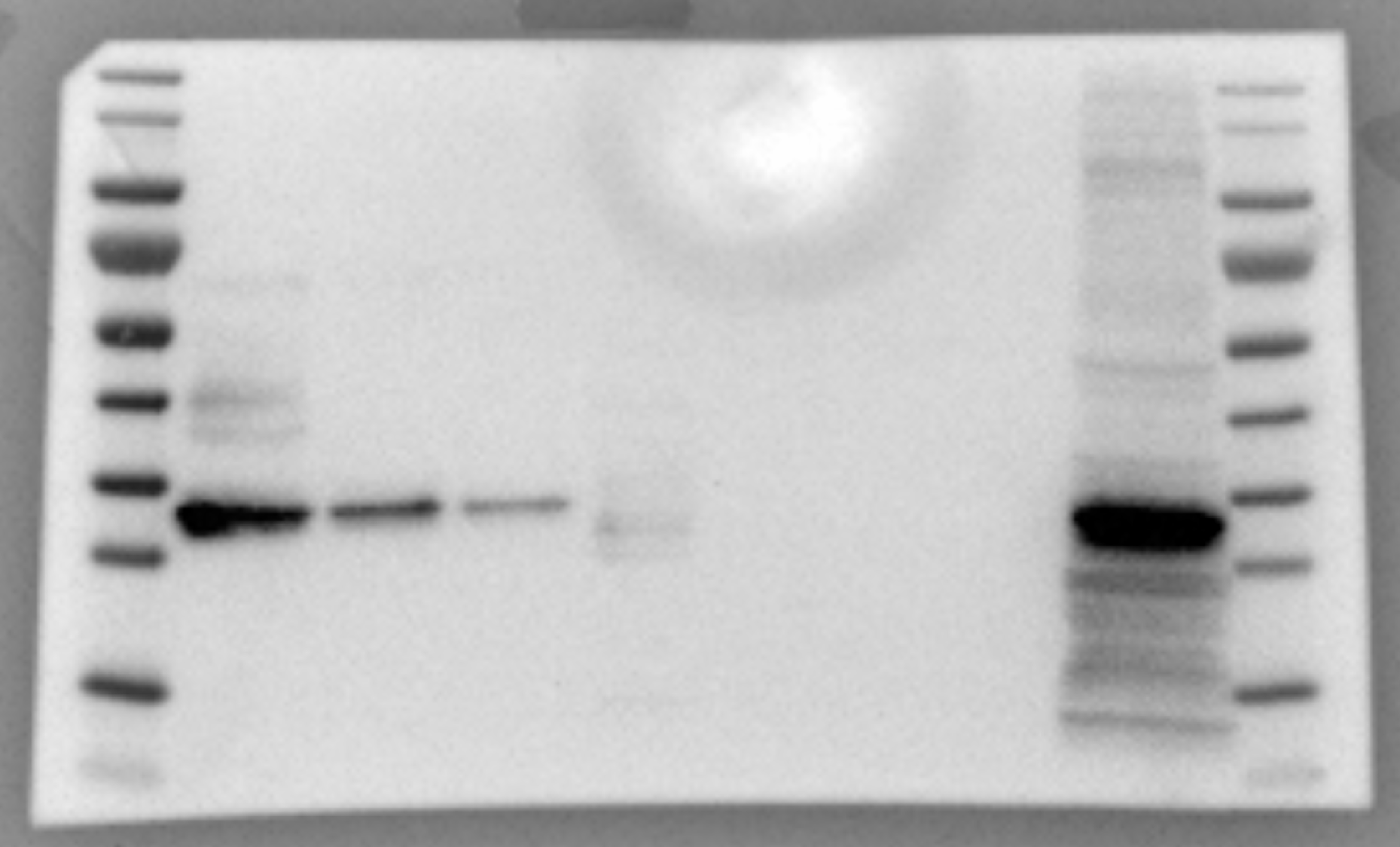

Supplement: Figure 4—source data 1. [file elife-85878-fig4-data1.zip › Figure 4b-source data 1.tif]

Figure 4b

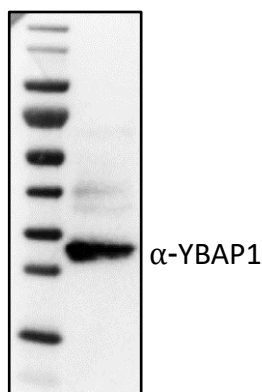

b1

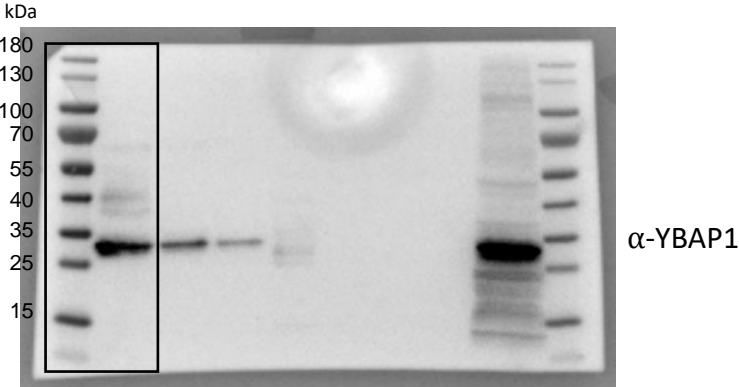

Supplement: Figure 4—source data 1. [file elife-85878-fig4-data1.zip › Figure 4b-source data 2.pdf]

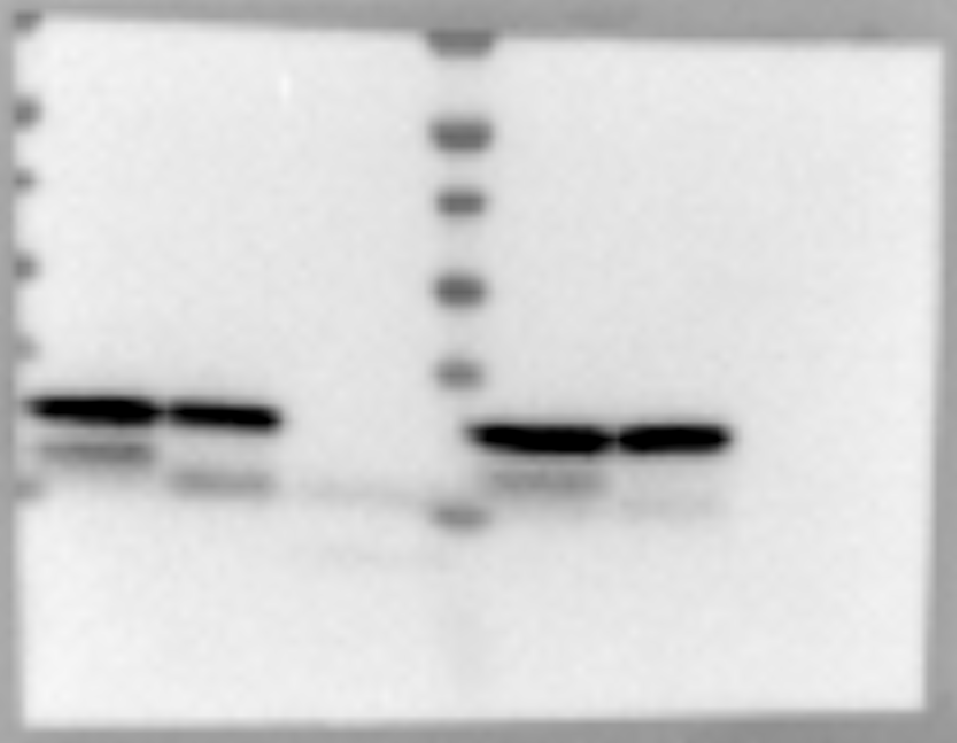

Supplement: Figure 4—source data 1. [file elife-85878-fig4-data1.zip › Figure 4e-source data 1.tif]

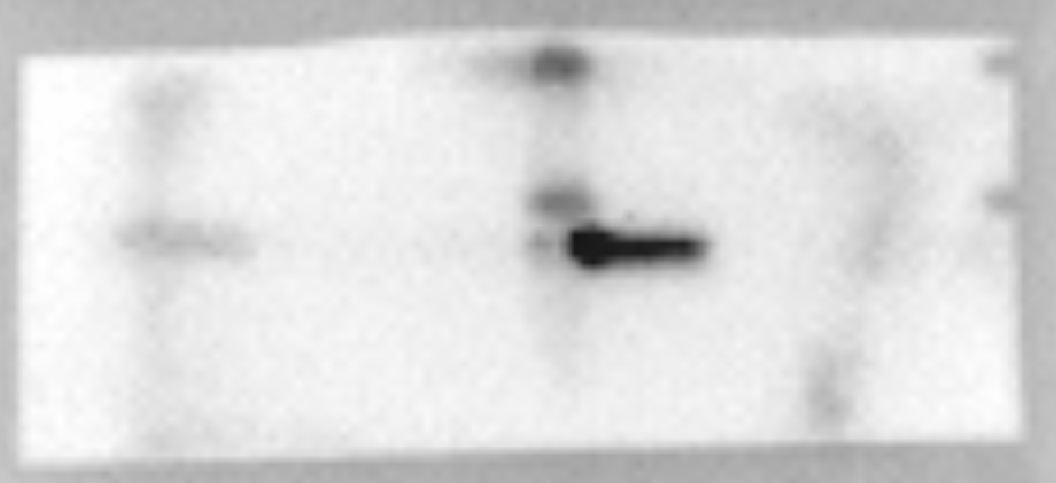

Supplement: Figure 4—source data 1. [file elife-85878-fig4-data1.zip › Figure 4e-source data 2.tif]

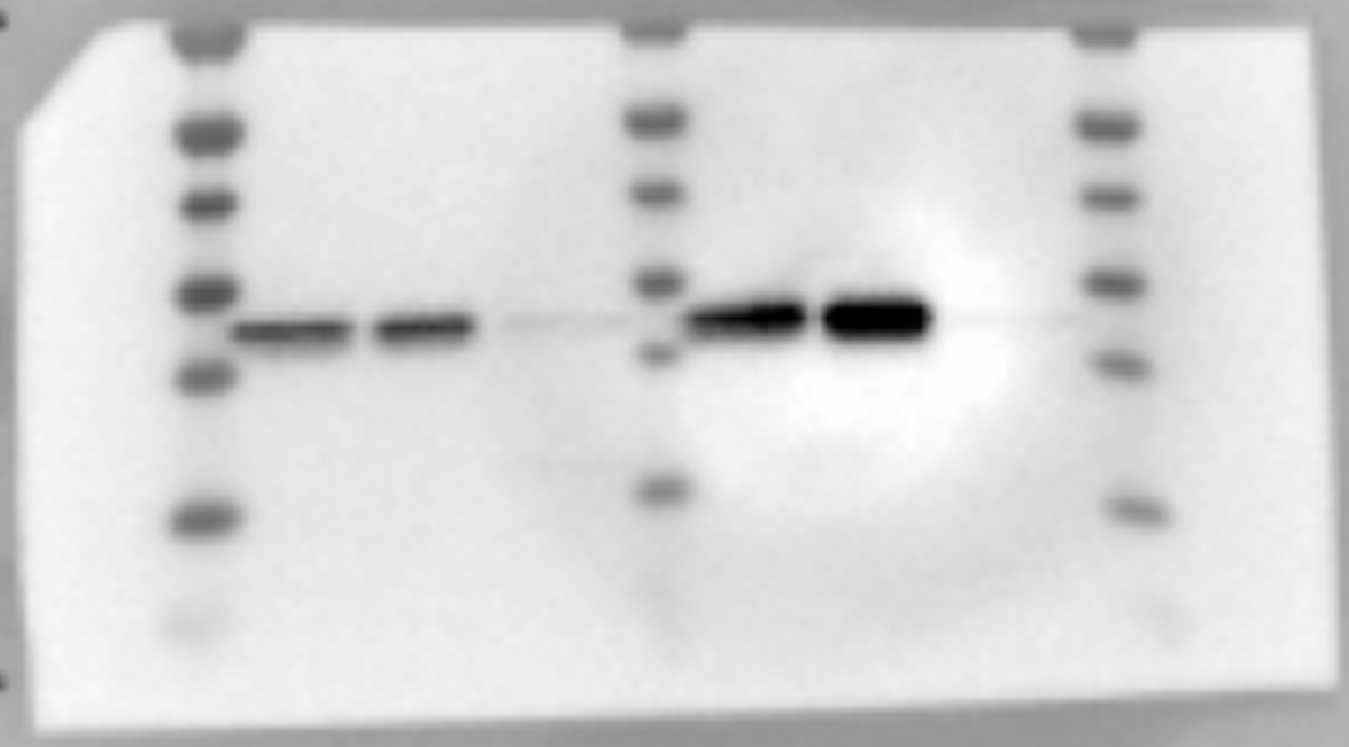

Supplement: Figure 4—source data 1. [file elife-85878-fig4-data1.zip › Figure 4e-source data 3.tif]

Figure 4e

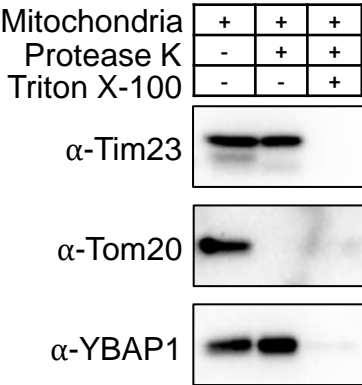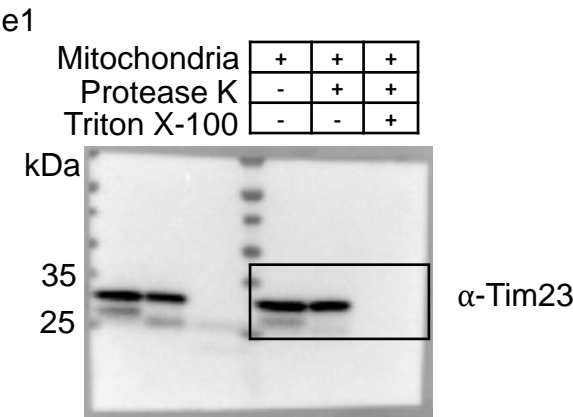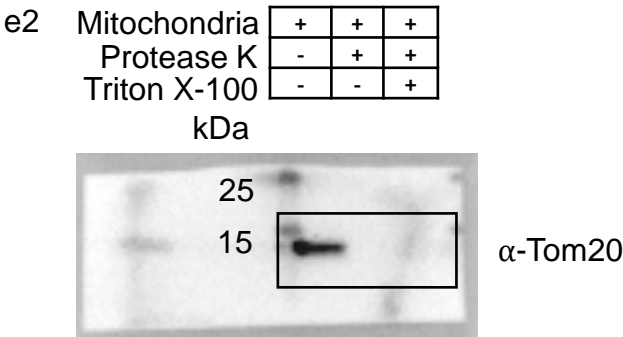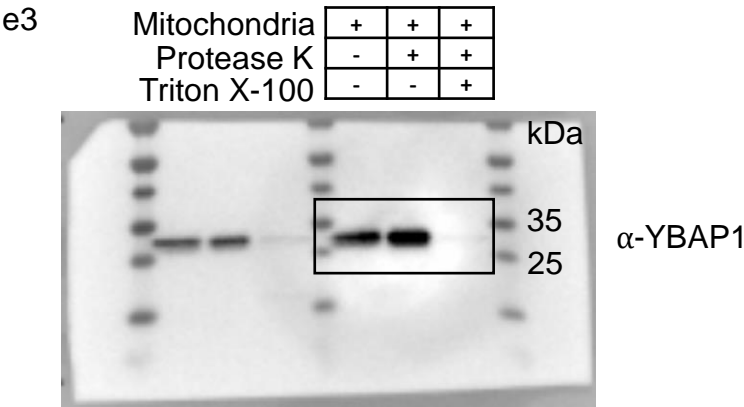

Supplement: Figure 4—source data 1. [file elife-85878-fig4-data1.zip › Figure 4e-source data 4.pdf]

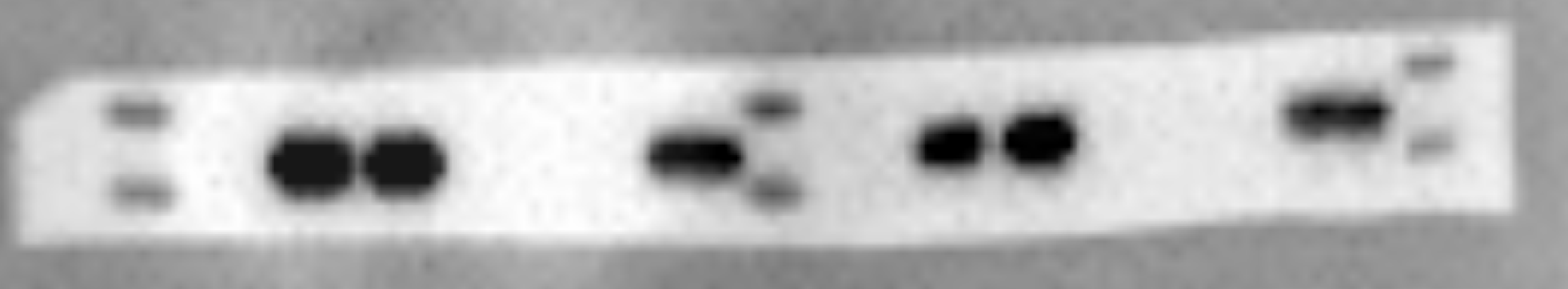

Supplement: Figure 4—source data 1. [file elife-85878-fig4-data1.zip › Figure 4f-source data 1.tif]

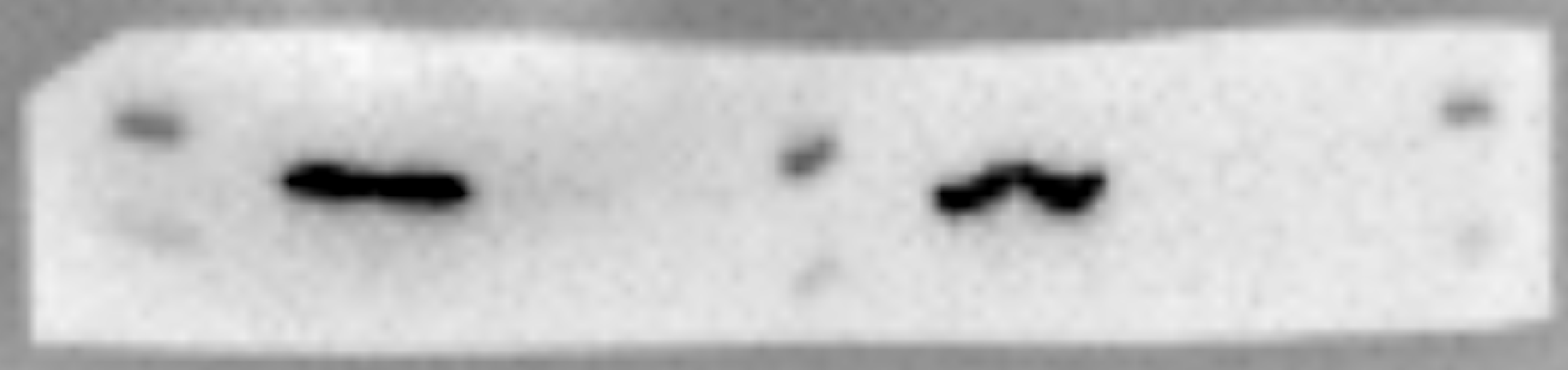

Supplement: Figure 4—source data 1. [file elife-85878-fig4-data1.zip › Figure 4f-source data 2.tif]

Figure 4f

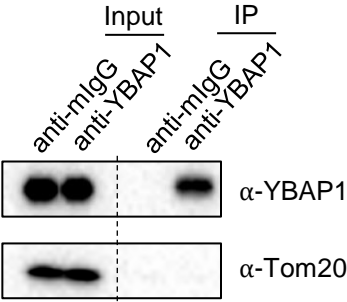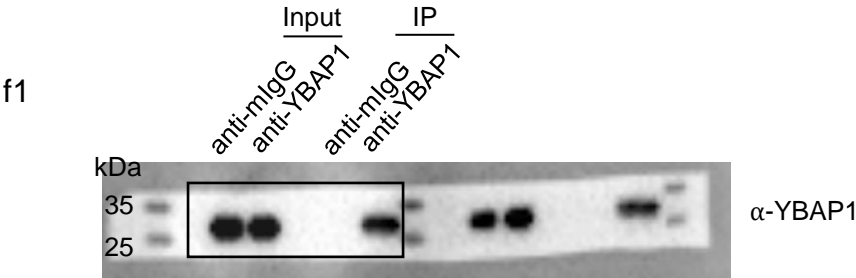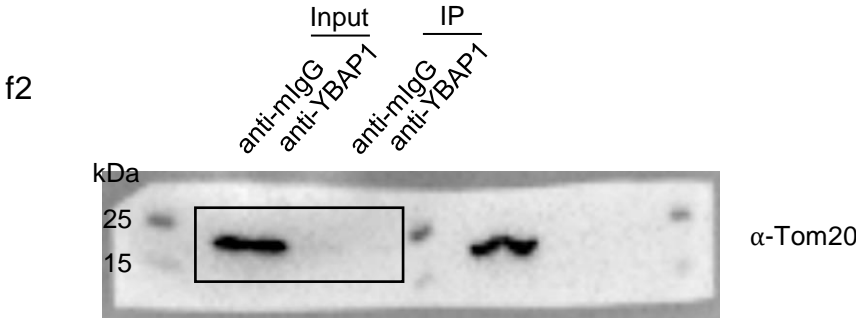

Supplement: Figure 4—source data 1. [file elife-85878-fig4-data1.zip › Figure 4f-source data 3.pdf]

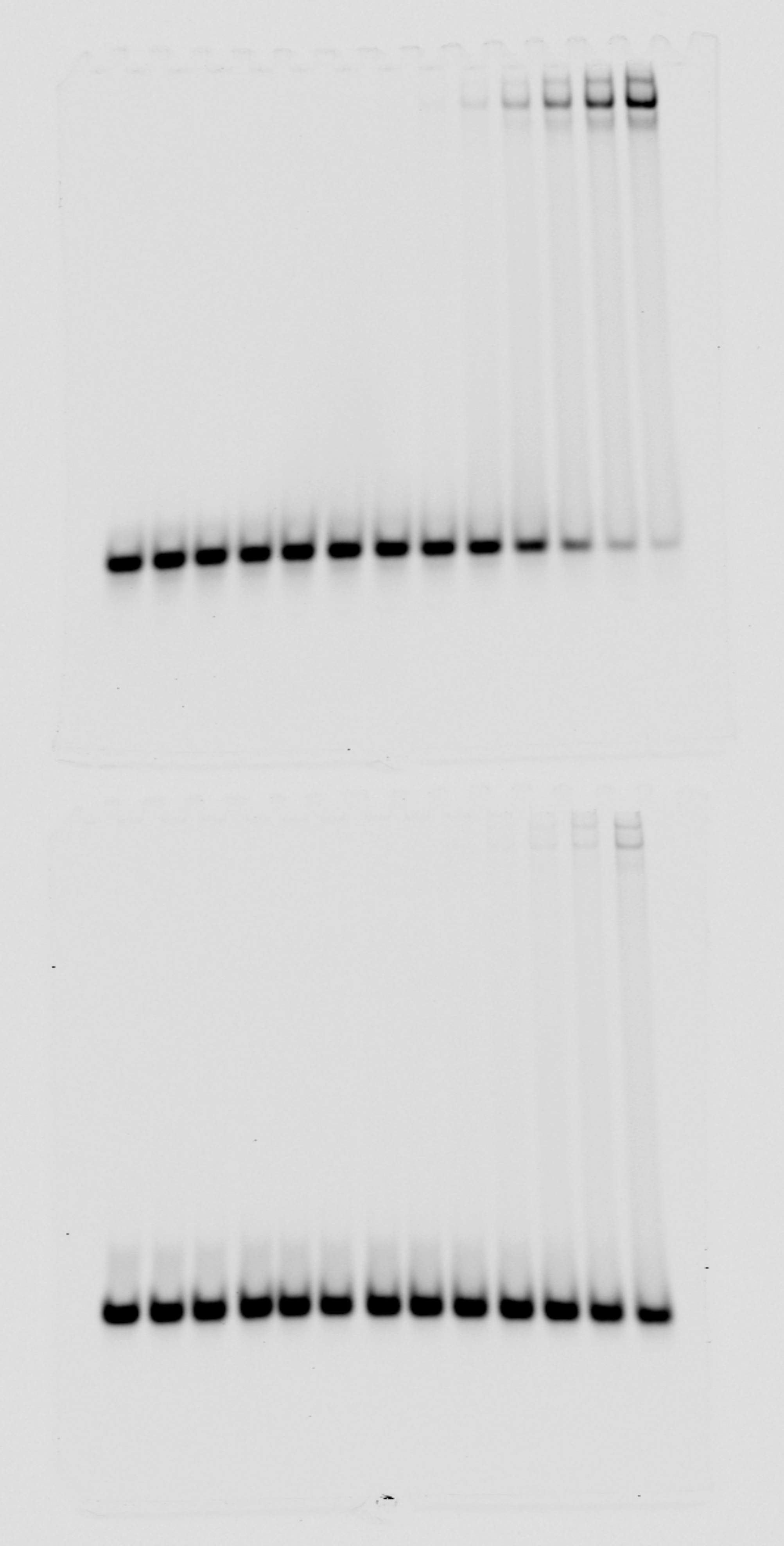

Supplement: Figure 4—source data 1. [file elife-85878-fig4-data1.zip › Figure 4h-source data 1.tif]

Figure 4h

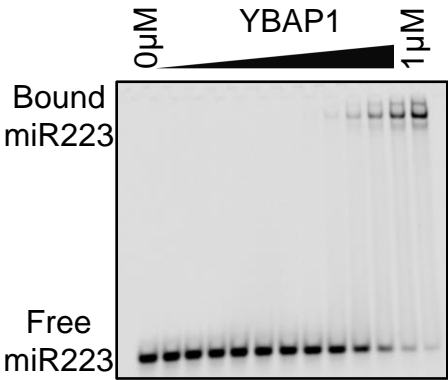

h1

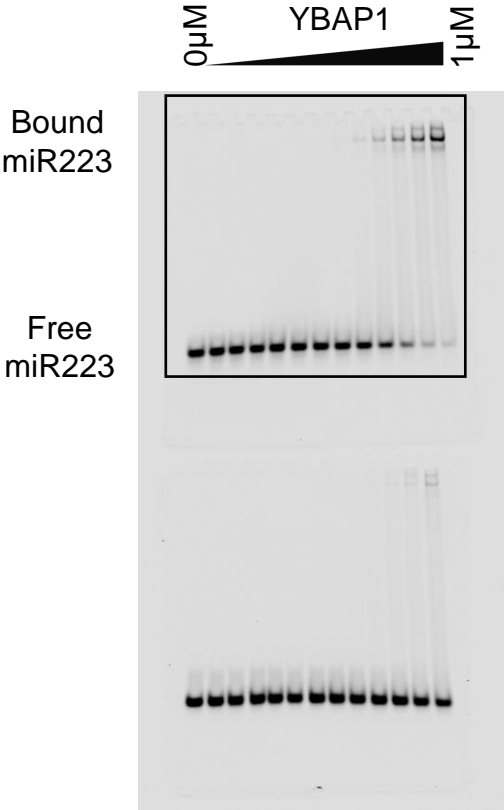

Supplement: Figure 4—source data 1. [file elife-85878-fig4-data1.zip › Figure 4h-source data 2.pdf]

Figure 4i

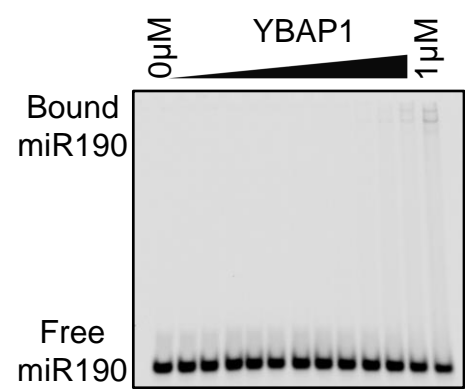

i1

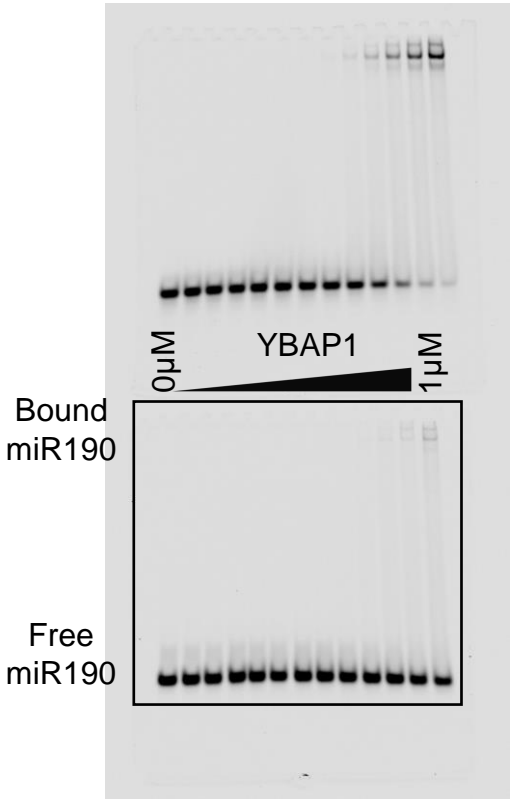

Supplement: Figure 4—source data 1. [file elife-85878-fig4-data1.zip › Figure 4i-source data 2.pdf]

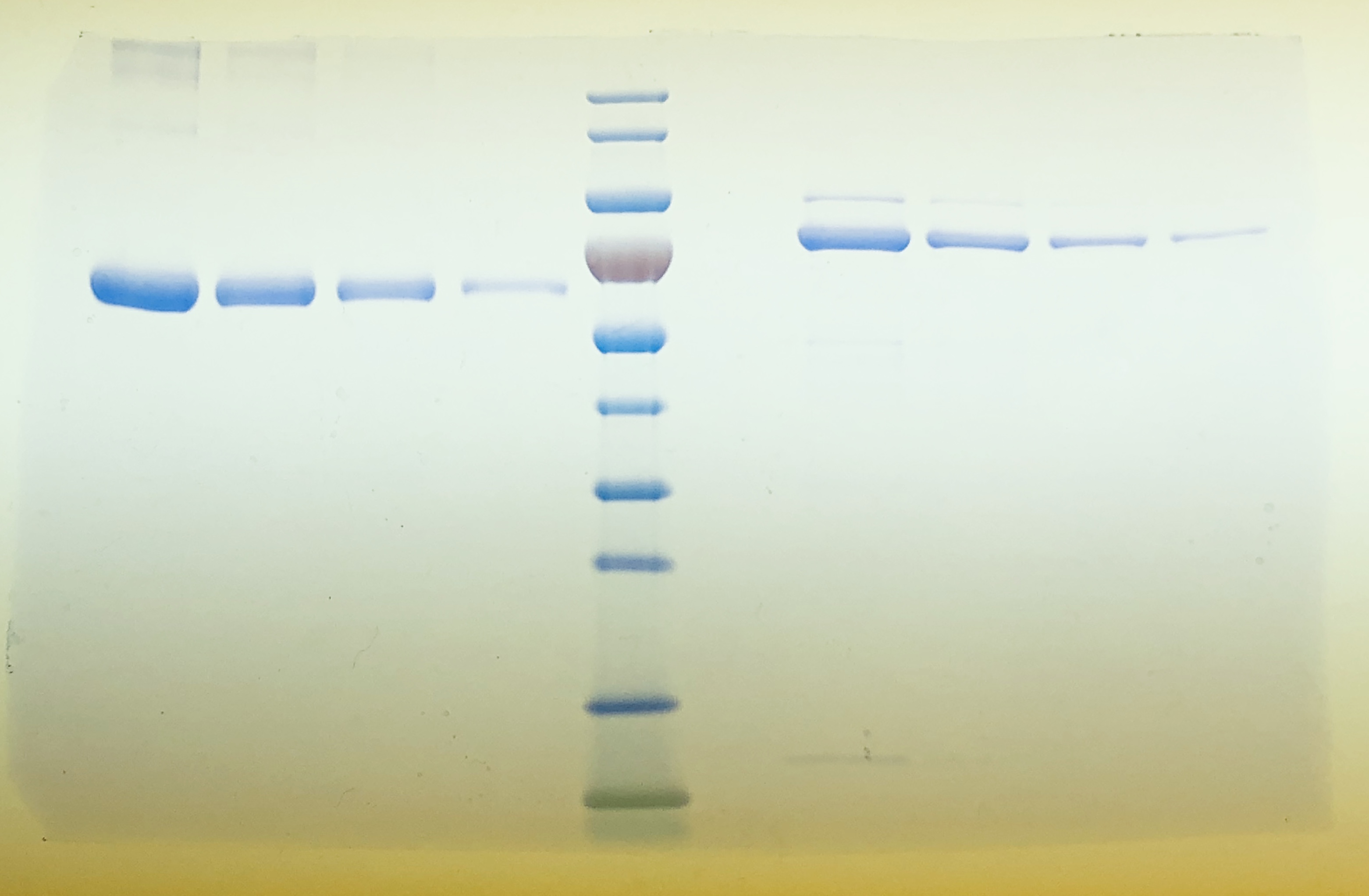

Supplement: Figure 4—figure supplement 1—source data 1. [file elife-85878-fig4-figsupp1-data1.zip › Figure 4-figure supplement 1a-source data 1.tif]

Figure 4-figure supplement 1a

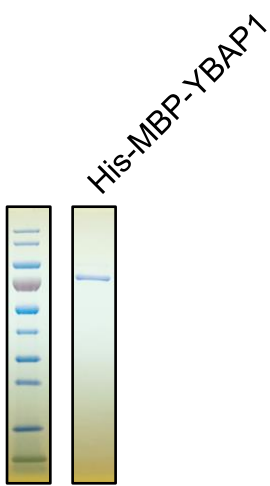

1a1.

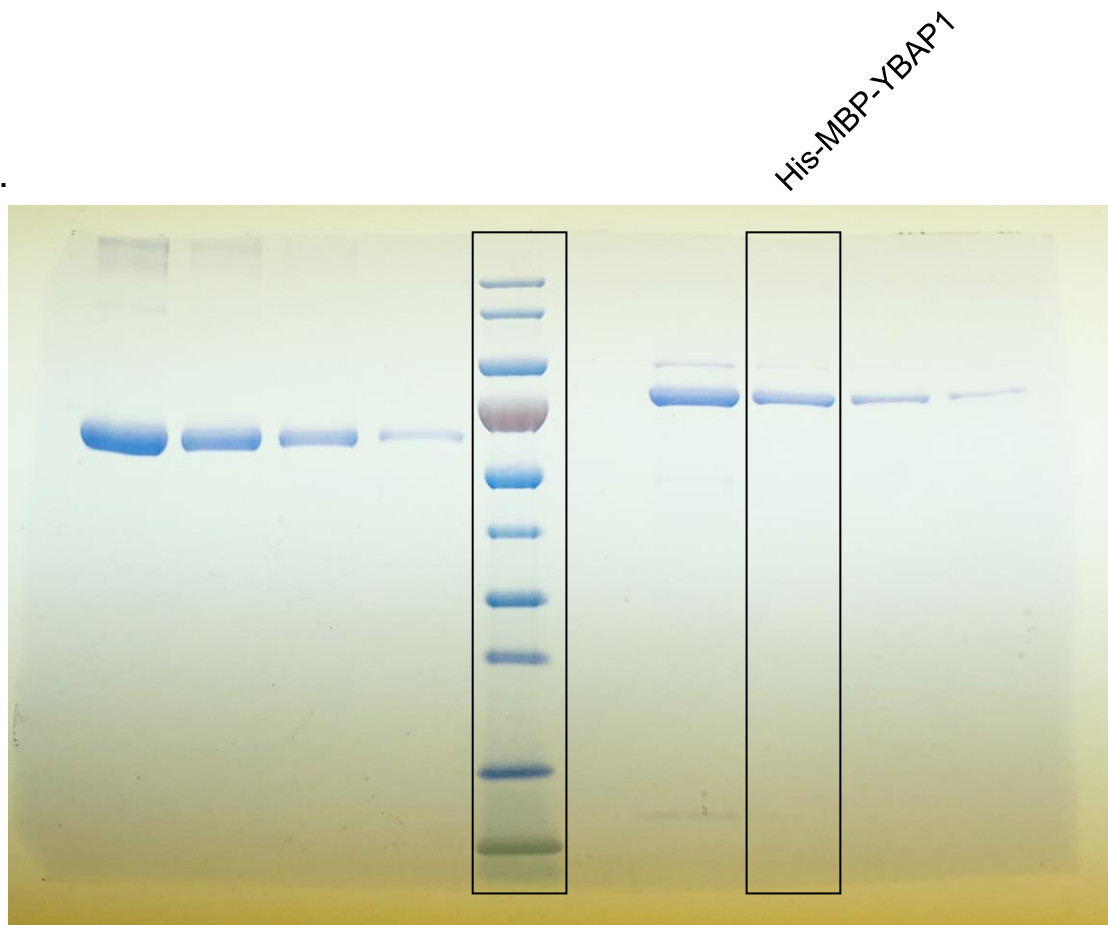

Supplement: Figure 4—figure supplement 1—source data 1. [file elife-85878-fig4-figsupp1-data1.zip › Figure 4-figure supplement 1a-source data 2.pdf]

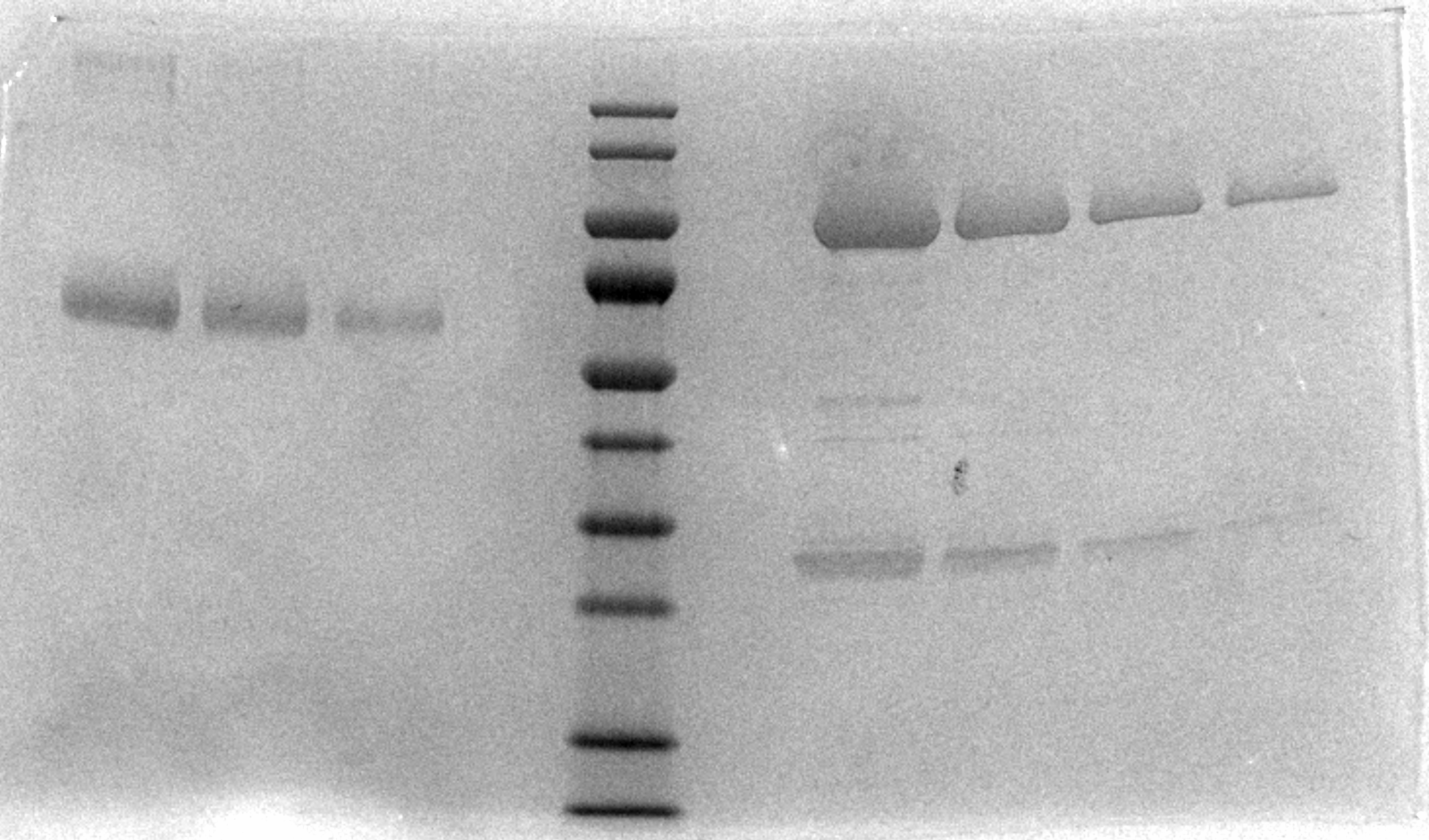

Supplement: Figure 4—figure supplement 1—source data 1. [file elife-85878-fig4-figsupp1-data1.zip › Figure 4-figure supplement 1b-source data 1.tif]

Figure 4-figure supplement 1b

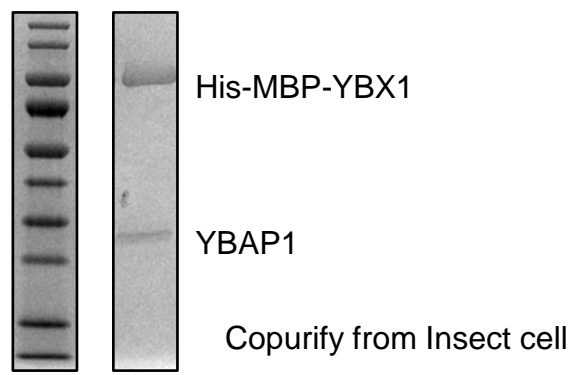

b1.

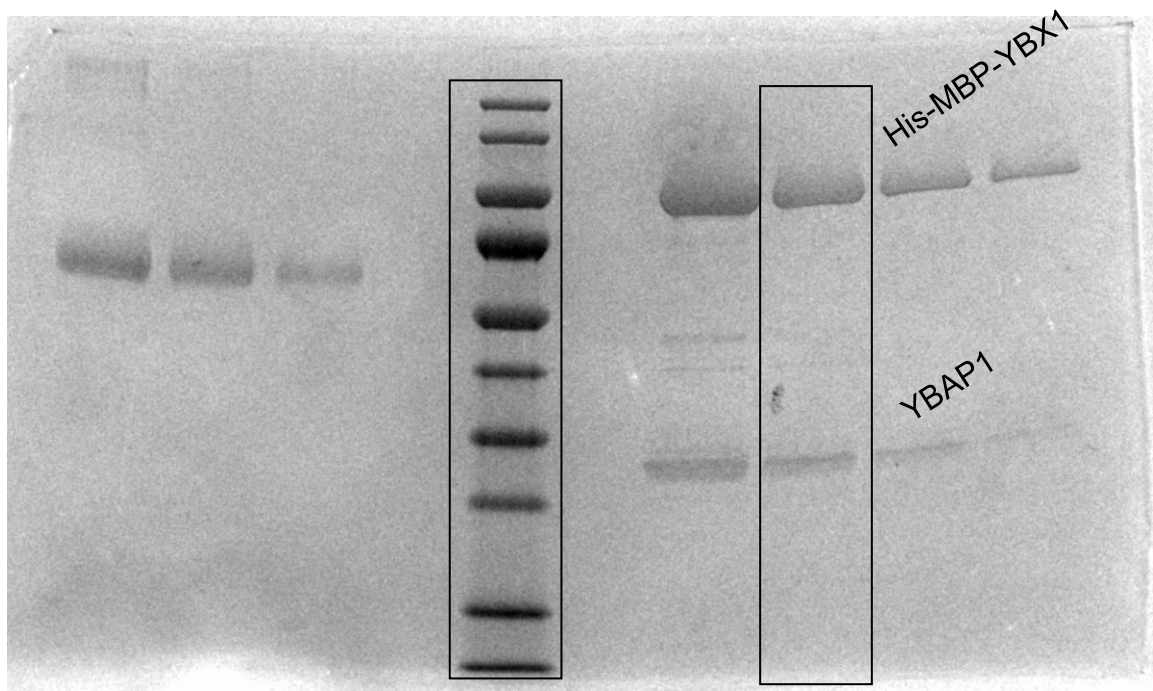

Supplement: Figure 4—figure supplement 1—source data 1. [file elife-85878-fig4-figsupp1-data1.zip › Figure 4-figure supplement 1b-source data 2.pdf]

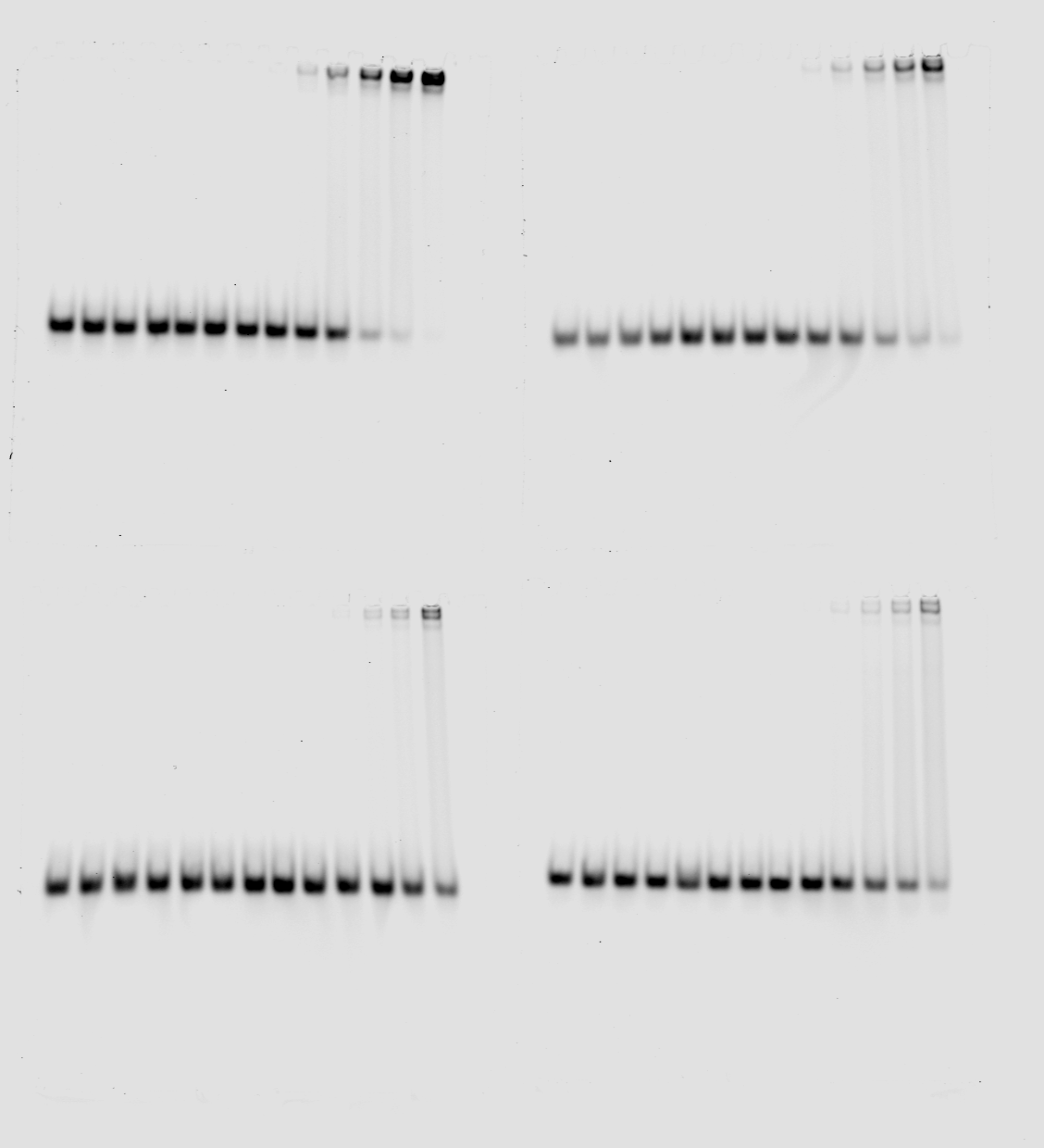

Supplement: Figure 4—figure supplement 2—source data 1. [file elife-85878-fig4-figsupp2-data1.zip › Figure 4-figure supplement 2a-source data 1.tif]

Figure 4-figure supplement 2a

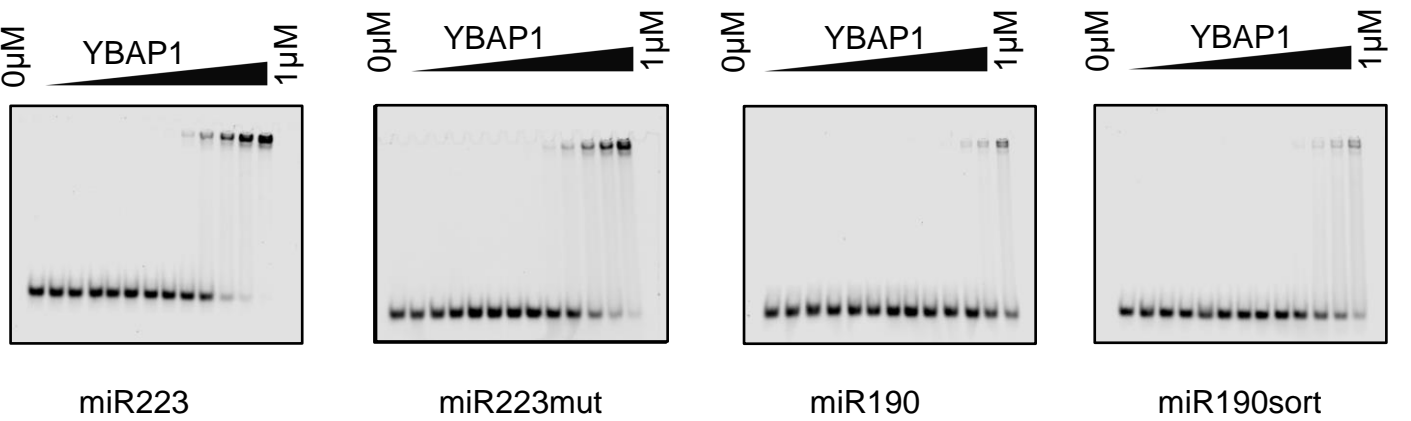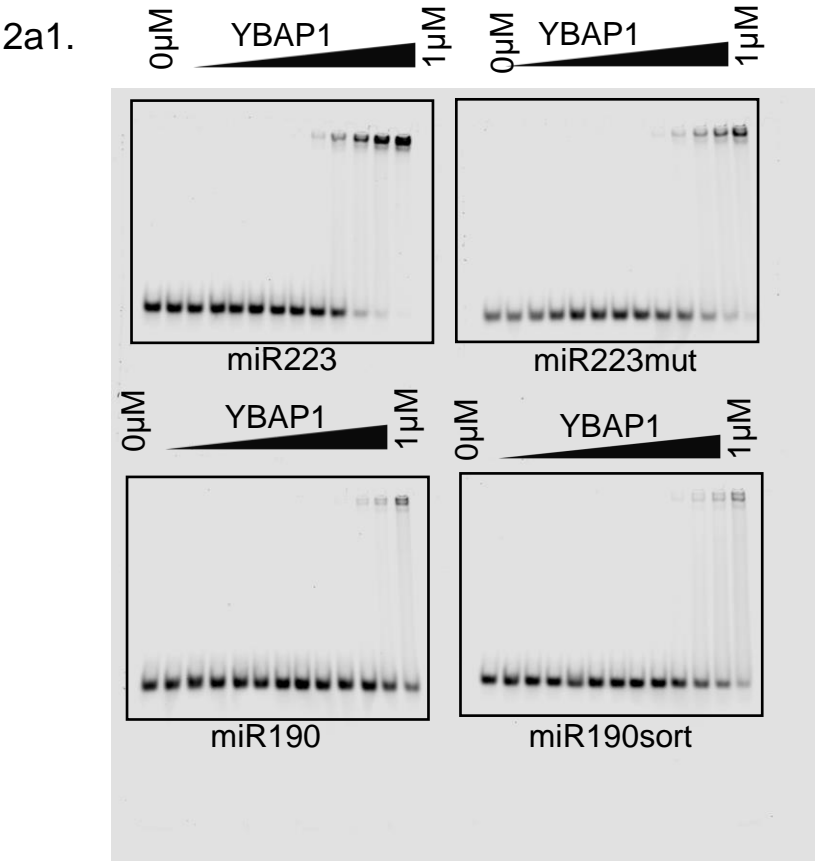

Supplement: Figure 4—figure supplement 2—source data 1. [file elife-85878-fig4-figsupp2-data1.zip › Figure 4-figure supplement 2a-source data 2.pdf]

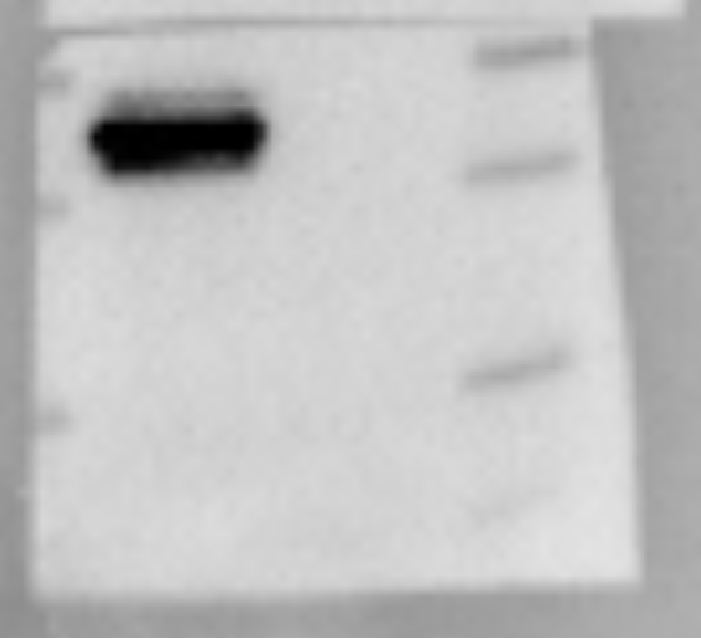

Supplement: Figure 5—source data 1. [file elife-85878-fig5-data1.zip › Figure 5b-source data 1-YBAP1.tif]

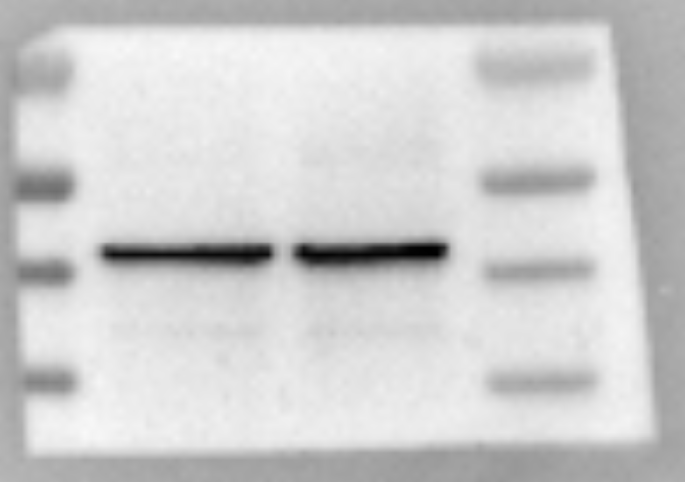

Supplement: Figure 5—source data 1. [file elife-85878-fig5-data1.zip › Figure 5b-source data 2-Citrate Synthase.tif]

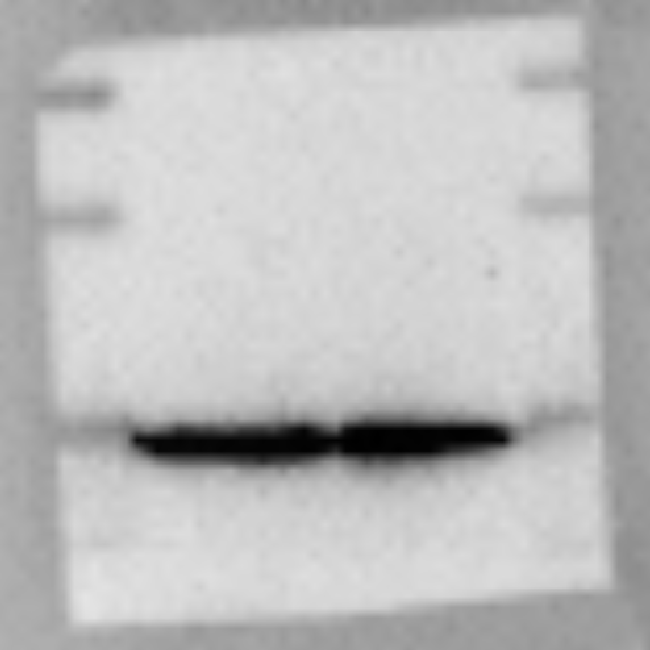

Supplement: Figure 5—source data 1. [file elife-85878-fig5-data1.zip › Figure 5b-source data 3-COX IV.tif]

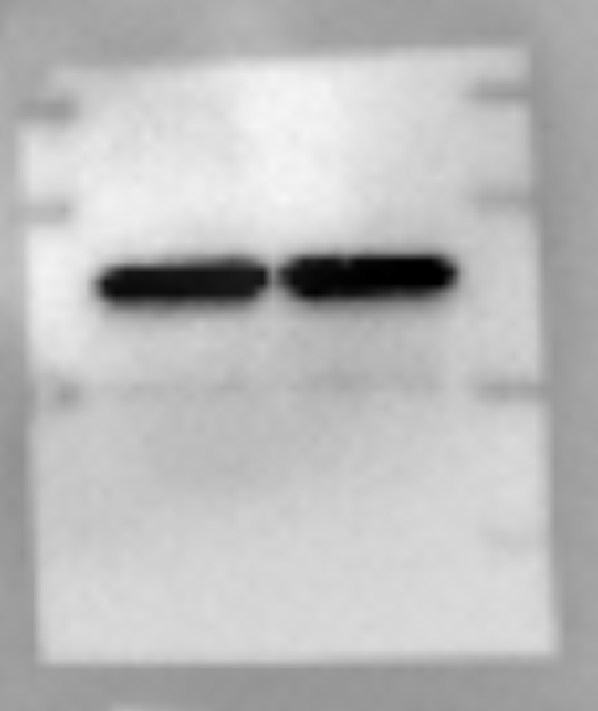

Supplement: Figure 5—source data 1. [file elife-85878-fig5-data1.zip › Figure 5b-source data 4-Tim23.tif]

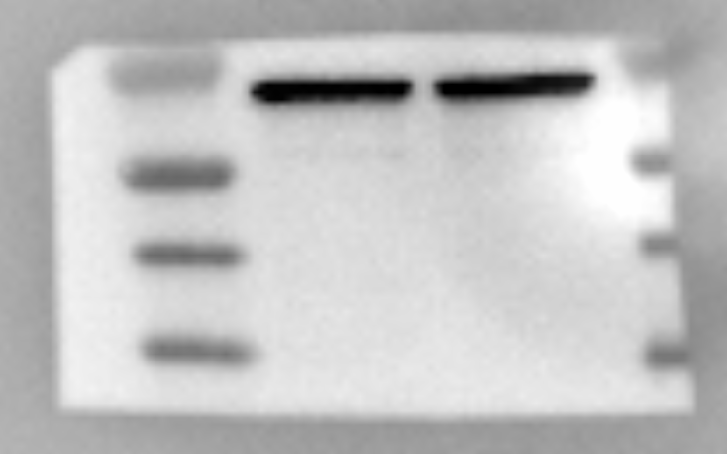

Supplement: Figure 5—source data 1. [file elife-85878-fig5-data1.zip › Figure 5b-source data 5-AIF.tif]

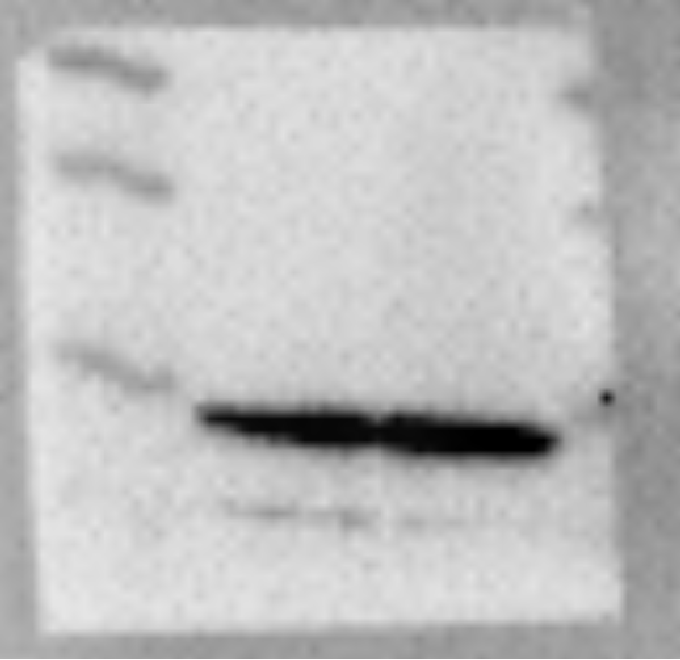

Supplement: Figure 5—source data 1. [file elife-85878-fig5-data1.zip › Figure 5b-source data 6-Tom20.tif]

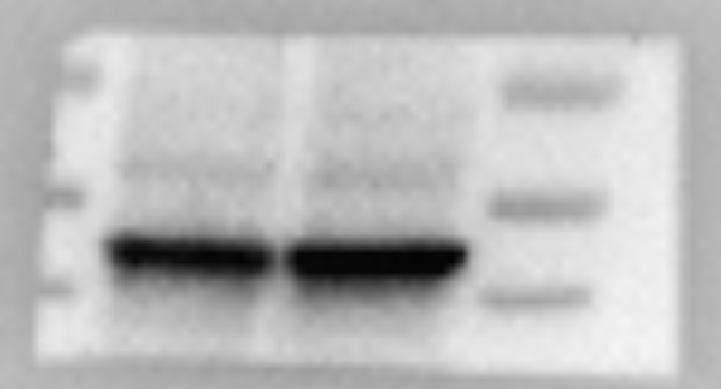

Supplement: Figure 5—source data 1. [file elife-85878-fig5-data1.zip › Figure 5b-source data 7-YBX1.tif]

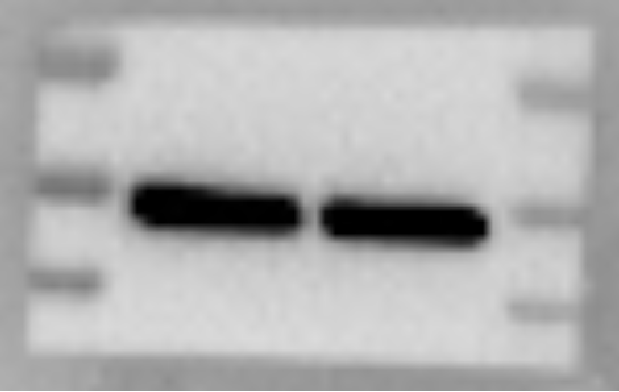

Supplement: Figure 5—source data 1. [file elife-85878-fig5-data1.zip › Figure 5b-source data 8-Tubulin.tif]

Figure 5b

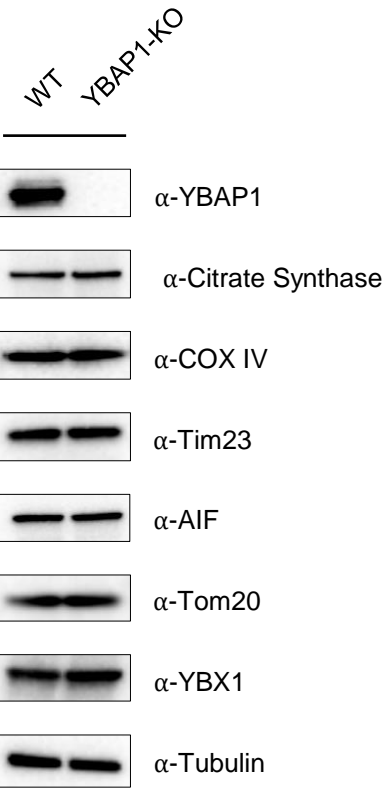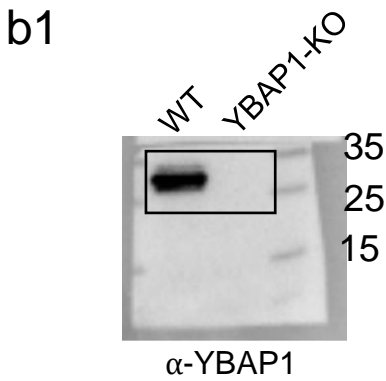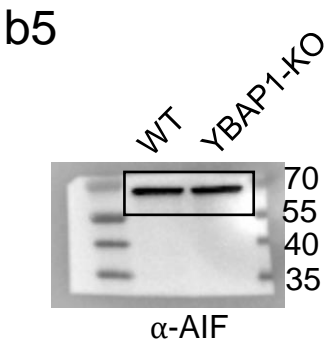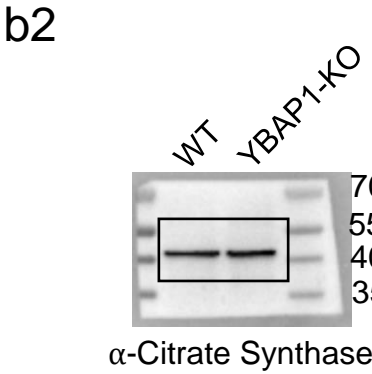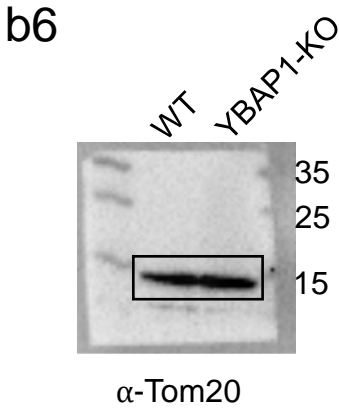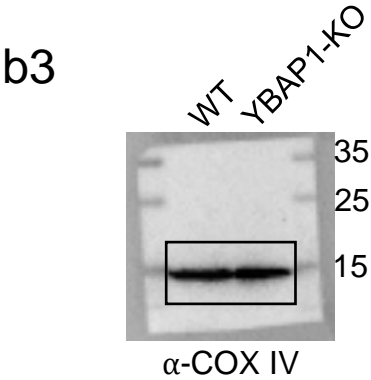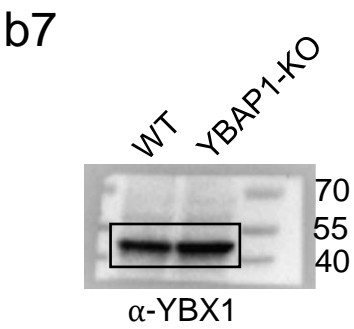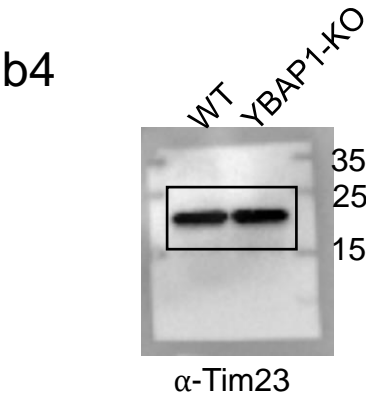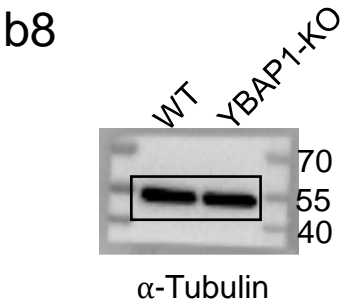

Supplement: Figure 5—source data 1. [file elife-85878-fig5-data1.zip › Figure 5b-source data 9.pdf]
